# Supplementary material for: The role of hospital managers in quality and patient safety: a systematic review
Source: BMJ Open. 2014 Sep 5;4(9):e005055. doi: 10.1136/bmjopen-2014-005055 (PMC4158193; doi:10.1136/bmjopen-2014-005055)
Supplement: Author's manuscript [file bmjopen-2014-005055.draft_revisions.pdf]

The Role of Hospital Managers in Quality and Patient Safety. A Systematic Review

Journal:	*BMJ Open*
Manuscript ID:	bmjopen-2014-005055
Article Type:	Research
Date Submitted by the Author:	13-Feb-2014
Complete List of Authors:	Parand, Anam; Imperial College London, Surgery and cancer<br>Dopson, Sue; University of Oxford, Said Business School<br>Renz, Anna; Imperial College London, Surgery and cancer<br>Vincent, Charles; University of Oxford, Experimental Psychology
**Primary Subject Heading**:	Medical management
Secondary Subject Heading:	Health services research
Keywords:	HEALTH SERVICES ADMINISTRATION & MANAGEMENT, Quality in health care < HEALTH SERVICES ADMINISTRATION & MANAGEMENT, Systematic literature review, Patient Safety, Leadership

SCHOLARONE™
Manuscripts

For peer review only

The Role of Hospital Managers in Quality and Patient Safety. A Systematic Review

For peer review only

Anam Parand[1], Sue Dopson[2], Anna Renz[1], Charles Vincent[3]

[1]Department of Surgery & Cancer, Imperial College London, London, UK
[2]Said Business School, University of Oxford, Oxford, UK
[3]Department of Experimental Psychology, University of Oxford, Oxford, UK

Correspondence to: Anam Parand, Department of Surgery & Cancer, Imperial College London, 503, 5th Floor Wright Fleming Building, St Mary's Campus, Norfolk Place, London W2 1PG, UK.
Email: a.parand@imperial.ac.uk; Tel: +44 (0) 207594 9726; Fax: +44 (0)20 7594 9726

Key Words: Quality of health care; Patient safety; Systematic literature review; Leadership, Management.

Abstract Word Count: 201 words

Manuscript Word Count: 4,330 words (excluding title page, abstract, references, acknowledgements and tables).

For peer review only - http://bmjopen.bmj.com/site/about/guidelines.xhtml

ABSTRACT

Objectives: To review the empirical literature to identify the activities, time spent and engagement of hospital managers in quality of care.

Design: A systematic review of the literature.

Methods: A search was carried out on the databases MEDLINE, PSYCHINFO, EMBASE, HMIC. The search strategy covered three facets: management, quality of care, and the hospital setting comprising medical subject headings and key terms. Reviewers screened 15,447 titles/abstracts and 423 full texts were checked against inclusion criteria. Data extraction and quality assessment were performed on 19 included articles.

Results: The evidence indicates that managers are involved in activities that are highly important to the quality and safety of their patients. Activities included strategy-setting and reporting, driving improvement culture and promotion of quality and safety. Significant positive associations with quality included setting the quality agenda and compensation attached to quality. However there is an inconsistency and inadequate employment of these conditions and actions across hospitals.

Conclusion: There is some evidence that managers' time spent and work can influence quality and safety clinical outcomes, processes and performance. However, there is a dearth of empirical studies on their work and its influence. We present a model to summarise the conditions and activities that affect quality performance.

For peer review only - http://bmjopen.bmj.com/site/about/guidelines.xhtml

ARTICLE SUMMARY

Strengths and limitations of this study

- This is the first systematic review of the literature that has considered the evidence on Boards' and managers' time spent, engagement and work within the context of quality and safety. This review adds to the predominantly commentary-based pieces that consider what managers should be doing by presenting what they are actually doing.
- The review reveals conditions and actions conducive to good quality management and offers a model to transparently present these to managers considering their own part in quality and safety.
- The search for this review has screened a vast amount of the literature (over 15,000 articles) across a number of databases.
- The small number of included studies and their varied study aims, design and population samples make generalisations difficult. With more literature on this topic, distinctions could be made between job positions.
- The quality assessment scores are subjective and may not take into consideration factors beyond the quality assessment scale used.

For peer review only

For peer review only - http://bmjopen.bmj.com/site/about/guidelines.xhtml

INTRODUCTION

Managers in healthcare have a legal and moral obligation to ensure a high quality of patient care and to strive to improve care. These managers are in a prime position to mandate policy, systems, procedures and organisational climates. Accordingly, many have argued that it is evident that healthcare managers possess an important and obvious role in quality of care and patient safety and that it is one of the highest priorities of healthcare managers.[1-3] In line with this, there have been calls for Boards to take responsibility for quality and safety outcomes.[4, 5] One article warned hospital leaders of the dangers of following in the path of bankers falling into recession, constrained by their lack of risk awareness and reluctance to take responsibility.[6] To add to the momentum are some high profile publicity of hospital management failures affecting quality and safety, eliciting strong instruction for managerial leadership for quality at the national level in some countries.[7, 8]

Beyond healthcare, there is clear evidence of managerial impact on workplace safety.[9-12] Within the literature on healthcare, there are non-empirical articles providing propositions and descriptions on managerial attitudes and efforts to improve safety and quality. This literature, made up of opinion articles, editorials and single participant experiences, present an array of insightful suggestions and recommendations for actions that hospital managers should take to improve the quality of patient care delivery in their organisation.[13-17] However, researchers have indicated that there is a limited evidence-base on this topic.[18-21] Others highlight the literature focus on the difficulties of the managers' role and the negative results of poor leadership on quality improvement (QI) rather than considering actions that managers presently undertake on quality and safety.[22, 23] Consequently, little is known about what healthcare managers are doing in practice to ensure and improve quality of care and patient safety, how much time they spend on this, and what research-based guidance is available for managers in order for them to decide on appropriate areas to become involved. Due perhaps to the broad nature of the topic, scientific studies exploring

For peer review only - http://bmjopen.bmj.com/site/about/guidelines.xhtml

these acts and their impact are likely to be a methodological challenge, although a systematic review of the evidence on this subject is notably absent. This present systematic literature review aims to identify empirical studies pertaining to the role of hospital managers in quality of care and patient safety. We define 'role' to comprise of managerial activities, time spent and active engagement in quality and safety and its improvement. Whilst the primary research question is on the managers' role, we take into consideration the contextual factors surrounding this role and its impact or importance as highlighted by the included studies. Our overarching question is 'What is the role of hospital managers in quality and safety and its improvement?' The specific review research questions are as follows:

- How much time is spent by hospital managers on quality and safety and its improvement?
- What are the managerial activities that relate to quality and safety and its improvement?
- How are managers engaged in quality and safety and its improvement?
- What impact do managers have on quality and safety and its improvement?
- How do contextual factors influence the managers' role and impact on quality and safety and its improvement?

METHODS

Concepts and definitions

Quality of care and patient safety were defined on the basis of widely accepted definitions from the Institute of Medicine [4, 24] and literature was searched for all key terms associated with quality and patient safety to produce an all encompassing approach. A manager was

For peer review only - http://bmjopen.bmj.com/site/about/guidelines.xhtml

defined as any employee that manages staff and is likely to hold managerial responsibilities such as budget responsibilities and staff recruitment and training. Therefore, all levels of managers including Boards of managers were included in this review with the exception of clinical frontline employees, e.g. doctors or nurses, who may have taken on further managerial responsibilities alongside their work but do not have a primary official role as a manager. Those that have specifically taken on a role for quality of care, e.g. the modern matron, were also excluded. Distinction between senior, middle and frontline management were as follows: senior management hold Trust-wide responsibilities;[25] middle managers are in the middle of the organisational hierarchy chart and have one or more managers reporting to them;[26] frontline managers are defined as managers at the first level of the organisational hierarchy chart who have frontline employees reporting to them. Board managers include all members of the Board. Although, there are overlaps between senior managers and Boards (for example Chief Executive Officers (CEOs) may sit on hospital Boards), we aim to present senior and Board level managers separately due to the differences in their responsibilities and position. Only managers that would manage within or govern hospitals were included, with the exclusion of settings that solely served mental health or that comprised solely of non-acute care community services. The definition of 'Role' focused on actual engagement, time spent and activities that do or did occur rather than those recommended that should or could occur.

Search Strategy

Literature was reviewed between 01 Jan 1983 and 01 Nov 2010. Eligible articles were those that described or tested managerial roles pertaining to quality and safety in the hospital setting. Part of the search strategy was based on guidance by Tanon et al (2010).[27] EMBASE, MEDLINE, Health Management Information Consortium (HMIC) and PSYCHINFO databases were searched. The search strategy involved three facets (i.Management;

For peer review only - http://bmjopen.bmj.com/site/about/guidelines.xhtml

ii.Quality; iii.Hospital Setting) and five steps. A facet on role was not included in the search strategy, as it would have significantly reduced the sensitivity of the search.

Multiple iterations and combinations of all search terms were tested to achieve the best level of specificity and sensitivity. In addition to the key terms, Medical Subject Headings (MeSH®) terms were used, which were ‘exploded’ to include all MeSH subheadings. All databases required slightly different MeSH terms (named Emtree in Embase), therefore four variations of the search strategies were used (see online Appendix 1 for the search strategies). Additional limits placed on the search strategy restricted study subjects to human and the language to English. The search strategy identified 15,447 articles after duplicates had been removed.

Screening

Three reviewers (AP, AR and DG) independently screened the titles and abstracts of the articles for studies that fit the inclusion criteria. One reviewer (AP) screened all 15,447 articles, while two additional reviewers screened 30% of the total sample retrieved from the search strategy: AR screened 20% and DG screened 10%. On testing inter-rater reliability, Cohen's kappa correlations showed low agreement between AR and AP (K=0.157, P<0.01) and between DG and AP (K=0.137, P<0.00).[28] However, there was a high percentage of agreement between raters (95% and 89% respectively), which reveals a good inter-rater reliability.[29, 30] Discrepancies were resolved by discussion and consensus. The main inclusion criteria were that: the setting was hospitals; the population sample reported on were managers; the context was quality and safety; the aim was to identify the managerial activities/time/engagement in quality and safety. The full inclusion/exclusion criteria and screening tool can be accessed in the online Appendices 2-3. Figure 1 presents the numbers of articles included and excluded at each stage of the review process.

For peer review only - http://bmjopen.bmj.com/site/about/guidelines.xhtml

Four hundred and twenty-three articles remained for full text screening. One reviewer (AP) screened all articles and a second reviewer (AR) reviewed 7% of these. A good agreement inter-rater reliability score was calculated (K=0.615,P<0.001) with 73% agreement. Hand searching and cross-referencing were carried out in case articles were missed by the search strategy or from restriction of databases. One additional article was identified from hand searching,[31] totalling 19 articles included in the systematic review.

For peer review only

For peer review only - http://bmjopen.bmj.com/site/about/guidelines.xhtml

Figure 1 Review stages based on PRISMA Flow Diagram[32]

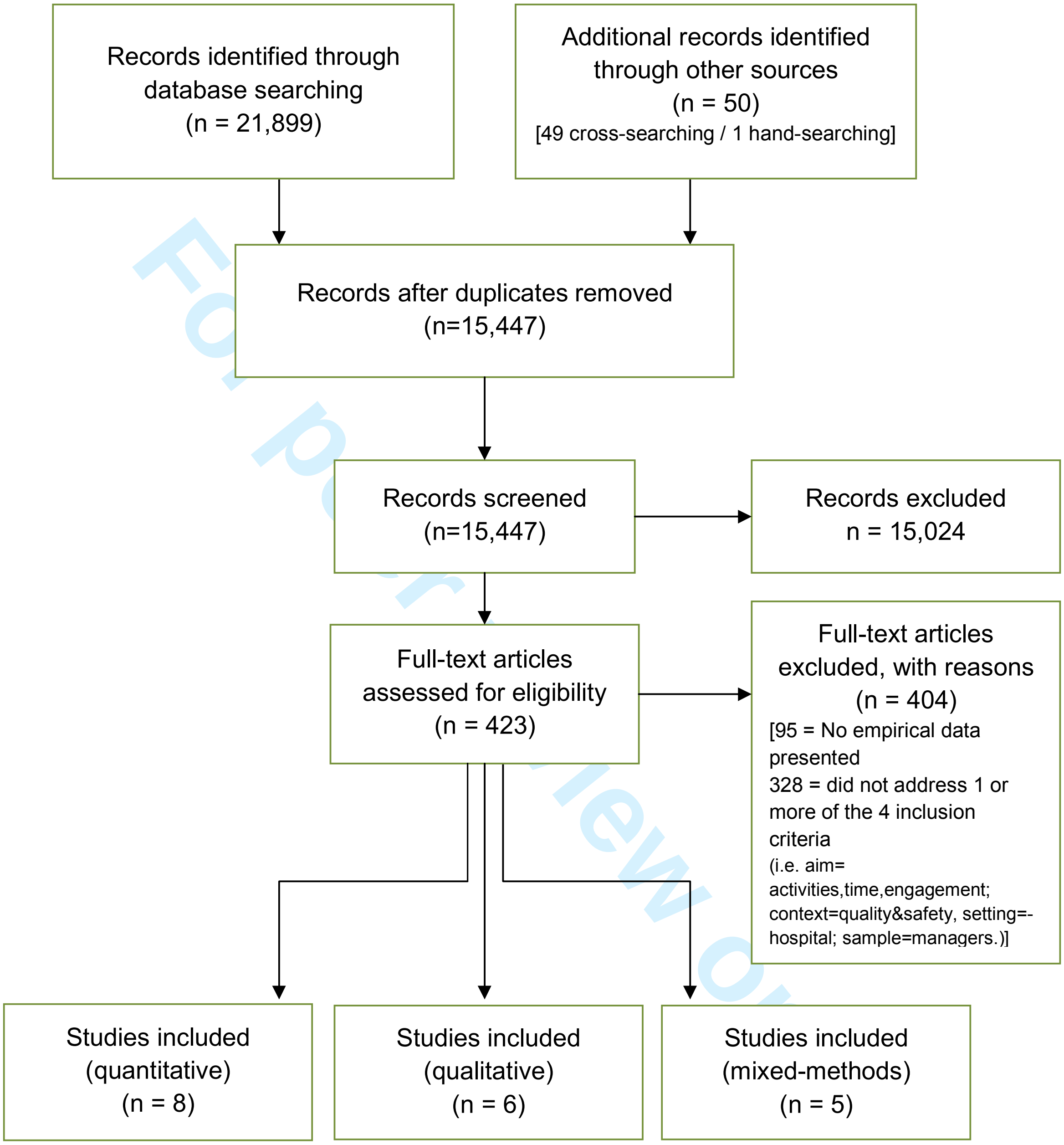

For peer review only - http://bmjopen.bmj.com/site/about/guidelines.xhtml

Data extraction & methodological quality

The characteristics and summary findings of the 19 included studies are presented in Table 1. This Table is a simplified version of a standardised template that was used to ensure consistency in data extracted from each article. Each study was assessed using a quality appraisal tool developed by Kmet et al (2004),[33] which comprised of two checklists (qualitative and quantitative). Random included articles (32%) were scored by AW for scoring consistency. All articles were scored on up to 24 questions with a score between 0-2, the total percentage scores are presented in Table 1. Some cumulative evidence bias may results from two larger datasets split into more than one study each.[34-37] Through a narrative synthesis, we aimed to maintain the original meanings, interpretations and raw data offered by the articles.[38]

RESULTS

This results section provides an overview description of the reviewed studies and their key findings. The findings are considered under four main headings: managerial time spent on quality and safety; managerial quality and safety activities; managerial impact on quality and safety; and contextual factors related to managers' quality and safety role. The section ends with a proposed model to summarise the review findings.

Description of the studies

From the 19 included studies, the majority were carried out and set in the US (14 studies), and investigated senior management and/or Boards (13 studies). Of these, 3 focused on senior managers alone (e.g. Chief Nursing Officers), 9 concentrated on Board managers and 1 included a mixture of managerial levels. Only 3 investigated middle managers (e.g. clinical directorate managers) and 3 examined frontline staff (e.g. unit nurse managers). The

For peer review only - http://bmjopen.bmj.com/site/about/guidelines.xhtml

settings of the study were mostly Trust or hospital-wide; a few articles were set in specific settings or contexts: elderly care,[39] evidence-based medicine,[40] staff productivity,[41] clinical risk management,[42] and hospital acquired infection prevention.[43] Two studies were involved in specific interventions,[44, 45] and 7 studies concentrated specifically on QI rather than quality and safety oversight or routine.[34, 39, 44-48] There were a mixture of 6 qualitative design (interviews or focus groups); 8 quantitative survey designs; and 5 mix-methods designs. The majority of authors opted for self-reports and all but one study employed a cross-sectional design.[45] The quality assessment scores ranged between 50-100%, with little use of verification procedures to establish credibility of interpretation and lack of reflexivity of qualitative accounts. Some quantitative results presented descriptives alone and failed to report variances.

Managerial time spent on quality and safety

The studies on Board level managers highlight an inadequate prioritisation of quality and patient safety on the Board agenda and subsequent time spent at Board meetings. Not all hospitals consistently have quality on their Board agenda, for example CEOs and chairpersons across 30 organisations reported that approximately a third of all Board meetings had quality on their agenda,[34] and necessary quality items were not consistently and sometimes never addressed.[35] In all studies examining time spent on quality and safety by the Board, less than half of the total time was spent on quality and safety,[31, 37, 47-50] with a majority of Boards spending 25% or less on quality.[31, 37, 48-50] Findings imply that this may be too low to have a positive influence on quality and safety, as higher quality performance was demonstrated by Boards that spent above 20%/25% on quality.[48, 49] Board members recognised that the usual time spent is insufficient.[47] However, few reported financial goals as more important than quality and safety goals,[31] and health system Boards only spent slightly more time on financial issues than quality.[50] Similar

For peer review only - http://bmjopen.bmj.com/site/about/guidelines.xhtml

For peer review only

issues are noted by studies on frontline managers; specifically that they placed less time and importance on QI,[41] identified as the least discussed topic by clinical managers.[51]

Managerial quality and safety activities

A broad range of quality-related activities were identified to be undertaken by managers. These are presented by the following three groupings: strategy-centred; data-centred and culture-centred.

Strategy-centred

Board priority-setting and planning strategies aligned with quality and safety goals were identified as Board managerial actions carried out in several studies. High percentages (over 80% in two studies) of Boards had formally established strategic goals for quality with specific targets, and aimed to create a quality plan integral to their broader strategic agenda.[31, 36] Contrary findings however suggest that the Board rarely set the agenda for the discussion on quality,[36] did not provide the ideas for their strategies,[31] and were largely uninvolved in strategic planning for QI.[47] In the latter case, the non-clinical Board managers felt that they held "passive" roles in quality decisions. This is important considering evidence that connects the activity of setting the hospital quality agenda with better performance in process of care and mortality.[37] Additionally, Boards that established goals in four areas of quality and publicly disseminated strategic goals and reported quality information were linked to high hospital performance.[34, 37, 49]

Culture-centred

Activities aimed at enhancing patient safety/QI culture emerged from several studies across organisational tiers.[43, 46, 47, 52] Board and senior management's activities included encouraging an organisational culture of QI on norms regarding interdepartmental/multi-

For peer review only - http://bmjopen.bmj.com/site/about/guidelines.xhtml

disciplinary collaboration and advocating QI efforts to clinicians and fellow senior managers, providing powerful messages of safety commitment and influencing the organisation's patient safety mission.[46, 52] Managers at differing levels focused on cultivating a culture of clinical excellence and articulating the organisational culture to staff.[43] Factors to motivate/engage middle and senior management in QI included senior management commitment, provision of resources and managerial role accountability.[39, 45] Findings revealed connections between senior management and Board priorities and values with hospital performance and on middle management quality-related activities. Ensuring capacity for high quality standards also appears within the remit of management, as physician credentialing was identified as a Board managers' responsibility in more than one study.[37, 47] From this review it is unclear to what degree Board involvement in the credentialing process has a significant impact on quality.[37, 40]

Data-centred

Information on quality and safety are continually supplied to the Board.[50] At all levels of management, activities around quality and safety data or information were recognised in 6 studies.[34, 37, 42, 44, 46, 52] Activities included collecting and collating information,[42] reviewing quality information,[34, 37, 52] using measures such as incident reports and infection rates to forge changes,[52] using patient satisfaction surveys,[34] taking corrective action based on adverse incidents or trends emphasised at Board meetings,[37] and providing feedback.[42, 46] The studies do not specify the changes made based on the data-related activities by senior managers; one study identified that frontline managers predominantly used data from an incident reporting tool to change policy/practice and training/education and communication between care providers.[44] However, overseeing data generally was found to be beneficial, as hospitals that carried out performance monitoring activities had significantly higher scores in process of care and lower mortality rates than hospitals that did not.[37]

For peer review only - http://bmjopen.bmj.com/site/about/guidelines.xhtml

For peer review only

Managerial impact on quality and safety outcomes

We have considered the associations found between specific managerial involvement and its affect on quality and safety. Here, we summarise the impact and importance of their general role. Of the articles that looked at either outcomes of management involvement in quality or at its perceived importance, 5 articles suggested that their role was beneficial to quality and safety performance.[31, 34, 37, 39, 48] Senior management support and engagement was identified as one of the primary factors associated with good hospital-wide quality outcomes and QI programme success.[34, 37, 39, 48] Conversely, 6 articles suggest that managers' involvement (from the Board, middle and frontline) has little, no or a negative influence on quality and safety.[31, 34, 37, 40, 41, 43, 48] Practices that showed no significant association with quality measures included Board's participation in physician credentialing.[34, 37] Another noted that if other champion leaders are present, management leadership was not deemed necessary.[43] Two articles identified a negative or inhibitory effect on evidence-based practices and staff productivity from frontline and middle managers.[40, 41]

Contextual factors related to managers' quality and safety role

Most of the articles focussed on issues that influenced the managers' role or their impact, as opposed to discussing the role of the managers. These provide an insight in to the types of conditions in which a manager can best undertake their role to affect quality and safety. Unfortunately it appears that many of these conditions are not in place.

Two studies found that a Board quality committee is a positive variable in quality performance, but that fewer than 60% had them.[37, 49] Similarly, compensation and performance evaluation linked to executive quality performance was identified in 4 articles [34, 36, 37, 48] and associated with better quality performance indicators,[37, 48] but quality

For peer review only - http://bmjopen.bmj.com/site/about/guidelines.xhtml

measures were insufficiently included in CEOs' performance evaluation.[34, 36] The use of the right measures to drive QI was raised in relation to Board managerial engagement in quality [34] and to impact on patient care improvement,[50] yet, almost half of this sample did not formally adopt system-wide measures and standards for quality. To aid them in these tasks, evidence indicates the common use of QI measure tools, such as a dashboard or scorecard, [36, 48, 49] with promising associations between dashboard use and quality outcomes.[37, 49]

Other factors linked to quality outcomes include management-staff relationship/high interactions between the Board and medical staff when setting quality strategy,[48] and managerial expertise. Although a connection between knowledge with quality outcomes was not found,[37] high performing hospitals have shown higher self-perceived ability to influence care, expertise at the Board and participation in training programs that have a quality component.[49] Disappointingly, there is a low level of CEO knowledge on quality and safety reports,[34] possibly little Boardroom awareness on salient nursing quality issues,[35] and little practice identified to improve quality literacy for the Board.[31, 36] There is however promise for new managers through relevant training at induction and by recruitment of those with relevant expertise.[31]

The Quality Management IPO Model

The input process output (IPO) model is a conceptual framework that helps to structure the review findings in a useful way, please see Figure 2.[53, 54] This literature may be conceptualised by considering what factors contribute (input) to managerial activities (process) that impact on quality and safety (output). The three factors are interrelated and interchangeable, presented by the cyclical interconnecting diagram.

For peer review only - http://bmjopen.bmj.com/site/about/guidelines.xhtml

First author; Year [Country]	Methods	Sample size [number of organisations]	Population sample [Level of management reported on (position of managers)]	Outcome Measure	Management Roles (Managerial quality and safety activities, time spent and engagement & key perceived importance and context factors)	Quality assessment score for qualitative studies	Quality assessment score for quantitative studies
Baker et al, 2010 [Canada][31]	Mixed methods (interviews, case studies, surveys)	n=15 interviews; n=4 Board case studies; n=79 surveys [79 organisations]	Managers [Board management]	Perceptions of managers on management Board practices in quality and safety	• Less than half (43%) of Boards reported that they addressed quality and patient safety issues in all meetings<br>• One-third of Boards spend 25% of their time or more on quality and patient safety issues.<br>• More than 80% of Boards have formally established strategic goals for quality with specific targets, but a majority of Board chairs indicate that their Boards did not provide the ideas for strategic direction or initiatives.<br>• Board chairs reported a low participation in education on quality and safety: 43% reported that all the Board members participated, 19% stated that more than half participated and 23% said it was less than a quarter of the Board<br>• Most Board chairs (87%) reported Board member induction training on responsibilities for quality and safety, although almost a third (30%) reported few or no opportunities for education on this, 42% reported some opportunities and 28% reported many).<br>• Approximately half (57%) of the Board chairs acknowledged recruitment of individuals that have knowledge, skills and experience in quality and patient safety onto the Board. A Board skills matrix included quality and safety as one of the competency areas.	16/20 (80%)	12/22 (55%)
Balding 2005 [Australia][45]	Mixed methods (action research, surveys & focus groups)	n=35 [1 hospital]	Managers [Middle management (Nursing managers and allied health managers)]	Perceptions of managers on their engagement in a QI programme	Five elements deemed essential to middle manager engagement:<br>• (1) Senior management commitment and leadership (e.g. senior management provides strategic direction for QI plan)<br>• (2) Provision of resources and opportunities for QI education and information dissemination (e.g. basic QI skills provided to all staff)<br>• (3) Senior and middle manager role accountability (e.g.: senior managers and middle managers agree QI roles and expectations)<br>• (4) Middle manager involvement in QI planning (e.g. senior and middle managers plan together)<br>• (5) Middle managers own and operate QI program (e.g. ongoing review and evaluation of the progress of the QI program by the middle and senior managers)	14/20 (70%)	15/22 (68%)

For peer review only - http://bmjopen.bmj.com/site/about/guidelines.xhtml

Bradely et al, 2003 [US][46]	Qualitative (interviews)	n=45 [8 hospitals]	Clinical staff and senior management [Senior management (unspecified)]	Perceptions of roles and activities that comprise senior management's involvement in quality improvement efforts	Five common roles and activities that captured the variation in management involvement in quality improvement efforts:<br>• (1) Personal engagement of senior managers<br>• (2) Management's relationship with clinical staff<br>• (3) Promotion of an organisational culture of quality improvement<br>• (4) Support of quality improvement with organisational structures<br>• (5) Procurement of organisational resources for quality improvement efforts	19/20 (95%)	NA
Bradely et al, 2006 [US][39]	Mixed-methods (surveys & interviews)	n=63 survey respondents [63 hospitals]; n=102 interviewees [13 hospitals]	Managers [Senior management (chief operating officer, vice president, medical director, chief nursing officer, director of volunteers, program director)]	Predominantly self-reported perceptions management-related factors around the HELP programme	• Providing resources for needed staffing or staff training<br>• Promoting the program among the governing Board, physicians who were initially less involved, and other administrators<br>• Senior management support reported as the primary enabling factor in the implementation of such programmes (96.6%), along with a lack of support as the primary reason for not implementing the programme (65.0%)<br>• The interviews supported that having an administrative champion was considered essential to their program's success	19/20 (95%)	17/22 (77%)
Braithwaite et al, 2004 [Australia][51]	Mixed methods (ethnographic work, observations & focus groups)	n=64 managers in focus groups [1 hospital]; Ethnographic case studies and n=4 observed [2 hospitals]	Managers [Frontline management (Medical managers, nurse managers & allied health managers)]	Observations and self-reported perceptions of clinician-managers' activities	• Quality was the least discussed topic (e.g. Continuous quality improvement)<br>• The most discussed topic was people (e.g. staffing, delegating) and organisational issues, e.g. beds and, equipment	16/20 (80%)	NA

For peer review only

For peer review only - http://bmjopen.bmj.com/site/about/guidelines.xhtml

Caine & Kenwrick, 1997[UK][40]	Qualitative (interviews)	n=10 [2 hospitals]	Managers [Middle management (Clinical directorate managers)]	Perceptions of managers on the managers' role in facilitating evidence-based practice in their nursing teams	• Managers saw their role in research implementation as a facilitator, ensuring quality and financial objectives and standards were met<br>• Managers perceived their facilitatory behaviours produced a low level of clinical change.<br>• Managers are not actively advocating research-based practice and failing to integrate it into everyday practice. Their behaviour inhibited the development of evidence-based nursing practice<br>• Devolved responsibility of use of research to individual professionals	14/20 (70%)	NA
Fox, Fox & Wells 1999 [US][41]	Quantitative (surveys &self kept activity logs)	n=16 [1 hospital]	Managers [Frontline management (Nurse administrative managers (NAMs))]	Perceptions of managers on their activities impacting unit personnel productivity	• The small amount of total management allocated to QI (2.6%) was the least time spent of all management functions<br>• A negative relationship between time spent in QI activities and unit personnel productivity. An increase (from 2.5% to 5%) in QI time/effort by NAMs would reduce staff productivity significantly by approximately 8%.<br>• The greater the experiences of NAMs as managers, the more time spent on QI. These seasoned NAMs spent more time on monitoring, reporting QI results, and quality improvement teams (statistics nor provided)	NA	13/22 (59%)
Harris, 2000 [UK][42]	Quantitative (surveys)	n=42 [42 hospitals]	Managers [Middle management (Nurse managers)]	Perceptions of managers on managers' quality and safety practices	• The majority of managers (91%) who received collated incident information used it to feed back to their own staff. 60% always fed back to staff, 28% sometimes did, 2% never did.<br>• Of the Trusts that had written guidance on types of clinical incident to report, 80% of managers had general guidance and fewer (20%) had written speciality specific guidance.<br>• 76% of managers reported information collation of clinical incidents. Of these, 59% were involved in data collection themselves.	NA	13/22 (59%)

For peer review only

Jha and Epstein, 2010 [US][49]	Quantitative (surveys)	n=722 [767 hospitals]	Managers [Board]	Perceptions of managers on the role of managers in quality and safety & quality outcome measurement (from Hospital Quality Alliance) i.e. 19 practices for care in 3 clinical conditions	• Two-thirds (63%) of Boards had quality as an agenda item at every meeting<br>• Fewer than half (42%) of the hospitals spent at least 20% of the Board's time on clinical quality<br>• 72% of Boards regularly reviewed a quality dashboard<br>• Most respondents reported that their Boards had established, endorsed, or approved goals in four areas of quality: hospital-acquired infections (82%), medication errors (83%), the HQA/Joint commission core measures (72%), and patient satisfaction (91%)<br>High-performing hospitals were more likely than low-performing hospitals to have:<br>• Board reviews of a quality dashboard regularly (<0.001) & of clinical measures (all <0.05)<br>• Quality performance on the agenda at every Board meeting (0.003)<br>• At least 20% of Board time on clinical quality (0.001)<br>• Has a quality subcommittee (0.001)	NA	22/22 (100%)
Jiang et al, 2008 [US][36]	Quantitative (surveys)	n=562 [387 hospitals]	Managers [Board & Senior management (presidents/ CEOs)]	Perceptions of managers on managers' practices in quality and safety; and outcomes of care (composite scores of risk-adjusted mortality indicators)	• 75% of CEOs reported that most to all of the Board meetings have a specific agenda item devoted to quality. Only 41% indicated that the Boards spend more than 20 percent of its meeting time on the specific item of quality.<br>The following activities were most reported to be performed:<br>• Board establishing strategic goals for QI (81.3%)<br>• Use quality dashboards to track performance (86%)<br>• Follow up corrective actions related to adverse events (83%)<br>The following activities were least reported to be performed:<br>• Board involvement in setting the agenda for the discussion on quality (42.4%)<br>• Inclusion of the quality measures in the CEO's performance evaluation (54.6%)<br>• Improvement of quality literacy of Board members (48.9%)<br>• Board written policy on quality and formally communicated it (30.8%)	NA	20/26 (77%)
Jiang et al, 2009 [US][37]	Quantitative (surveys)	n=490 [490 hospitals]	Managers [Board & Senior management (CEOS & Hospital presidents reports)]	Perceptions of managers on manager's practices in quality and safety; and process of care measures (20 measures in 4 clinical areas); and outcome measures (composite scores of risk-adjusted mortality indicators)	Board practices found to be associated with better performance (all P<0.05) in process of care (POC) and adjusted mortality (M) included:<br>• Having a Board quality committee (83.8%POC, 6.2M versus 80.2%POC, 7.9M without a committee)<br>• Establishing strategic goals for quality improvement (82.8%POC, 6.6M versus 80.3%POC, 7.9M)<br>• Being involved in setting the quality agenda for the hospital (83.2%POC, 6.4M versus 80.9%POC, 7.7M)<br>• Including a specific item on quality in Board meetings (83.2%POC, 6.5M versus 78.5%POC, 8.6M)<br>• Using a dashboard with national benchmarks and internal data that includes indicators for clinical quality, patient safety, and patient satisfaction (all above 80%POC & below 6.5M versus all below 80%POC and above 7M)<br>• Linking senior executives' performance evaluation to quality and patient safety indicators (83.1%POC, 6.6M versus 80.4%POC, 7.6M)<br>Practices that did NOT show significant association with the quality measures for process and mortality include:<br>• Reporting to the Board of any corrective action related to adverse events (82.5%POC, 7.0M versus 81.8%POC, 6.6M)	NA	22/24 (92%)

For peer review only

For peer review only - http://bmjopen.bmj.com/site/about/guidelines.xhtml

					• Board's participation in physician credentialing (82.8%POC, 6.9M versus 81.5%POC, 6.9M)<br>• Orientation for new Board members on quality(82.9%POC, 6.8M versus 81.7%POC, 7.0M)<br>• Education of Board members on quality issues (82.8%POC, 7.0M versus 81.9%POC, 6.9M)		
Joshi & Hines, 2006 [US][34]	Mixed-methods (surveys & interviews)	n=37 survey respondents; n=47 interviewees [30 hospitals]	Managers [Board & Senior management (CEOs, Board chairs)]	Perceptions of managers on managers' practices in quality and safety and appropriate care measure (ACM) and risk adjusted mortality.	• Board engagement in quality was reported as satisfactory (7.58 by CEOs and 8.10 by Chairs on a 1-10 scale where 10 indicates greatest satisfaction)<br>• Board engagement was positively associated with perceptions of the rate of progress in improvement (r=.44, p = .05), and marginally associated with ACM scores (r=.41, p=.07)<br>• Approximately one third of Board meetings are devoted to discussing quality issues (reported at 35% by CEOs and 27% by Chairs)<br>• Integrating Quality Planning and Strategic Planning was also rated as satisfactory (7.67 by CEOs and 8.85 by Chairs)<br>• Approximately two-thirds of respondents reported using patient satisfaction surveys (70% and 65% reported by CEOs and Chairs respectively)<br>• Low level of CEO expertise in QI, as reported by themselves (2.70) and by Board Chairs (3.35%) on a scale of 1-10 where 1 is low familiarity and 10 is high familiarity.	12/20 (60%)	16/20 (80%)
Levey et al, 2007 [US][47]	Qualitative (Interviews)	n=96 [18 hospitals]	Managers [Board & Senior management (Hospital Board members, CEOs, Chief Medical Officers, Chief Quality Officers, medical staff leaders)]	Perceptions of managers on managers' role in quality and safety	• Few CEOs were willing to take the lead for transformation to a "culture of quality"<br>• Board members were largely uninvolved in strategic planning for QI<br>• In terms of the Board's quality functions, respondents largely agreed that physician credentialing was their critical responsibility<br>• Non-physicians reported that they felt relegated to "passive" roles in decisions on quality and seemed reluctant to assume leadership roles in the quality domain.<br>• Board meeting agendas maintained a focus on financial issues, although patient safety/care and QI were gaining prominence<br>• About half of the respondents said that quality was not sufficiently highlighted during meetings. Estimates of time devoted to quality and safety issues at Board meetings were between 10% to 35%	13/20 (65%)	NA
Mastal, Joshi & Shulke 2007 [US][35]	Qualitative (interviews and a focus group)	n=73 interviewees; 1 focus group [63 hospitals]	Managers [Board & Senior management (Board chairs, CEOs, Chief Nurse Officers)]	Perceptions of managers on managers' role in quality and safety	• Two chief nursing officers (CNOs) reported that nursing quality was never addressed at Board meetings.<br>• Few of the CNOs, CEOs, and Board chairs responded that issues are discussed more frequently, such as at every meeting.<br>• Quality and patient safety measures for nurses are not consistently addressed during all hospital Board meetings.<br>• Staffing concerns are the most frequent measure of nursing quality reported at the Board level	12/20 (60%)	NA

For peer review only - http://bmjopen.bmj.com/site/about/guidelines.xhtml

Poniatowski, Stanley & Youngberg, 2005 [US][44]	Quantitative (surveys)	n=515 [16 academic medical centers]	Managers [Frontline management (Unit Nurse managers)]	Perceptions of managers on their practices with Patient Safety Net (PSN)	• Managers reviewed on average 65% of the PSN events reported<br>As a result of what was learned from PSN data, 162 managers detailed their changes made to:<br>• Policies and practices (59%)<br>• Training, education and communication between care providers (27%)<br>• Purchase of new equipment and supplies (8%)<br>• Staffing (6%)	N/A	10/20 (50%)
Prybil et al, 2010(US)[50]	Quantitative (surveys)	n=123 [712 hospitals]	Managers [Board & Senior management (CEOs & Boards)]	Perceptions of managers on their role in quality and safety	• Health system Boards spent 23% of their Board meeting time on quality and safety issues. They only spent slightly more on financial issues (25.2%) and strategic planning (27.2%)<br>• Almost all (96%) CEOs said that the Boards regularly received formal written reports on quality targets<br>• 88% of CEOs said that the Boards had assigned quality and safety oversight to a standing Board committee<br>• All but one (98.9%) of the CEOs stated that they have specific performance expectations and criteria related to quality and safety<br>• CEOs reported 59% of the Boards formally adopted system-wide measures and standards for quality	N/A	14/22 (64%)
Saint et al, 2010 [US][43]	Qualitative (interviews)	n=86 (interviewees) [14 hospitals]	Senior hospital staff and managers [Mixed levels (Nurse managers, chief physicians, Chairs of medicine, Chief of Staffs, hospital directors, CEOs & clinical non-managerial staff)]	Perceptions of managers on managers' practices in healthcare-associated infection (HAI)	• Although committed leadership by CEOs can be helpful, it was not always necessary, provided that other hospital leaders were committed to infection prevention<br>Behaviours of leaders who successfully implemented/facilitated practices to prevent HAI:<br>• Cultivated a culture of clinical excellence and kept their eye on improving patient care<br>• Developed a vision<br>• Articulated the organisational culture well and conveyed that to staff at all levels.<br>• Focused on overcoming barriers and dealing directly with resistant staff or process issues that impeded prevention of HAI<br>• Cultivated leadership skills and inspired the people they supervised (motivating and energising them to work towards the goal of preventing HAI)<br>• Thought strategically while acting locally; planned ahead and left few things to chance<br>• They did the politicking before issues arose for committee votes<br>• They leveraged personal prestige to move initiatives forward<br>• They worked well across disciplines	16/20 (80%)	NA

For peer review only - http://bmjopen.bmj.com/site/about/guidelines.xhtml

Vaughn et al, 2006 (US)[48]	Quantitative (surveys)	n=413 [413 hospitals]	Managers [Board & Senior management (chief executives & senior quality executives; Board, executives, clinical leadership)]	Perceptions of managers on managers' role in QI & observed hospital Quality index outcomes (risk-adjusted measures of morbidity, mortality, and medical complications)	• 72% of hospital Boards spent one-quarter of their time or less on quality-of-care issues. About 5% of Boards spent more than half of their time on these issues<br>• A majority of respondents reported great influence from government and regulatory agencies (87%), consumers (72%) and accrediting bodies (74%) on quality priorities. Although 44% of respondents also noted that multiple government and regulatory requirements were unhelpful<br>Better quality index scores (QIS) are associated with hospitals where the Board:<br>• Spends more than 25% of their time on quality issues (QIS 83 – QIS mean 100 across hospitals)<br>• Receives a formal quality performance measurement report (QIS 302)<br>• Bases the senior executives' compensation in part on QI performance (QIS 239)<br>• Engages in a great amount of interaction with the medical staff on quality strategy	NA	21/22 (95%)
Weingart & Page, 2004 [US][52]	Qualitative (case study documentation analysis and meeting discussions and focus group)	n=30 [10 hospitals and other stakeholder organisations]	Managers [Senior management (Executives)]	Perceptions of managers on manager's practices in quality and safety	Executives developed and tested a set of governance best practices in patient safety, such as:<br>• Creation of a Board committee with explicit responsibility for patient safety<br>• Development of Board level safety reports, introduction of educational activities for Board members<br>• Participation of Board members in executive walk rounds.<br>• Executives reviewed measures to assess safety (e.g. incident reports, infection rates, pharmacist interventions, readmissions, etc)<br>• Executives endorsed a statement of public commitment to patient safety.<br>• Executives believed their behaviours affected their organisations' patient safety mission	14/20 (70%)	NA

Table 1 Table of characteristics and summary findings of included studies

For peer review only - http://bmjopen.bmj.com/site/about/guidelines.xhtml

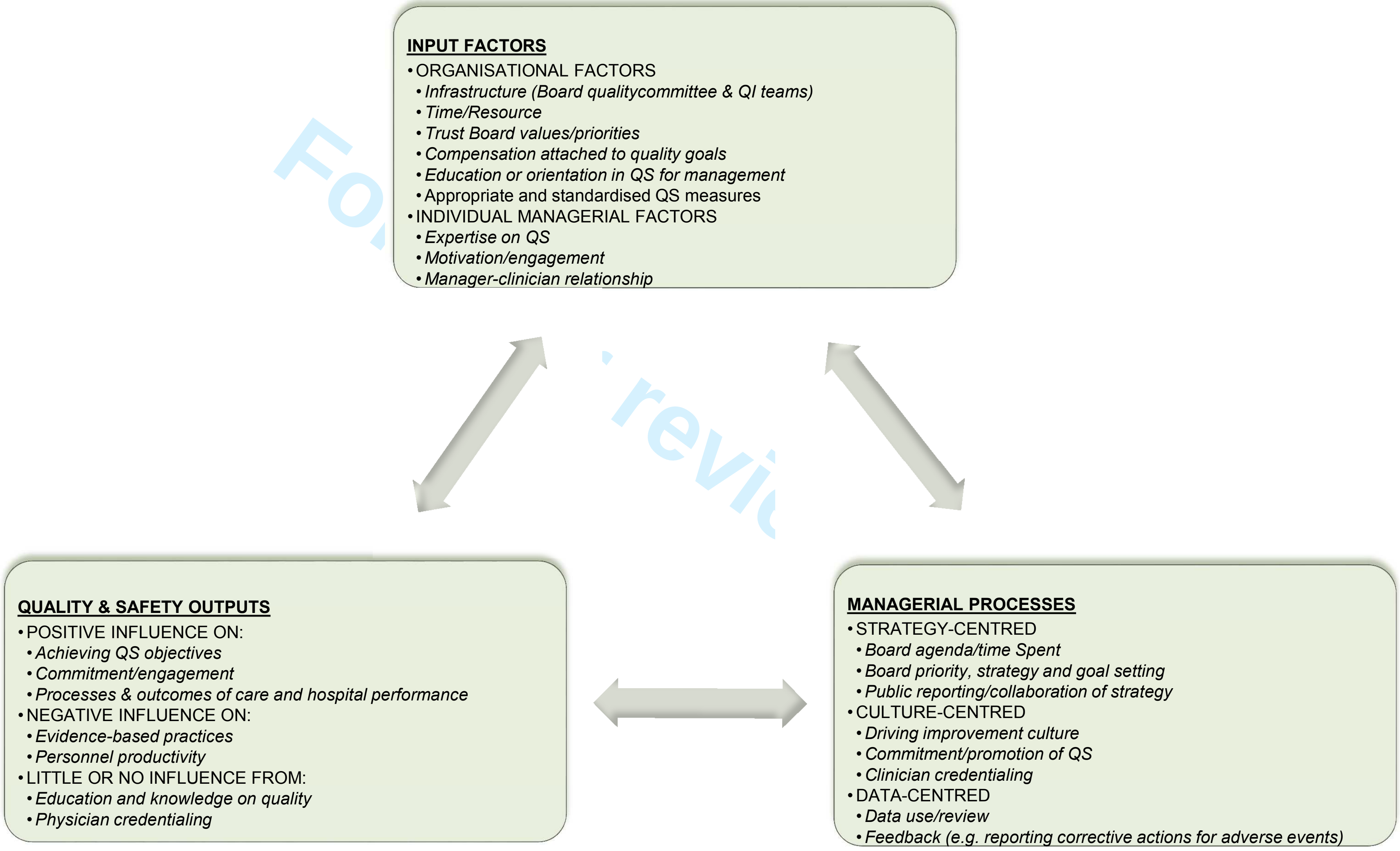

Figure 2 The Quality Management IPO model

For peer review only - http://bmjopen.bmj.com/site/about/guidelines.xhtml

DISCUSSION

Our review examined the role of managers in maintaining and promoting safe, quality care. The existing studies detail the time spent, activities and engagement of hospital managers and Boards, and suggest that these can positively influence quality and safety performance. They further reveal that such involvement is often absent, as are certain conditions that may help them in their work.

Evidence promotes hospitals to have a Board quality committee, with a specific item on quality at the Board meeting, a quality performance measurement report and a dashboard with national quality and safety benchmarks along with standardised quality and safety measures. Outside of the Boardroom, the implications are for senior managers to build a good infrastructure for staff-manager interactions on quality strategies and attach compensation and performance evaluation to quality and safety achievements. For QI programmes, managers should keep in mind its consistency with the hospital's mission and provide commitment, resources, education, and role accountability. Literature elsewhere supports much of these findings, such as the use of quality measurement tools [21, 55] better quality-associated compensation, a separate quality committee,[16, 56] and has also emphasised poor manager-clinician relationships as damaging to patients and QI.[57, 58]

Some of the variables that were shown to be associated with good quality performance, such as having a Board committee, compensation/performance and adoption of system-wide measures, were lacking within the study hospitals. There are also indications of the need to develop Board and senior managerial knowledge and training on quality and safety. Furthermore, this review indicates that many managers do not spend sufficient time on quality and safety. The included studies suggest time spent by the Board should exceed 20-25%, yet the findings expose that certain Boards devote less time than this. Inadequacies of time allocated to quality at the Board meeting hold concerning implications for quality. If little

For peer review only - http://bmjopen.bmj.com/site/about/guidelines.xhtml

time is taken to consider quality of care matters at the highest level, an inference is that less attention will be paid to prevention and improvement of quality within the hospital. While the position that the item appears on the agenda is deemed of high importance, it is unimportant if the duration on this item is overly brief. In this vein, the inadequate time on quality spent by some may reflect their prioritisation on quality in relation to other matters discussed at the meetings or the value perceived to be gained from discussing it further. It might instead however be indicative of the difficulties in measuring time spent on quality by management. Some of these studies provide us not necessarily with Board managers' time on quality and safety but their time spent on this at Board meetings. The two may not equate and time spent on quality may not necessarily be well-spent.[35] The emerging inference that managers greatly prioritise other work over quality and safety is not explicit, with further research required to identify what time is actually devoted and required from managers inside and outside of the Boardroom. Perhaps encouragingly, the more recent studies present more time spent on quality and safety than the earlier studies. Yet even the most recent studies conclude that much improvement is required, further corroborated by very recent empirical research.[59]

This review presents a wide-range of managerial activities, such as public reporting of quality strategies and driving an improvement culture. It further highlights the activities that appear to affect quality performance. Priorities for Boards/managers are to engage in quality, establish goals and strategy to improve care, and get involved in setting the quality agenda, support and promote a safety and QI culture, cultivate leaders, manage resisters, plan ahead and procure organisational resources for quality. Again, much of the findings support the assertions made in the non-empirical literature. Above all, involvement through action, engagement and commitment have been suggested to positively affect quality and safety.[60] While researchers have stressed the limited empirical evidence showing conclusive connection between management commitment and quality,[21] some supporting evidence however can be unearthed in research that concentrates on organisational factors

For peer review only

related to changes made to improve quality and safety in healthcare.[61-63] In addition to this evidence, a few studies have specifically investigated the impact that hospital managers have on quality and safety (rather than examination of their role). These studies have shown senior managerial leadership to be associated with a higher degree of QI implementation,[64] promotion of clinical involvement,[65, 66] safety climate attitudes,[67] and increased Board leadership for quality.[56] A clear case for the positive influence of management involvement with quality is emerging both from the findings of our review and related literature.

There is a dearth of empirical research on the role of hospital managers in quality of care and patient safety and quality improvement. Much of the literature reviewed focused more on the contextual issues surrounding managers' roles, rather than actual manager practices. Some of these outlined managerial actions would further benefit from more detail, for example, the literature fails to present changes made based on data-related activities at the Board or senior management level. Supplementary work could also resolve contradictions that were found within the review, clarifying for example the positive impact of managerial expertise versus knowledge on quality and who sets the Board agenda for the discussion on quality. Research on this area is particularly required to examine middle and frontline managers, to take into consideration non-managers' perceptions, and to assess senior managers' time and tasks outside of the Boardroom. Future studies would benefit from better experimental controls, ideally with more than one time point and more reflections on qualitative work.

Review limitations

There are several limitations of the present review pertaining to the search strategy and review process, the limited sample of studies, publication bias, and limitations of the studies themselves. Specifically, the small number of included studies and their varied study aims,

For peer review only

design and population samples make generalisations difficult. Grouped demographics, such as middle management, are justified by the overlap between positions. With more literature on this topic, distinctions could be made between job positions. Furthermore, more research on lower levels of management would have provided a better balanced review of hospital managers work and contributions to quality. Restricting the language of studies to English in the search strategy is likely to have biased the findings and misrepresent which countries conduct studies on this topic. As most of the study findings relied on self-reports, social desirability may have resulted in exaggerated processes and inflated outputs. Although, encouragingly, one of the included studies found that managers that perceived their Boards to be effective in quality oversight were from hospitals that had higher processes-of-care scores and lower risk adjusted mortality. The quality assessment scores should be viewed with caution; such scores are subjective and may not take into consideration factors beyond the quality assessment scale used.

Conclusion

The modest literature that exists suggests that managers' time spent, engagement and work can influence quality and safety clinical outcomes, processes and performance. Managerial activities that affect quality performance are especially highlighted by this review, such as engagement in quality, establishing goals and strategy to improve care, setting the quality agenda, promoting a quality improvement culture, managing resisters, and procurement of organisational resources for quality. Positive actions to consider include the establishment of a Board quality committee with a specific item on quality at the Board meeting, a quality performance measurement report and a dashboard with national quality and safety benchmarks, performance evaluation attached to quality and safety, and an infrastructure for staff-manager interactions on quality strategies. However, the studies additionally show that many of these contextual factors and activities are lacking within the management of quality within the hospital setting. Moreover, there are indications of a need to develop managerial

For peer review only

For peer review only - http://bmjopen.bmj.com/site/about/guidelines.xhtml

knowledge and training on quality and safety and to increase time devoted to quality and safety. The review suggests time spent by the Board should exceed 20-25%, yet the findings expose that certain Boards devote less time than this. Much of the content of the articles focused on these contextual factors rather than information on the managerial role itself; more empirical research is required to elucidate managers' actual activities. Research is additionally required to examine middle and frontline managers, non-manager perceptions, and to assess senior managers' time and tasks outside of the Boardroom.

For peer review only

- There is a dearth of empirical evidence on hospital managerial work and its influence on quality of care.
- There is some evidence that Boards'/managers' time spent, engagement and work can influence quality and safety clinical outcomes, processes and performance.
- Some variables associated with good quality performance were lacking in study hospitals.
- Many Board managers do not spend sufficient time on quality and safety and need to develop their knowledge on quality and safety.
- There is a greater focus on the contextual issues surrounding managers' roles than on examining managerial activities.
- Research is required to examine middle and frontline managers, to take into consideration non-managers' perceptions, and to assess senior managers' time and tasks outside of the Boardroom
- We present a model to summarise the evidence-based promotion of conditions and activities for managers to best affect quality performance.

Box 1 Key messages from systematic review

CONTRIBUTORS

All co-authors contributed to the study design and review of drafts of the article. The first author screened all of the articles for inclusion in this review and appraised the study quality. Dr Anna Renz and Miss Dina Grishin screened a sample of these at title/abstract and full text, and Miss Ana Wheelock scored the quality of a sample of the included articles.

ACKNOWLEDGEMENTS

For peer review only - http://bmjopen.bmj.com/site/about/guidelines.xhtml

We would like to thank Miss Dina Grishin for helping to review the abstracts and Miss Ana Wheelock for helping to assess the quality of the articles.

COMPETING INTERESTS

There are no competing interests.

FUNDING

This work was supported by funding from the Health Foundation and the National Institute for Health Research.

For peer review only

REFERENCES

1. Kizer KW. Patient Safety: A Call to Action: Safety Is a Critical Component of Quality. Med Gen Med 2001;**3**:10.

2. Kizer KW. Establishing health care performance standards in an era of consumerism. JAMA 2001;**286**:1213-7.

3. Berwick D. Will is the way to win the patient safety war. HSJ 2007;**117**:18-9.

4. IOM. Crossing the quality chasm : a new health system for the 21st century. Washington: National Academy Press; 2001.

5. Gosfield AG, Reinertsen JL. The 100,000 lives campaign: crystallizing standards of care for hospitals. Health aff 2005;**24**:1560-70.

6. Denham CR. Is your hospital as safe as your bank?...Time to ask your board. J Patient Saf 2009;**5**:122-6.

7. Francis R. Report of the Mid Staffordshire NHS Foundation Trust Public Inquiry London: The Stationery Office 2013 [accessed on 18.08.13]. Available from: http://www.midstaffspublicinquiry.com/report.

8. National Advisory Group on the Safety of Patients in England. A promise to learn – a commitment to act: Improving the Safety of Patients in England 2013 [accessed 15.08.13]. Available from: https://www.gov.uk/government/uploads/system/uploads/attachment_data/file/226703/Berwick_Report.pdf.

9. O'Dea A, Flin R. Site managers and safety leadership in the offshore oil and gas industry. Safety Sci 2001;**37**:39-57.

10. Zohar D, Luria G. The use of supervisory practices as leverage to improve safety behavior: a cross-level intervention model. Journal of Safety Res 2003;**34**:567-77.

11. Flin R. "Danger-Men at Work": Management Influence on Safety. Hum Factor Ergon Man 2003;**13**:261-8.

For peer review only

12. Guidotti TL. How can managers visibly promote safety and health protection? J Occ Env Med 2010;**52**:1141-2.

13. Conway J. Getting boards on board: engaging governing boards in quality and safety. Jt Comm J Qual Patient Saf 2008;**34**:214-20.

14. Gautam KS. A call for board leadership on quality in hospitals. Qual Manag Health Care 2005;**14**:18-30.

15. Caldwell C, Butler G, Grah J. Breakthrough quality: what the board must do. Trustee 2008;**61(6)**:32-3.

16. Goeschel CA, Wachter RM, Pronovost PJ. Responsibility for quality improvement and patient safety: hospital board and medical staff leadership challenges. Chest 2010;**138**:171-8.

17. McGinn P, Dave R. What is the board's role in quality? Trustee 2007;**60**:40.

18. Øvretveit J. Leading improvement effectively: Review of research 2009 [accessed on 18.08.13]. Available from: http://www.health.org.uk/public/cms/75/76/313/560/Leading%20imrpovement%20effectively.pdf?realName=YUaqJk.pdf.

19. Flin R, Yule S. Leadership for safety: industrial experience. Quality and Safety in Health Care 2004;**13**:45-51.

20. West E. Management matters: the link between hospital organisation and quality of patient care. Qual Saf Health Care 2001;**10**:40-8.

21. Glickman SW, Baggett KA, Krubert CG, Peterson ED, Schulman KA. Promoting quality: the health-care organization from a management perspective Int J Qual Health Care 2007;**19**:341-8.

22. Gaucher E, Kratochwill EW. The leader's role in implementing total quality management. Qual Manag Health Care 1993;**1**:10-18.

23. Thorman KE, Capitulo KL, Dubow J, Hanold K, Noonan M, Wehmeyer J. Perinatal patient safety from the perspective of nurse executives: a round table discussion. JOGNN 2006;**35**:409-16.

For peer review only

24. AHRQ. The Agency for Healthcare Research and Quality Patient Safety Network Glossary 2011 [accessed on 18.08.13]. Available from: http://www.psnet.ahrq.gov/glossary.aspx?indexLetter=P.

25. Merali F. NHS managers' commitment to a socially responsible role: the NHS managers' views of their core values and their public image. Social Responsibility Journal 2005;**1**:38-46.

26. Mintzberg H. The Nature of Managerial Work. New York: Harper & Row; 1973.

27. Tanon AA, Champagne F, Contandriopoulos AP, Pomey MP, Vadeboncoeur A, Nguyen H. Patient safety and systematic reviews: finding papers indexed in MEDLINE, EMBASE and CINAHL. Qual Saf Health Care 2010;**19**:452-61.

28. Cohen J. A coefficient for agreement for nominal scales. Educ Psychol Meas 1960;**20**:37–46.

29. Cicchetti DV, Feinstein AR. High agreement but low kappa: II. Resolving the paradoxes. J clin epidemiol 1990;**43**:551-8.

30. Viera AJ, Garrett JM. Understanding interobserver agreement: the kappa statistic. Family med 2005;**37**:360-3.

31. Baker GR, Denis JL, Pomey MP, MacIntosh-Murray A. Effective governance for quality and patient safety in Canadian Healthcare organizations: a report to the Canadian Health Services Research Foundation and the Canadian Patient Safety Institute. Ottawa: Canadian Health Services Research Foundation and Canadian Patient Safety Institute, 2010.

32. Moher D, Liberati A, Tetzlaff J, Altman DG. Preferred reporting items for systematic reviews and meta-analyses: the PRISMA statement. Ann Intern Med 2009;**151**:264-9.

33. Kmet LM, Lee RC, Cook LS. Standard Quality Assessment Criteria for Evaluating Primary Research Papers from a Variety of Fields. Edmonton: Alberta Heritage Foundation for Medical Research, 2004.

34. Joshi MS, Hines SC. Getting the board on board: Engaging hospital boards in quality and patient safety. Jt Comm J Qual Patient Saf 2006;**32**:179-87.

For peer review only

35. Mastal MF, Joshi M, Schulke K. Nursing leadership: championing quality and patient safety in the boardroom. Nurs Econ 2007;**25**:323-30.

36. Jiang HJ, Lockee C, Bass K, Fraser I. Board engagement in quality: findings of a survey of hospital and system leaders. J Healthc Manag 2008;**53**:121-34.

37. Jiang HJ, Lockee C, Bass K, Fraser I. Board oversight of quality: Any differences in process of care and mortality? J Healthc Manag 2009;**54**:15-29.

38. Walsh D, Downe S. Meta-synthesis method for qualitative research: a literature review. J Adv Nurs 2005;**50**:204-11.

39. Bradley EH, Webster TR, Schlesinger M, Baker D, Inouye SK. The roles of senior management in improving hospital experiences for frail older adults. J Healthc Manag 2006;**51**:323-36.

40. Caine C, Kenrick M. The role of clinical directorate managers in facilitating evidence-based practice: a report of an exploratory study. J Nurs Manag 1997;**5**:157-65.

41. Fox RT, Fox DH, Wells PJ. Performance of first-line management functions on productivity of hospital unit personnel. J Nurs Adm 1999;**29**:12-8.

42. Harris A. Risk management in practice: how are we managing? Clin Perform Qual Health Care 2000;**8**:142-9.

43. Saint S, Kowalski CP, Banaszak-Holl J, Forman J, Damschroder L, Krein SL. The importance of leadership in preventing healthcare-associated infection: Results of a multisite qualitative study. Infection control and hospital epidemiology Infect Control Hosp Epidemiol 2010;**31 (9)**:901-7.

44. Poniatowski L, Stanley S, Youngberg B. Using information to empower nurse managers to become champions for patient safety. Nurs Adm Q 2005;**29**:72-7.

45. Balding C. Embedding organisational quality improvement through middle manager ownership. Int J Health Care Qual Assur 2005;**18**:271-88.

46. Bradley EH, Holmboe ES, Mattera JA, Roumanis SA, Radford MJ, Krumholz HM. The roles of senior management in quality improvement efforts: what are the key components? J Healthc Manag 2003;**48**:15-28.

For peer review only

47. Levey S, Vaughn T, Koepke M, Moore D, Lehrman W, Sinha S. Hospital Leadership and Quality Improvement: Rhetoric Versus Reality. J Patient Saf 2007;**3**:9-15

48. Vaughn T, Koepke M, Kroch E, Lehrman W, Sinha S, Levey S. Engagement of Leadership in Quality Improvement Initiatives: Executive Quality Improvement Survey Results. J Patient Saf 2006;**2**:2-9.

49. Jha A, Epstein A. Hospital governance and the quality of care. Health Aff 2010;**29**:182-7.

50. Prybil LD, Peterson R, Brezinski P, Zamba G, Roach Jr W, Fillmore A. Board oversight of patient care quality in community health systems. Am J Med Qual 2010;**25**:34-41.

51. Braithwaite J, Finnegan TP, Graham EM, Degeling PJ, Hindle D, Westbrook MT. How important are quality and safety for clinician managers? Evidence from triangulated studies. Clin Governance 2004;**9**:34-41.

52. Weingart SN, Page D. Implications for practice: challenges for healthcare leaders in fostering patient safety. Qual Saf Health Care 2004;**13**:52-6.

53. Flin R, Maran N. Identifying and training non-technical skills for teams in acute medicine. Qual Saf Health Care 2004;**13**:80-4.

54. Kunzle B, Kolbe M, Grote G. Ensuring patient safety through effective leadership behaviour: A literature review. Safety Sci 2010;**48**:1-17.

55. Naranjo-Gil D. Strategic performance in hospitals: the use of the balanced scorecard by nurse managers. Health Care Manage Rev 2009;**34**:161-70.

56. Weiner BJ, Alexander JA, Shortell SM. Leadership for quality improvement in health care; empirical evidence on hospital boards, managers, and physicians. Med Care Res Rev 1996;**53**:397-416.

57. Edwards N, Marshall M, McLellan A, Abbasi K. Doctors and managers: A problem without a solution? BMJ 2003;**326**:609-10.

58. Wilkinson JE, Powell A, Davies H. Are clinicians engaged in quality improvement? A review of the literature on healthcare professionals' views on quality improvement initiative:

For peer review only

For peer review only - http://bmjopen.bmj.com/site/about/guidelines.xhtml

Health Foundation Report; 2011 [accessed on 18.08.13]. Available from: http://www.health.org.uk/public/cms/75/76/313/2440/Are%20clinicians%20engaged%20in%20quality%20improvement.pdf?realName=y8NfS4.pdf.

59. Jha AK, Epstein AM. A Survey Of Board Chairs Of English Hospitals Shows Greater Attention To Quality Of Care Than Among Their US Counterparts. Health Aff 2013;**32**:677-85.

60. Stanton P. The role of an NHS Board in assuring the quality of clinically governed care and the duty of trust to patients. Clin Governance 2006;**11**:39-49.

61. Lukas CV, Holmes SK, Cohen AB, Restuccia J, Cramer IE, Shwartz M, et al. Transformational change in health care systems: an organizational model. Health Care Manage Rev 2007;**32**:309-20.

62. Shortell SM, Bennett CL, Byck GR. Assessing the impact of continuous quality improvement on clinical practice: what it will take to accelerate progress. Milbank Q 1998;**76**:593-624.

63. Keroack MA, Youngberg BJ, Cerese JL, Krsek C, Prellwitz LW, Trevelyan EW. Organizational factors associated with high performance in quality and safety in academic medical centers. Acad Med 2007;**82**:1178-86.

64. Parker VA, Wubbenhorst WH, Young GJ, Desai KR, Charns MP. Implementing quality improvement in hospitals: the role of leadership and culture. Am J Med Qual 1999;**14**:64-9.

65. Weiner B, Shortell S, Alexander J. Promoting clinical involvement in hospital quality improvement efforts: the effects on top management, board, and physician leadership. Health serv res 1997;**32**:491-510.

66. Duckers MLA, Stegeman I, Spreeuwenberg P, Wagner C, Sanders K, Groenewegen PP. Consensus on the leadership of hospital CEOs and its impact on the participation of physicians in improvement projects. Health Policy 2009;**91**:306-13.

For peer review only

67. Vogus TJ, Sutcliffe KM. The impact of safety organizing, trusted leadership, and care pathways on reported medication errors in hospital nursing units. Med Care 2007;**45**:997-1002.

For peer review only

APPENDICES TO APPEAR ONLINE

Appendix 1 Systematic review search strategies

Key: */=MeSH term; ti=title; ab=abstract; $=truncation; *=focus of search & substitution; exp=explode; adj=adjacent to*

MEDLINE SEARCH

Step	Search strategy formula
**1**	((safe$.ti,ab. or exp Safety/ or Err$.ti,ab. or Adverse.ti,ab.) and (exp *Risk Management/ or exp medical Errors/ or Safety Management/ or Medical Audit.mp.)) or exp Health Care Quality/ or patient centred care/ or length of stay/ or exp consumer satisfaction/ or patient readmission/ or exp evidence based medicine/ or exp "outcome and process assessment (health care)"/ or exp Quality Indicators, healthcare/ or quality assurance, health care/ or exp clinical audit/ or exp program evaluation/ or guideline adherence/ or (patient centred care or length of stay or customer satisfaction or patient satisfaction or medical audit or clinical audit or clinical effectiveness or performance measurement or outcome assessment or process assessment or guideline adherence or compliance to practice$1 or benchmarking or patient centred care or incident report$ or infection control or patient readmission or evidence based medicine or (evidence based adj2 practice) or waiting times or patient experience or complaints or target$1 or clinical excellence or service excellence or quality).ti,ab.
**2**	exp Hospital/ or (hospital$ or secondary care or acute care or health care organi*ation$1 or healthcare organi*ation$1 or infirmar$).ti,ab.
**3**	exp Health Facility Administrators/ or Governing board/ or (Manager$1 or Matron$1 or CEO$1 or executive$1 or director$3 or board$1 or middle management or senior management or lower management or frontline management or leader$4 or president$1 or head of department$1.ti,ab. or department head$1.ti,ab. or head of nursing or administrator$1 or healthcare administration or (chief adj4 officer$1) or (chief adj4 nurs$) or (chief adj4 operation$1) or (chief adj4 service$1) or chief of staff).ti,ab.
**4**	1 and 2 and 3
**5**	limit 4 to (human and English language and yr="1983-Current")

EMBASE SEARCH

Step	Search strategy formula
**1**	((exp Safety/ or Safe$.ti,ab. or Err$.ti,ab. or Adverse.ti,ab.) and (Health Care Quality.mp. or exp Health Care Organization/ or Health$.ti,ab.)) or exp Health Care Quality/ or clinical effectiveness/ or incident report/ or nursing outcome/ or performance measurement system/ or quality of nursing care/ or length of stay/ or hospital readmission/ or evidence based medicine/ or exp outcome assessment/ or quality control/ or medical audit/ or patient satisfaction/ or (patient centred care or length of stay or customer satisfaction or patient satisfaction or medical audit or clinical audit or clinical effectiveness or performance measurement or outcome assessment or process assessment or guideline adherence or compliance to practice$1 or benchmarking or patient centred care or incident report$ or infection control or patient readmission or evidence based medicine or (evidence based adj2 practice) or waiting times or patient experience or complaints or target$1 or clinical excellence or service excellence or quality).ti,ab.
**2**	exp Hospital/ or Health care organization/ or (hospital$ or secondary care or acute care or health care organi*ation$1 or healthcare organi*ation$1 or infirmar$).ti,ab.
**3**	exp manager/ or (Manager$1 or Matron$1 or CEO$1 or executive$1 or director$3 or board$1 or middle management or senior management or lower management or frontline management or leader$4 or president$1 or head of nursing or head of department$1.ti,ab. or department head$1.ti,ab. or administrator$1 or healthcare administration or health care administration or (chief adj4 officer$1) or (chief adj4 nurs$).ti,ab. or (chief adj4 operation$).ti,ab. or (chief adj4 service$).ti,ab. or chief of staff).ti,ab.
**4**	1 and 2 and 3
**5**	limit 4 to (human and English language and yr=”1983-Current”)

For peer review only - http://bmjopen.bmj.com/site/about/guidelines.xhtml

PSYCHINFO SEARCH

Step	Search strategy formula
**1**	((exp Safety/ or Safe$.ti,ab. or Err$.ti,ab. or Adverse.ti,ab.) and (Health Care Quality.mp. or risk management/ or Health$.ti,ab.)) or exp quality of care/ or quality control/ or Quality of Services/ or Treatment Duration/ or exp consumer satisfaction/ or exp client satisfaction/ or exp evidence based medicine/ or clinical audit/ or (patient centred care or length of stay or customer satisfaction or patient satisfaction or medical audit or clinical audit or clinical effectiveness or performance measurement or outcome assessment or process assessment or guideline adherence or compliance to practice$1 or benchmarking or patient centred care or incident report$ or infection control or patient readmission or evidence based medicine or (evidence based adj2 practice) or waiting times or patient experience or complaints or target$1 or clinical excellence or service excellence or quality).ti,ab.
**2**	Hospitals/ or (hospital$ or secondary care or acute care or health care organi*ation$1 or healthcare organi*ation$1 or infirmar$).ti,ab.
**3**	exp Management Personnel/ or exp Hospital Administration/ or (Manager$1 or Matron$1 or CEO$1 or executive$1 or director$3 or board$1 or middle management or senior management or lower management or frontline management or leader$4 or president$1 or head of department$1.ti,ab. or department head$1.ti,ab. or head of nursing or administrator$1 or healthcare administration or (chief adj4 officer$1) or (chief adj4 nurs$) or (chief adj4 operation$1) or (chief adj4 service$1) or chief of staff).ti,ab.
**4**	1 and 2 and 3
**5**	limit 4 to (human and English language and yr=”1983-Current”)

For peer review only

HMIC SEARCH

Step	Search strategy formula
**1**	((exp Safety/ or Safe$.ti,ab. or Err$.ti,ab. or Adverse.ti,ab.) and (Health Care Quality.mp. or Health$.ti,ab.)) or exp quality assurance in health services/ or patient centred care/ or hospital stay duration/ or exp consumer satisfaction/ or patient readmission/ or exp evidence based medicine/ or exp outcomes/ or benchmarking/ or (patient centred care or length of stay or customer satisfaction or patient satisfaction or medical audit or clinical audit or clinical effectiveness or performance measurement or outcome assessment or process assessment or guideline adherence or compliance to practice$1 or benchmarking or patient centred care or incident report$ or infection control or patient readmission or evidence based medicine or (evidence based adj2 practice) or quality improvement or waiting times or patient experience or complaints or target$1 or clinical excellence or service excellence or quality).ti,ab.
**2**	Acute Hospitals/ or hospital care/ or (hospital$ or secondary care or acute care or health care organi*ation$1 or healthcare organi*ation$1 or infirmar$).ti,ab.
**3**	exp managers/ or "middle and lower management"/ or senior managers/ or top management/ or (Manager$1 or Matron$1 or CEO$1 or executive$1 or director$3 or board$1 or middle management or senior management or lower management or frontline management or leader$4 or president$1 or head of department$1 or department head$1 or head of nursing or administrator$1 or healthcare administration or (chief adj4 officer$1) or (chief adj4 nurs$) or (chief adj4 operation$1) or (chief adj4 service$1) or chief of staff).ti,ab.
**4**	1 and 2 and 3
**5**	limit 4 to (human and English language and yr=”1983-Current”)

For peer review only

Appendix 2 Inclusion/exclusion criteria for systematic review

							Population sample		
	**Type of paper**	**Country**	**Language**	**Study design**	**Aims or focus**	**Setting**	**Population group reported on**	**Types of managers**	**Level of management**
**Inclusion**	Empirical Data	Any	English	Any (e.g. qualitative, quantitative or mixed methods)	To identify, describe or test types of managerial activities (i.e. behaviours, actions, role) relating to Q&S and QSI	Hospital	Hospital Managers	Clinical & operational managers (E.g. general managers, service managers, service delivery managers, directorate managers, Matrons, Ward managers, chief operating officer, chief executive officer or clinical directors or board managers or US administrators) Q&S managers: case managers, quality managers, risk managers, quality improvement managers *(mark as QM)*	Includes any levels of management (e.g. senior or middle management)
**Exclusion**	Commentary/opinion papers; literature reviews; editorials; conference proceedings; books guides; manuals; leaflets; conference proceedings; seminar proceedings; meeting notes; internal letters; Single case studies; and case studies that have no methods description.	None	Non-English	None	Managers' perceptions, knowledge, attitudes, awareness, competencies, leadership styles, or contextual issues (e.g. system barriers to involvement) of Q&S. Quality of cost of care.	Services solely for mental health or solely non-acute care community services; primary care; and all other industries	Managers from other settings; Senior clinicians (e.g. Clinician leaders such as consultants)	Those whose main position is to deal with financial management responsibilities alone<br><br>Other very specialist managers, e.g. materials specialist manager or information manager<br><br>Managers whose official role is quality and safety, e.g. quality manager	None

For peer review only - http://bmjopen.bmj.com/site/about/guidelines.xhtml

Appendix 3 Full review screening for systematic review

Article ID:

Stage 1

**A**	**Aim: Is the aim of the article to investigate work activities/engagement/time?**	**yes**	**no**	**unclear**
**B**	Context: Are the work activities in the context of Quality & Safety?	yes	no	unclear
**C**	Setting: Is the setting related to hospitals)?	yes	no	unclear
**D**	Sample Described: Are the work activities described those of managers?	yes	no	unclear

If no to any of the above then exclude and do not proceed to stage 2.
If yes to all, proceed to Stage 2.

**Proceed to stage 2**	**yes**	**no**

Stage 2

**Type: Is it an empirical study?**	**yes**	**no**	**unclear**

If yes, tick box Yes
If unclear, tick box Maybe
If no tick box Yes commentary

**Yes**	
**Maybe**	
**Yes commentary**	

Terms to identify the inclusion criteria

**A) ROLE**	Definition: Any activities carried out at work
	Key Terms: Role, tasks, work, responsibilities, activities, function, duties, undertaking, briefs, assignments, projects, errands, jobs, etc.
	Not: impact on quality & safety, managers competencies or knowledge on quality & safety etc
**B) QUALITY and/or SAFETY**	Definition: Safe, effective, patient-centred, timely efficient and equitable care.
	Key Terms Quality: Health Care Quality or length of stay or mortality or hospital readmission or exp evidence based medicine or evidence based practice or exp outcome assessment or quality control or medical audit or patient satisfaction or patient centred care or length of stay or mortality or customer satisfaction or patient readmission or evidence based medicine or or waiting times or patient experience or complaints or target(s) or clinical excellence or service excellence or quality or patient safety or medical errors or adverse events
	Not: Cost
**C) HOSPITALS**	Definition: Organisations that provide medical/surgical care
	Key Terms: Hospital(s) or acute care or acute care or health care organisation(s)
	Not: All other non-hospital settings, those that only focus on mental health & non-acute care community services, and all other industries
**D) MANAGEMENT**	Definition: A manager is a person that manages staff and has budgetary and service responsibilities. All levels of management are included (frontline managers, middle managers, senior managers, Board)
	Key Terms: General managers, service managers, service delivery managers, directorate managers, divisional managers, manager(s) or matron(s) or CEO(s) or executive(s) or director(s) or board(s) or middle management or senior management or leader(s) or president(s) or head(s) of nursing or head(s) of department(s) or department head(s) or administrator(s) or healthcare administration or chief officer(s) or chief(s) of staff, etc.
	Not: Clinician leaders such as consultants that do not hold budgetary responsibilities, those with financial management responsibilities, those with an official role in quality & safety (e.g. clinical governance managers, quality managers, risk managers)/

For peer review only - http://bmjopen.bmj.com/site/about/guidelines.xhtml

PRISMA 2009 Checklist

Section/topic	#	Checklist item	Reported on page #
**TITLE**			
Title	1	Identify the report as a systematic review, meta-analysis, or both.	1
**ABSTRACT**			
Structured summary	2	Provide a structured summary including, as applicable: background; objectives; data sources; study eligibility criteria, participants, and interventions; study appraisal and synthesis methods; results; limitations; conclusions and implications of key findings; systematic review registration number.	2
**INTRODUCTION**			
Rationale	3	Describe the rationale for the review in the context of what is already known.	4
Objectives	4	Provide an explicit statement of questions being addressed with reference to participants, interventions, comparisons, outcomes, and study design (PICOS).	NA (not a review of interventions)
**METHODS**			
Protocol and registration	5	Indicate if a review protocol exists, if and where it can be accessed (e.g., Web address), and, if available, provide registration information including registration number.	All protocol info in appendices
Eligibility criteria	6	Specify study characteristics (e.g., PICOS, length of follow-up) and report characteristics (e.g., years considered, language, publication status) used as criteria for eligibility, giving rationale.	7
Information sources	7	Describe all information sources (e.g., databases with dates of coverage, contact with study authors to identify additional studies) in the search and date last searched.	6
Search	8	Present full electronic search strategy for at least one database, including any limits used, such that it could be repeated.	in appendices
Study selection	9	State the process for selecting studies (i.e., screening, eligibility, included in systematic review, and, if applicable, included in the meta-analysis).	7
Data collection process	10	Describe method of data extraction from reports (e.g., piloted forms, independently, in duplicate) and any processes for obtaining and confirming data from investigators.	10
Data items	11	List and define all variables for which data were sought (e.g., PICOS, funding sources) and any assumptions and simplifications made.	6
Risk of bias in individual studies	12	Describe methods used for assessing risk of bias of individual studies (including specification of whether this was done at the study or outcome level), and how this information is to be used in any data synthesis.	10
Summary measures	13	State the principal summary measures (e.g., risk ratio, difference in means).	NA
Synthesis of results	14	Describe the methods of handling data and combining results of studies, if done, including measures of consistency (e.g., I^2) for each meta-analysis.	10

For peer review only

PRISMA 2009 Checklist

Section/topic	#	Checklist item	Reported on page #
Risk of bias across studies	15	Specify any assessment of risk of bias that may affect the cumulative evidence (e.g., publication bias, selective reporting within studies).	10
Additional analyses	16	Describe methods of additional analyses (e.g., sensitivity or subgroup analyses, meta-regression), if done, indicating which were pre-specified.	NA
**RESULTS**			
Study selection	17	Give numbers of studies screened, assessed for eligibility, and included in the review, with reasons for exclusions at each stage, ideally with a flow diagram.	9
Study characteristics	18	For each study, present characteristics for which data were extracted (e.g., study size, PICOS, follow-up period) and provide the citations.	16
Risk of bias within studies	19	Present data on risk of bias of each study and, if available, any outcome level assessment (see item 12).	16
Results of individual studies	20	For all outcomes considered (benefits or harms), present, for each study: (a) simple summary data for each intervention group (b) effect estimates and confidence intervals, ideally with a forest plot.	NA
Synthesis of results	21	Present results of each meta-analysis done, including confidence intervals and measures of consistency.	NA
Risk of bias across studies	22	Present results of any assessment of risk of bias across studies (see Item 15).	11
Additional analysis	23	Give results of additional analyses, if done (e.g., sensitivity or subgroup analyses, meta-regression [see Item 16]).	NA
**DISCUSSION**			
Summary of evidence	24	Summarize the main findings including the strength of evidence for each main outcome; consider their relevance to key groups (e.g., healthcare providers, users, and policy makers).	24
Limitations	25	Discuss limitations at study and outcome level (e.g., risk of bias), and at review-level (e.g., incomplete retrieval of identified research, reporting bias).	26
Conclusions	26	Provide a general interpretation of the results in the context of other evidence, and implications for future research.	27
**FUNDING**			
Funding	27	Describe sources of funding for the systematic review and other support (e.g., supply of data); role of funders for the systematic review.	30

From: Moher D, Liberati A, Tetzlaff J, Altman DG, The PRISMA Group (2009). Preferred Reporting Items for Systematic Reviews and Meta-Analyses: The PRISMA Statement. PLoS Med 6(6): e1000097. doi:10.1371/journal.pmed1000097

For more information, visit: **www.prisma-statement.org**.

For peer review only - http://bmjopen.bmj.com/site/about/guidelines.xhtml

BMJ Open

The Role of Hospital Managers in Quality and Patient Safety. A Systematic Review

Journal:	*BMJ Open*
Manuscript ID:	bmjopen-2014-005055.R1
Article Type:	Research
Date Submitted by the Author:	01-Jul-2014
Complete List of Authors:	Parand, Anam; Imperial College London, Surgery and cancer<br>Dopson, Sue; University of Oxford, Said Business School<br>Renz, Anna; Imperial College London, Surgery and cancer<br>Vincent, Charles; University of Oxford, Experimental Psychology
**Primary Subject Heading**:	Medical management
Secondary Subject Heading:	Health services research
Keywords:	HEALTH SERVICES ADMINISTRATION & MANAGEMENT, Quality in health care < HEALTH SERVICES ADMINISTRATION & MANAGEMENT, Systematic literature review, Patient Safety, Leadership

SCHOLARONE™
Manuscripts

For peer review only

The Role of Hospital Managers in Quality and Patient Safety. A Systematic Review

Anam Parand[1], Sue Dopson[2], Anna Renz[1], Charles Vincent[3]

[1]Department of Surgery & Cancer, Imperial College London, London, UK
[2]Said Business School, University of Oxford, Oxford, UK
[3]Department of Experimental Psychology, University of Oxford, Oxford, UK

Correspondence to: Anam Parand, Department of Surgery & Cancer, Imperial College London, 503, 5th Floor Wright Fleming Building, St Mary's Campus, Norfolk Place, London W2 1PG, UK.
Email: a.parand@imperial.ac.uk; Tel: +44 (0) 207594 9726; Fax: +44 (0)20 7594 9726

Key Words: Quality of health care; Patient safety; Systematic literature review; Leadership, Management.

Abstract Word Count: 236 words

Manuscript Word Count: 5,312 words (excluding title page, abstract, references, acknowledgements and tables).

For peer review only - http://bmjopen.bmj.com/site/about/guidelines.xhtml

ABSTRACT

Objectives: To review the empirical literature to identify the activities, time spent and engagement of hospital managers in quality of care.

Design: A systematic review of the literature.

Methods: A search was carried out on the databases MEDLINE, PSYCHINFO, EMBASE, HMIC. The search strategy covered three facets: management, quality of care, and the hospital setting comprising medical subject headings and key terms. Reviewers screened 15,447 titles/abstracts and 423 full texts were checked against inclusion criteria. Data extraction and quality assessment were performed on 19 included articles.

Results: The majority of studies were set in the US and investigated Board/senior level management. The most common research designs were interviews and surveys on the perceptions of managerial quality and safety practices. Managerial activities comprised strategy, culture and data-centred activities, such as driving improvement culture and promotion of quality, strategy/goal-setting and providing feedback. Significant positive associations with quality included compensation attached to quality, using quality improvement measures and having a Board quality committee. However there is an inconsistency and inadequate employment of these conditions and actions across the sample hospitals.

Conclusion: There is some evidence that managers' time spent and work can influence quality and safety clinical outcomes, processes and performance. However, there is a dearth of empirical studies, further weakened by a lack of objective outcome measures and little examination of actual actions undertaken. We present a model to summarise the conditions and activities that affect quality performance.

For peer review only - http://bmjopen.bmj.com/site/about/guidelines.xhtml

ARTICLE SUMMARY

Strengths and limitations of this study

- This is the first systematic review of the literature that has considered the evidence on Boards' and managers' time spent, engagement and work within the context of quality and safety. This review adds to the widely anecdotal and commentary pieces that speculate on what managers should be doing by presenting what they are actually doing.
- The review reveals conditions and actions conducive to good quality management and offers a model to transparently present these to managers considering their own part in quality and safety.
- The search for this review has screened a vast amount of the literature (over 15,000 articles) across a number of databases.
- The small number of included studies and their varied study aims, design and population samples make generalisations difficult. With more literature on this topic, distinctions could be made between job positions.
- The quality assessment scores are subjective and may not take into consideration factors beyond the quality assessment scale used.

For peer review only

For peer review only - http://bmjopen.bmj.com/site/about/guidelines.xhtml

INTRODUCTION

Managers in healthcare have a legal and moral obligation to ensure a high quality of patient care and to strive to improve care. These managers are in a prime position to mandate policy, systems, procedures and organisational climates. Accordingly, many have argued that it is evident that healthcare managers possess an important and obvious role in quality of care and patient safety and that it is one of the highest priorities of healthcare managers.[1-3] In line with this, there have been calls for Boards to take responsibility for quality and safety outcomes.[4, 5] One article warned hospital leaders of the dangers of following in the path of bankers falling into recession, constrained by their lack of risk awareness and reluctance to take responsibility.[6] To add to the momentum are some high profile publicity of hospital management failures affecting quality and safety, eliciting strong instruction for managerial leadership for quality at the national level in some countries.[7, 8]

Beyond healthcare, there is clear evidence of managerial impact on workplace safety.[9-12] Within the literature on healthcare, there are non-empirical articles providing propositions and descriptions on managerial attitudes and efforts to improve safety and quality. This literature, made up of opinion articles, editorials and single participant experiences, present an array of insightful suggestions and recommendations for actions that hospital managers should take to improve the quality of patient care delivery in their organisation.[13-17] However, researchers have indicated that there is a limited evidence-base on this topic.[18-21] Others highlight the literature focus on the difficulties of the managers' role and the negative results of poor leadership on quality improvement (QI) rather than considering actions that managers presently undertake on quality and safety.[22, 23] Consequently, little is known about what healthcare managers are doing in practice to ensure and improve quality of care and patient safety, how much time they spend on this, and what research-based guidance is available for managers in order for them to decide on appropriate areas to become involved. Due perhaps to the broad nature of the topic, scientific studies exploring

For peer review only - http://bmjopen.bmj.com/site/about/guidelines.xhtml

these acts and their impact are likely to be a methodological challenge, although a systematic review of the evidence on this subject is notably absent. This present systematic literature review aims to identify empirical studies pertaining to the role of hospital managers in quality of care and patient safety. We define 'role' to comprise of managerial activities, time spent and active engagement in quality and safety and its improvement. Whilst the primary research question is on the managers' role, we take into consideration the contextual factors surrounding this role and its impact or importance as highlighted by the included studies. Our overarching question is 'What is the role of hospital managers in quality and safety and its improvement?' The specific review research questions are as follows:

- How much time is spent by hospital managers on quality and safety and its improvement?
- What are the managerial activities that relate to quality and safety and its improvement?
- How are managers engaged in quality and safety and its improvement?
- What impact do managers have on quality and safety and its improvement?
- How do contextual factors influence the managers' role and impact on quality and safety and its improvement?

METHODS

Concepts and definitions

Quality of care and patient safety were defined on the basis of widely accepted definitions from the Institute of Medicine (IOM) and the Agency for Healthcare Research and Quality Patient Safety Network (ARQH PSN). IOM define quality in healthcare as possessing the

For peer review only - http://bmjopen.bmj.com/site/about/guidelines.xhtml

following dimensions: safe, effective, patient-centred, timely, efficient and equitable.[4] They define patient safety simply as *“the prevention of harm to patients”,*[24] and AHRQ define it is *“freedom from accidental or preventable injuries produced by medical care.”*[25] Literature was searched for all key terms associated with quality and patient safety to produce an all-encompassing approach. A manager was defined as an employee that has subordinates, oversees staff, is responsible for staff recruitment and training, and holds budgetary accountabilities. Therefore, all levels of managers including Boards of managers were included in this review with the exception of clinical frontline employees, e.g. doctors or nurses, who may have taken on further managerial responsibilities alongside their work but do not have a primary official role as a manager. Those that have specifically taken on a role for quality of care, e.g. the modern matron, were also excluded. Distinction between senior, middle and frontline management were as follows: senior management hold Trust-wide responsibilities;[26] middle managers are in the middle of the organisational hierarchy chart and have one or more managers reporting to them;[27] frontline managers are defined as managers at the first level of the organisational hierarchy chart who have frontline employees reporting to them. Board managers include all members of the Board. Although, there are overlaps between senior managers and Boards (for example Chief Executive Officers (CEOs) may sit on hospital Boards), we aim to present senior and Board level managers separately due to the differences in their responsibilities and position. Only managers that would manage within or govern hospitals were included, with the exclusion of settings that solely served mental health or that comprised solely of non-acute care community services (in order to keep the sample more homogenous). The definition of ‘Role’ focused on actual engagement, time spent and activities that do or did occur rather than those recommended that should or could occur.

For peer review only - http://bmjopen.bmj.com/site/about/guidelines.xhtml

Search Strategy

Literature was reviewed between 01 Jan 1983 and 01 Nov 2010. Eligible articles were those that described or tested managerial roles pertaining to quality and safety in the hospital setting. Part of the search strategy was based on guidance by Tanon et al (2010).[28] EMBASE, MEDLINE, Health Management Information Consortium (HMIC) and PSYCHINFO databases were searched. The search strategy involved three facets (i.Management; ii.Quality; iii.Hospital Setting) and five steps. A facet (i.e. a conceptual grouping of related search terms) for role was not included in the search strategy, as it would have significantly reduced the sensitivity of the search.

Multiple iterations and combinations of all search terms were tested to achieve the best level of specificity and sensitivity. In addition to the key terms, Medical Subject Headings (MeSH®) terms were used, which were 'exploded' to include all MeSH subheadings. All databases required slightly different MeSH terms (named Emtree in Embase), therefore four variations of the search strategies were used (see online Appendix 1 for the search strategies). Additional limits placed on the search strategy restricted study subjects to human and the language to English. The search strategy identified 15,447 articles after duplicates had been removed.

Screening

Three reviewers (AP, AR and DG) independently screened the titles and abstracts of the articles for studies that fit the inclusion criteria. One reviewer (AP) screened all 15,447 articles, while two additional reviewers screened 30% of the total sample retrieved from the search strategy: AR screened 20% and DG screened 10%. On testing inter-rater reliability, Cohen's kappa correlations showed low agreement between AR and AP ($K=0.157$, $P<0.01$) and between DG and AP ($K=0.137$, $P<0.00$).[29] However, there was a high percentage of agreement between raters (95% and 89% respectively), which reveals a good inter-rater

For peer review only - http://bmjopen.bmj.com/site/about/guidelines.xhtml

reliability.[30, 31] Discrepancies were resolved by discussion and consensus. The main inclusion criteria were that: the setting was hospitals; the population sample reported on were managers; the context was quality and safety; the aim was to identify the managerial activities/time/engagement in quality and safety. The full inclusion/exclusion criteria and screening tool can be accessed in the online Appendices 2-3. Figure 1 presents the numbers of articles included and excluded at each stage of the review process.

Four hundred and twenty-three articles remained for full text screening. One reviewer (AP) screened all articles and a second reviewer (AR) reviewed 7% of these. A moderate agreement inter-rater reliability score was calculated ($K=0.615, P<0.001$) with 73% agreement. The primary reoccurring difference in agreement was regarding whether the article pertained to quality of care, owing to the broad nature of the definition. Each article was discussed individually until a consensus was reached on whether to include or exclude. Hand searching and cross-referencing were carried out in case articles were missed by the search strategy or from restriction of databases. One additional article was identified from hand searching,[32] totalling 19 articles included in the systematic review.

Enter Figure 1

For peer review only - http://bmjopen.bmj.com/site/about/guidelines.xhtml

For peer review only

Data extraction & methodological quality

The characteristics and summary findings of the 19 included studies are presented in Table 1. This Table is a simplified version of a standardised template that was used to ensure consistency in data extracted from each article. Each study was assessed using a quality appraisal tool developed by Kmet et al (2004),[34] which comprised of two checklists (qualitative and quantitative). Random included articles (32%) were scored by AW for scoring consistency. All articles were scored on up to 24 questions with a score between 0-2; Box 1 shows an example definition of what constitutes 'Yes' (2), 'Partial' (1) and 'No' (0) rating criteria. The total percentage scores for each study are presented in Table 1. All studies were included regardless of their quality scores. Some cumulative evidence bias may results from two larger datasets split into more than one study each.[35-38] Through a narrative synthesis, we aimed to maintain the original meanings, interpretations and raw data offered by the articles.[39]

Rating	Criteria to verify whether question or objective is sufficiently described
**Yes**	Is easily identified in the introductory section (or first paragraph of methods section). Specifies (where applicable, depending on study design) *all* of the following: purpose, subjects/target population, and the *specific* intervention(s) /association(s)/descriptive parameter(s) under investigation. A study purpose that only becomes apparent after studying other parts of the paper is *not* considered sufficiently described.
**Partial**	Vaguely/incompletely reported (e.g. "describe the effect of" or "examine the role of" or "assess opinion on many issues" or "explore the general attitudes"...); *or* some information has to be gathered from parts of the paper other than the introduction/background/objective section.
**No**	Question or objective is not reported, or is incomprehensible.
**N/A**	Should not be checked for this question

Box 1 Example of a rating criteria for Kmet's quality assessment[34]

For peer review only - http://bmjopen.bmj.com/site/about/guidelines.xhtml

RESULTS

This results section provides an overview description of the reviewed studies and their key findings. The findings are considered under four main headings: managerial time spent on quality and safety; managerial quality and safety activities; managerial impact on quality and safety; and contextual factors related to managers' quality and safety role. The section ends with a proposed model to summarise the review findings.

Description of the studies

From the 19 included studies, the majority were carried out and set in the US (14 studies), and investigated senior management and/or Boards (13 studies). Of these, 3 focused on senior managers alone (e.g. Chief Nursing Officers), 9 concentrated on Board managers and 1 included a mixture of managerial levels. Only 3 investigated middle managers and 3 examined frontline staff (e.g. clinical directorate managers and unit nurse managers). The settings of the study were mostly Trust or hospital-wide; a few articles were set in specific settings or contexts: elderly care,[40] evidence-based medicine,[41] staff productivity,[42] clinical risk management,[43] and hospital acquired infection prevention.[44] Two studies involved specific interventions,[45, 46] and 7 studies concentrated specifically on QI rather than quality and safety oversight or routine.[35, 40, 45-49] There were a mixture of 6 qualitative design (interviews or focus groups); 8 quantitative survey designs; and 5 mix-methods designs. All but one study employed a cross-sectional design[46].[46] The primary outcome measure used in most studies was perceptions of managerial quality and safety practices. All reported participant perceptions and a majority presented self-reports, that is, either a mixture of self and peer reports, or self-reports alone.[41, 43, 45, 46] Several studies asked participants about their own and/or other managers' involvement in regards to their specific quality improvement intervention or quality/safety issue.[40, 41, 44-47] With some variations, the most common research design was to interview or survey senior manager/Board members (particularly Board chairs, presidents and CEOs) perceptions on

For peer review only - http://bmjopen.bmj.com/site/about/guidelines.xhtml

the Board/senior managers' functions, practices, priorities, agenda, time spent, engagement, challenges/issues, drivers and literacy (e.g. familiarity of key reports) on quality and safety.[35-38, 48-51] Five of these studies included objective process/outcome measures, such as adjusted mortality rates.[35, 37, 38, 49, 50] No other studies included clinical outcome measures.

The quality assessment scores ranged between 50-100%; one study scored (what we consider to be) very low (i.e. <55%), eight studies scored highly (i.e. >75%), two other articles scored highly on one out of two of their studies (quantitative/qualitative), and the remaining eight scored a moderate rating in-between. Almost half of the articles did not adequately describe their qualitative studies. Specifically, 8 failed to fully describe their qualitative data collection methods, often not mentioning a standardised topic guide, what questions were asked of participants, or no mention of consent and confidentiality assurances. In 7 studies there was no or vague qualitative data analysis description, including omitting the type of qualitative analysis used. Six of the studies showed no or poor use of verification procedures to establish credibility and 9 reported no or poor reflexivity. Positively, all study designs were evident, the context of studies were clear and the authors showed a connection to a wider body of knowledge.

Similarly to the qualitative studies, 7 quantitative studies did not fully describe, justify or use appropriate analysis methods. However, compared with the qualitative studies, the quantitative studies suffered more from sampling issues. Three studies had particularly small samples (e.g. n=35) and one had an especially low response rate of 15%. Subject characteristics were insufficiently described in 5 studies; in one case the authors did not state the number of hospitals included in data analysis. Several studies had obtained ordinal data but only presented percentages, and only one study appropriately controlled for confounding variables. Across all articles, all but 3 studies reported clear objectives and asserted conclusions clearly supported by the data.

For peer review only - http://bmjopen.bmj.com/site/about/guidelines.xhtml

Managerial time spent on quality and safety

The studies on Board level managers highlight an inadequate prioritisation of quality and patient safety on the Board agenda and subsequent time spent at Board meetings. Not all hospitals consistently have quality on their Board agenda, for example CEOs and chairpersons across 30 organisations reported that approximately a third of all Board meetings had quality on their agenda,[35] and necessary quality items were not consistently and sometimes never addressed.[36] In all studies examining time spent on quality and safety by the Board, less than half of the total time was spent on quality and safety,[32, 37, 38, 48-51] with a majority of Boards spending 25% or less on quality.[32, 38, 45, 49-51] Findings imply that this may be too low to have a positive influence on quality and safety, as higher quality performance was demonstrated by Boards that spent above 20%/25% on quality.[49, 50] Board members recognised that the usual time spent is insufficient.[48] However, few reported financial goals as more important than quality and safety goals,[32] and health system Boards only spent slightly more time on financial issues than quality.[51] Similar issues are noted by studies on frontline managers; specifically that they placed less time and importance on QI,[42] identified as the least discussed topic by clinical managers.[52]

Managerial quality and safety activities

A broad range of quality-related activities were identified to be undertaken by managers. These are presented by the following three groupings: strategy-centred; data-centred and culture-centred.

For peer review only - http://bmjopen.bmj.com/site/about/guidelines.xhtml

Strategy-centred

Board priority-setting and planning strategies aligned with quality and safety goals were identified as Board managerial actions carried out in several studies. High percentages (over 80% in two studies) of Boards had formally established strategic goals for quality with specific targets, and aimed to create a quality plan integral to their broader strategic agenda.[32, 37] Contrary findings however suggest that the Board rarely set the agenda for the discussion on quality,[37] did not provide the ideas for their strategies,[32] and were largely uninvolved in strategic planning for QI.[48] In the latter case, the non-clinical Board managers felt that they held "passive" roles in quality decisions. This is important considering evidence that connects the activity of setting the hospital quality agenda with better performance in process of care and mortality.[38] Additionally, Boards that established goals in four areas of quality and publicly disseminated strategic goals and reported quality information were linked to high hospital performance.[35, 38, 50]

Culture-centred

Activities aimed at enhancing patient safety/QI culture emerged from several studies across organisational tiers.[44, 47, 48, 53] Board and senior management's activities included encouraging an organisational culture of QI on norms regarding interdepartmental/multi-disciplinary collaboration and advocating QI efforts to clinicians and fellow senior managers, providing powerful messages of safety commitment and influencing the organisation's patient safety mission.[47, 53] Managers at differing levels focused on cultivating a culture of clinical excellence and articulating the organisational culture to staff.[44] Factors to motivate/engage middle and senior management in QI included senior management commitment, provision of resources and managerial role accountability.[40, 46] Findings revealed connections between senior management and Board priorities and values with hospital performance and on middle management quality-related activities. Ensuring capacity for high quality standards also appears within the remit of management, as

For peer review only - http://bmjopen.bmj.com/site/about/guidelines.xhtml

physician credentialing was identified as a Board managers' responsibility in more than one study.[38, 48] From this review it is unclear to what degree Board involvement in the credentialing process has a significant impact on quality.[38, 41]

Data-centred

Information on quality and safety are continually supplied to the Board.[51] At all levels of management, activities around quality and safety data or information were recognised in 6 studies.[35, 38, 43, 45, 47, 53] Activities included collecting and collating information,[43] reviewing quality information,[35, 38, 53] using measures such as incident reports and infection rates to forge changes,[53] using patient satisfaction surveys,[35] taking corrective action based on adverse incidents or trends emphasised at Board meetings,[38] and providing feedback.[43, 47] The studies do not specify the changes made based on the data-related activities by senior managers; one study identified that frontline managers predominantly used data from an incident reporting tool to change policy/practice and training/education and communication between care providers.[45] However, overseeing data generally was found to be beneficial, as hospitals that carried out performance monitoring activities had significantly higher scores in process of care and lower mortality rates than hospitals that did not.[38]

Managerial impact on quality and safety outcomes

We have considered the associations found between specific managerial involvement and its affect on quality and safety. Here, we summarise the impact and importance of their general role. Of the articles that looked at either outcomes of management involvement in quality or at its perceived importance, 6 articles suggested that their role was beneficial to quality and safety performance.[32, 35, 38, 40, 49, 53] Senior management support and engagement was identified as one of the primary factors associated with good hospital-wide quality outcomes and QI programme success.[35, 38, 40, 49] Conversely, 6 articles suggest

For peer review only - http://bmjopen.bmj.com/site/about/guidelines.xhtml

that managers' involvement (from the Board, middle and frontline) has little, no or a negative influence on quality and safety.[35, 38, 41, 42, 44, 49] Practices that showed no significant association with quality measures included Board's participation in physician credentialing.[35, 38] Another noted that if other champion leaders are present, management leadership was not deemed necessary.[44] Two articles identified a negative or inhibitory effect on evidence-based practices and staff productivity from frontline and middle managers.[41, 42]

Contextual factors related to managers' quality and safety role

Most of the articles focussed on issues that influenced the managers' role or their impact, as opposed to discussing the role of the managers. These provide an insight in to the types of conditions in which a manager can best undertake their role to affect quality and safety. Unfortunately it appears that many of these conditions are not in place.

Two studies found that a Board quality committee is a positive variable in quality performance, but that fewer than 60% had them.[38, 50] Similarly, compensation and performance evaluation linked to executive quality performance was identified in 4 articles [35, 37, 38, 49] and associated with better quality performance indicators,[38, 49] but quality measures were insufficiently included in CEOs' performance evaluation.[35, 37] The use of the right measures to drive QI was raised in relation to Board managerial engagement in quality [35] and to impact on patient care improvement,[51] yet, almost half of this sample did not formally adopt system-wide measures and standards for quality. To aid them in these tasks, evidence indicates the common use of QI measure tools, such as a dashboard or scorecard, [37, 49, 50] with promising associations between dashboard use and quality outcomes.[38, 50]

For peer review only - http://bmjopen.bmj.com/site/about/guidelines.xhtml

For peer review only

Other factors linked to quality outcomes include management-staff relationship/high interactions between the Board and medical staff when setting quality strategy,[49] and managerial expertise. Although a connection between knowledge with quality outcomes was not found,[38] high performing hospitals have shown higher self-perceived ability to influence care, expertise at the Board and participation in training programs that have a quality component.[50] Disappointingly, there is a low level of CEO knowledge on quality and safety reports,[35] possibly little Boardroom awareness on salient nursing quality issues,[36] and little practice identified to improve quality literacy for the Board.[32, 37] There is however promise for new managers through relevant training at induction and by recruitment of those with relevant expertise.[32]

The Quality Management IPO Model

The input process output (IPO) model is a conceptual framework that helps to structure the review findings in a useful way, please see Figure 2.[54, 55] This literature may be conceptualised by considering what factors contribute (input) to managerial activities (process) that impact on quality and safety (output). The three factors are interrelated and interchangeable, presented by the cyclical interconnecting diagram. This diagram enables a clearer mental picture of what a manager should consider for their role in quality and safety. Specifically, the input factors suggest certain organisational factors that should be put in place alongside individual factors to prepare for such a role (e.g. standardised quality measures, motivation, education and expertise, and a good relationship with clinicians). The processes present the strategy, culture and data-centred areas where managers (according to the literature) are and/or should be involved (e.g. driving improvement culture, goal-setting and providing feedback on corrective actions for adverse events). The outputs identify managerial influences that are positive, negative or have little or no established association with quality performance (e.g. positive outcomes of care, achieving objectives and engaging others in quality of care). This helps to identify areas where it is possible to make an impact through the processes mentioned. With further empirical studies on this topic, this model

For peer review only - http://bmjopen.bmj.com/site/about/guidelines.xhtml

For peer review only

could be strengthened to become a more robust set of evidence-based criteria and outcomes.

For peer review only

For peer review only - http://bmjopen.bmj.com/site/about/guidelines.xhtml

First author; Year [Country]	Methods	Sample size [number of organisations]	Population sample [Level of management reported on (position of managers)]	Outcome Measure	Management Roles (Managerial quality and safety activities, time spent and engagement & key perceived importance and context factors)	Quality assessment score for qualitative studies	Quality assessment score for quantitative studies	Findings pertaining to research questions *[Time spent; Activities, Engagement; Impact (including perceived effectiveness); Contextual factors]*
Baker et al, 2010 [Canada][32]	Mixed methods (interviews, case studies, surveys)	n=15 interviews; n=4 Board case studies; n=79 surveys [79 organisations]	Managers [Board management]	Perceptions of managers on management Board practices in quality and safety	• Less than half (43%) of Boards reported that they addressed quality and patient safety issues in all meetings<br>• One-third of Boards spend 25% of their time or more on quality and patient safety issues<br>• More than 80% of Boards have formally established strategic goals for quality with specific targets, but a majority of Board chairs indicate that their Boards did not provide the ideas for strategic direction or initiatives<br>• Board chairs reported a low participation in education on quality and safety: 43% reported that all the Board members participated, 19% stated that more than half participated and 23% said it was less than a quarter of the Board<br>• Most Board chairs (87%) reported Board member induction training on responsibilities for quality and safety, although almost a third (30%) reported few or no opportunities for education on this, 42% reported some opportunities and 28% reported many)<br>• Approximately half (57%) of the Board chairs acknowledged recruitment of individuals that have knowledge, skills and experience in quality and patient safety onto the Board. A Board skills matrix included quality and safety as one of the competency areas<br>• Over half (55%) of board chairs rated their board's effectiveness in quality and safety oversight as very/extremely effective and 40% as somewhat effective.	16/20 (80%)	12/22 (55%)	Time Activities Impact Context
Balding 2005 [Australia] [46]	Mixed methods (action research, surveys & focus groups)	n=35 [1 hospital]	Managers [Middle management (Nursing managers and allied health managers)]	Self-reported perceptions of managers on their engagement in a QI programme	Five elements deemed essential to middle manager engagement:<br>• (1) Senior management commitment and leadership (e.g. senior management provides strategic direction for QI plan)<br>• (2) Provision of resources and opportunities for QI education and information dissemination (e.g. basic QI skills provided to all staff)<br>• (3) Senior and middle manager role accountability (e.g.: senior managers and middle managers agree QI roles and expectations)<br>• (4) Middle manager involvement in QI planning (e.g. senior and middle managers plan together)<br>• (5) Middle managers own and operate QI program (e.g. ongoing review and evaluation of the progress of the QI program by the middle and senior managers)	14/20 (70%)	15/22 (68%)	Activities Engagement Impact

For peer review only - http://bmjopen.bmj.com/site/about/guidelines.xhtml

Bradely et al, 2003 [US][47]	Qualitative (interviews)	n=45 [8 hospitals]	Clinical staff and senior management [Senior management (unspecified)]	Perceptions of roles and activities that comprise senior management's involvement in quality improvement efforts	Five common roles and activities that captured the variation in management involvement in quality improvement efforts:<br>• (1) Personal engagement of senior managers<br>• (2) Management's relationship with clinical staff<br>• (3) Promotion of an organisational culture of quality improvement<br>• (4) Support of quality improvement with organisational structures<br>• (5) Procurement of organisational resources for quality improvement efforts	19/20 (95%)	NA	Activities Engagement Impact
Bradely et al, 2006 [US][40]	Mixed-methods (surveys & interviews)	n=63 survey respondents [63 hospitals]; n=102 interviewees [13 hospitals]	Managers [Senior management (chief operating officer, vice president, medical director, chief nursing officer, director of volunteers, program director)]	Perceptions of management-related factors around the HELP programme	• Providing resources for needed staffing or staff training<br>• Promoting the program among the governing Board, physicians who were initially less involved, and other administrators<br>• Senior management support reported as the primary enabling factor in the implementation of such programmes (96.6%), along with a lack of support as the primary reason for not implementing the programme (65.0%)<br>• The interviews supported that having an administrative champion was considered essential to their program's success	19/20 (95%)	17/22 (77%)	Activities Engagement Impact Context
Braithwaite et al, 2004 [Australia] [52]	Mixed methods (ethnographic work, observations & focus groups)	n=64 managers in focus groups [1 hospital]; Ethnographic case studies and n=4 observed [2 hospitals]	Managers [Frontline management (Medical managers, nurse managers & allied health managers)]	Observations and self-reported perceptions of clinician-managers' activities	• Quality was the least discussed topic (e.g. Continuous quality improvement)<br>• The most discussed topic was people (e.g. staffing, delegating) and organisational issues, e.g. beds and, equipment	16/20 (80%)	NA	Time

For peer review only - http://bmjopen.bmj.com/site/about/guidelines.xhtml

Caine & Kenwrick, 1997[UK][41]	Qualitative (interviews)	n=10 [2 hospitals]	Managers [Middle management (Clinical directorate managers)]	Self-reported perceptions of managers on the managers' role in facilitating evidence-based practice in their nursing teams	• Managers saw their role in research implementation as a facilitator, ensuring quality and financial objectives and standards were met<br>• Managers perceived their facilitatory behaviours produced a low level of clinical change.<br>• Managers are not actively advocating research-based practice and failing to integrate it into everyday practice. Their behaviour inhibited the development of evidence-based nursing practice<br>• Devolved responsibility of use of research to individual professionals	14/20 (70%)	NA	Activities Impact
Fox, Fox & Wells 1999 [US][42]	Quantitative (surveys & self kept activity logs)	n=16 [1 hospital]	Managers [Frontline management (Nurse administrative managers (NAMs))]	Self-reported perceptions of managers on their activities impacting unit personnel productivity & monitored time/effort allocated to each function & managers' hours worked, patient admissions and length of stay.	• The small amount of total management allocated to QI (2.6%) was the least time spent of all management functions<br>• A negative relationship between time spent in QI activities and unit personnel productivity. An increase (from 2.5% to 5%) in QI time/effort by NAMs would reduce staff productivity significantly by approximately 8%.<br>• The greater the experiences of NAMs as managers, the more time spent on QI. These seasoned NAMs spent more time on monitoring, reporting QI results, and quality improvement teams (statistics nor provided)	NA	13/22 (59%)	Time Activities Impact (Objective outcome measure)
Harris, 2000 [UK][43]	Quantitative (surveys)	n=42 [42 hospitals]	Managers [Middle management (Nurse managers)]	Self-reported perceptions of managers on managers' quality and safety practices	• The majority of managers (91%) who received collated incident information used it to feed back to their own staff. 60% always fed back to staff, 28% sometimes did, 2% never did.<br>• Of the Trusts that had written guidance on types of clinical incident to report, 80% of managers had general guidance and fewer (20%) had written speciality specific guidance.<br>• 76% of managers reported information collation of clinical incidents. Of these, 59% were involved in data collection themselves	NA	13/22 (59%)	Activities

For peer review only - http://bmjopen.bmj.com/site/about/guidelines.xhtml

Jha and Epstein, 2010 [US][50]	Quantitative (surveys)	n=722 [767 hospitals]	Managers [Board]	Perceptions of managers on the role of managers in quality and safety & quality outcome measurement (from Hospital Quality Alliance) i.e. 19 practices for care in 3 clinical conditions	• Two-thirds (63%) of Boards had quality as an agenda item at every meeting<br>• Fewer than half (42%) of the hospitals spent at least 20% of the Board's time on clinical quality<br>• 72% of Boards regularly reviewed a quality dashboard<br>• Most respondents reported that their Boards had established, endorsed, or approved goals in four areas of quality: hospital-acquired infections (82%), medication errors (83%), the HQA/Joint commission core measures (72%), and patient satisfaction (91%)<br>High-performing hospitals were more likely than low-performing hospitals to have:<br>• Board reviews of a quality dashboard regularly (<0.001) & of clinical measures (all <0.05)<br>• Quality performance on the agenda at every Board meeting (0.003)<br>• At least 20% of Board time on clinical quality (0.001)<br>• Has a quality subcommittee (0.001)	NA	22/22 (100%)	Time Activities Impact (Objective outcome measure) Context
Jiang et al, 2008 [US][37]	Quantitative (surveys)	n=562 [387 hospitals]	Managers [Board & Senior management (presidents/ CEOs)]	Perceptions of managers on managers' practices in quality and safety; and outcomes of care (composite scores of risk-adjusted mortality indicators)	• 75% of CEOs reported that most to all of the Board meetings have a specific agenda item devoted to quality. Only 41% indicated that the Boards spend more than 20 percent of its meeting time on the specific item of quality.<br>The following activities were most reported to be performed:<br>• Board establishing strategic goals for QI (81.3%)<br>• Use quality dashboards to track performance (86%)<br>• Follow up corrective actions related to adverse events (83%)<br>The following activities were least reported to be performed:<br>• Board involvement in setting the agenda for the discussion on quality (42.4%)<br>• Inclusion of the quality measures in the CEO's performance evaluation (54.6%)<br>• Improvement of quality literacy of Board members (48.9%)<br>• Board written policy on quality and formally communicated it (30.8%)	NA	20/26 (77%)	Time Activities Impact (Objective outcome measure) Context
Jiang et al, 2009 [US][38]	Quantitative (surveys)	n=490 [490 hospitals]	Managers [Board & Senior management (CEOS & Hospital presidents reports)]	Perceptions of managers on manager's practices in quality and safety; and process of care measures (20 measures in 4 clinical areas); and outcome measures (composite scores of risk-adjusted	Board practices found to be associated with better performance (all P<0.05) in process of care (POC) and adjusted mortality (M) included:<br>• Having a Board quality committee (83.8%POC, 6.2M versus 80.2%POC, 7.9M without a committee)<br>• Establishing strategic goals for quality improvement (82.8%POC, 6.6M versus 80.3%POC, 7.9M)<br>• Being involved in setting the quality agenda for the hospital (83.2%POC, 6.4M versus 80.9%POC, 7.7M)<br>• Including a specific item on quality in Board meetings (83.2%POC, 6.5M versus 78.5%POC, 8.6M)<br>• Using a dashboard with national benchmarks and internal data that includes indicators for clinical quality, patient safety, and patient satisfaction (all above 80%POC & below 6.5M versus all below 80%POC and above 7M)<br>• Linking senior executives' performance evaluation to quality and patient safety indicators (83.1%POC, 6.6M versus 80.4%POC, 7.6M)<br>Practices that did NOT show significant association with the quality measures for process and mortality include:<br>• Reporting to the Board of any corrective action related to adverse events	NA	22/24 (92%)	Activities Impact (Objective outcome measure) Context

For peer review only - http://bmjopen.bmj.com/site/about/guidelines.xhtml

				mortality indicators)	(82.5%POC, 7.0M versus 81.8%POC, 6.6M)<br>• Board's participation in physician credentialing (82.8%POC, 6.9M versus 81.5%POC, 6.9M)<br>• Orientation for new Board members on quality(82.9%POC, 6.8M versus 81.7%POC, 7.0M)<br>• Education of Board members on quality issues (82.8%POC, 7.0M versus 81.9%POC, 6.9M)			
Joshi & Hines, 2006 [US][35]	Mixed-methods (surveys & interviews)	n=37 survey respondents; n=47 interviewees [30 hospitals]	Managers [Board & Senior management (CEOs, Board chairs)]	Perceptions of managers on managers' practices in quality and safety and appropriate care measure (ACM) and risk adjusted mortality.	• Board engagement in quality was reported as satisfactory (7.58 by CEOs and 8.10 by Chairs on a 1-10 scale where 10 indicates greatest satisfaction)<br>• Board engagement was positively associated with perceptions of the rate of progress in improvement (r=.44, p = .05), and marginally associated with ACM scores (r=.41, p=.07)<br>• Approximately one third of Board meetings are devoted to discussing quality issues (reported at 35% by CEOs and 27% by Chairs)<br>• Integrating Quality Planning and Strategic Planning was also rated as satisfactory (7.67 by CEOs and 8.85 by Chairs)<br>• Approximately two-thirds of respondents reported using patient satisfaction surveys (70% and 65% reported by CEOs and Chairs respectively)<br>• Low level of CEO expertise in QI, as reported by themselves (2.70) and by Board Chairs (3.35%) on a scale of 1-10 where 1 is low familiarity and 10 is high familiarity.	12/20 (60%)	16/20 (80%)	Time Activities Engagement Impact (Objective outcome measure) Context
Levey et al, 2007 [US][48]	Qualitative (Interviews)	n=96 [18 hospitals]	Managers [Board & Senior management (Hospital Board members, CEOs, Chief Medical Officers, Chief Quality Officers, medical staff leaders)]	Perceptions of managers on managers' role in quality and safety	• Few CEOs were willing to take the lead for transformation to a "culture of quality"<br>• Board members were largely uninvolved in strategic planning for QI<br>• In terms of the Board's quality functions, respondents largely agreed that physician credentialing was their critical responsibility<br>• Non-physicians reported that they felt relegated to "passive" roles in decisions on quality and seemed reluctant to assume leadership roles in the quality domain.<br>• Board meeting agendas maintained a focus on financial issues, although patient safety/care and QI were gaining prominence<br>• About half of the respondents said that quality was not sufficiently highlighted during meetings. Estimates of time devoted to quality and safety issues at Board meetings were between 10% to 35%	13/20 (65%)	NA	Time Activities Engagement Context
Mastal, Joshi & Shulke 2007 [US][36]	Qualitative (interviews and a focus group)	n=73 interviewees; 1 focus group [63 hospitals]	Managers [Board & Senior management (Board chairs, CEOs, Chief Nurse Officers)]	Perceptions of managers on managers' role in quality and safety	• Two chief nursing officers (CNOs) reported that nursing quality was never addressed at Board meetings.<br>• Few of the CNOs, CEOs, and Board chairs responded that issues are discussed more frequently, such as at every meeting.<br>• Quality and patient safety measures for nurses are not consistently addressed during all hospital Board meetings.<br>• Staffing concerns are the most frequent measure of nursing quality reported at the Board level	12/20 (60%)	NA	Time Context

For peer review only

Poniatowski, Stanley & Youngberg, 2005 [US][45]	Quantitative (surveys)	n=515 [16 academic medical centers]	Managers [Frontline management –unclear whether frontline or middle managers (Unit Nurse managers)]	Self-reported perceptions of managers on their practices with Patient Safety Net (PSN)	• Managers reviewed on average 65% of the PSN events reported<br>As a result of what was learned from PSN data, 162 managers detailed their changes made to:<br>• Policies and practices (59%)<br>• Training, education and communication between care providers (27%)<br>• Purchase of new equipment and supplies (8%)<br>• Staffing (6%)	N/A	10/20 (50%)	Activities
Prybil et al, 2010(US) [51]	Quantitative (surveys)	n=123 [712 hospitals]	Managers [Board & Senior management (CEOs & Boards)]	Perceptions of managers on their role in quality and safety	• Health system Boards spent 23% of their Board meeting time on quality and safety issues. They only spent slightly more on financial issues (25.2%) and strategic planning (27.2%)<br>• Almost all (96%) CEOs said that the Boards regularly received formal written reports on quality targets<br>• 88% of CEOs said that the Boards had assigned quality and safety oversight to a standing Board committee<br>• All but one (98.9%) of the CEOs stated that they have specific performance expectations and criteria related to quality and safety<br>• CEOs reported 59% of the Boards formally adopted system-wide measures and standards for quality	N/A	14/22 (64%)	Time Activities Context
Saint et al, 2010 [US][44]	Qualitative (interviews)	n=86 (interviewees) [14 hospitals]	Senior hospital staff and managers [Mixed levels (Nurse managers, chief physicians, Chairs of medicine, Chief of Staffs, hospital directors, CEOs & clinical non-managerial staff)]	Perceptions of managers on managers' practices in healthcare-associated infection (HAI)	• Although committed leadership by CEOs can be helpful, it was not always necessary, provided that other hospital leaders were committed to infection prevention<br>Behaviours of leaders who successfully implemented/facilitated practices to prevent HAI:<br>• Cultivated a culture of clinical excellence and kept their eye on improving patient care<br>• Developed a vision<br>• Articulated the organisational culture well and conveyed that to staff at all levels.<br>• Focused on overcoming barriers and dealing directly with resistant staff or process issues that impeded prevention of HAI<br>• Cultivated leadership skills and inspired the people they supervised (motivating and energising them to work towards the goal of preventing HAI)<br>• Thought strategically while acting locally; planned ahead and left few things to chance<br>• They did the politicking before issues arose for committee votes<br>• They leveraged personal prestige to move initiatives forward<br>• They worked well across disciplines	16/20 (80%)	NA	Activities Engagement Impact

For peer review only

Vaughn et al, 2006 (US)[49]	Quantitative (surveys)	n=413 [413 hospitals]	Managers [Board & Senior management (chief executives & senior quality executives; Board, executives, clinical leadership)]	Perceptions of managers on managers' role in QI & observed hospital Quality index outcomes (risk-adjusted measures of morbidity, mortality, and medical complications)	• 72% of hospital Boards spent one-quarter of their time or less on quality-of-care issues. About 5% of Boards spent more than half of their time on these issues<br>• A majority of respondents reported great influence from government and regulatory agencies (87%), consumers (72%) and accrediting bodies (74%) on quality priorities. Although 44% of respondents also noted that multiple government and regulatory requirements were unhelpful<br>Better quality index scores (QIS) are associated with hospitals where the Board:<br>• Spends more than 25% of their time on quality issues (QIS 83 – QIS mean 100 across hospitals)<br>• Receives a formal quality performance measurement report (QIS 302)<br>• Bases the senior executives' compensation in part on QI performance (QIS 239)<br>• Engages in a great amount of interaction with the medical staff on quality strategy	NA	21/22 (95%)	Time<br>Activities<br>Engagement<br>Impact (Objective outcome measure)<br>Context
Weingart & Page, 2004 [US][53]	Qualitative (case study documentation analysis and meeting discussions and focus group)	n=30 [10 hospitals and other stakeholder organisations]	Managers [Senior management (Executives)]	Perceptions of managers on manager's practices in quality and safety	Executives developed and tested a set of governance best practices in patient safety, such as:<br>• Creation of a Board committee with explicit responsibility for patient safety<br>• Development of Board level safety reports, introduction of educational activities for Board members<br>• Participation of Board members in executive walk rounds.<br>• Executives reviewed measures to assess safety (e.g. incident reports, infection rates, pharmacist interventions, readmissions, etc)<br>• Executives endorsed a statement of public commitment to patient safety.<br>• Executives believed their behaviours affected their organisations' patient safety mission	14/20 (70%)	NA	Activities<br>Impact

Table 1 Table of characteristics and summary findings of included studies

----

Enter Figure 2

For peer review only - http://bmjopen.bmj.com/site/about/guidelines.xhtml

DISCUSSION

Our review examined the role of managers in maintaining and promoting safe, quality care. The existing studies detail the time spent, activities and engagement of hospital managers and Boards, and suggest that these can positively influence quality and safety performance. They further reveal that such involvement is often absent, as are certain conditions that may help them in their work.

Evidence from the review promotes hospitals to have a Board quality committee, with a specific item on quality at the Board meeting, a quality performance measurement report and a dashboard with national quality and safety benchmarks along with standardised quality and safety measures. Outside of the Boardroom, the implications are for senior managers to build a good infrastructure for staff-manager interactions on quality strategies and attach compensation and performance evaluation to quality and safety achievements. For QI programmes, managers should keep in mind its consistency with the hospital's mission and provide commitment, resources, education, and role accountability. Literature elsewhere supports much of these findings, such as the use of quality measurement tools [21, 56] better quality-associated compensation, a separate quality committee,[16, 57] and has also emphasised poor manager-clinician relationships as damaging to patients and QI.[58, 59]

Some of the variables that were shown to be associated with good quality performance, such as having a Board committee, compensation/performance and adoption of system-wide measures, were lacking within the study hospitals. There are also indications of the need to develop Board and senior managerial knowledge and training on quality and safety. Furthermore, this review indicates that many managers do not spend sufficient time on quality and safety. The included studies suggest time spent by the Board should exceed 20-25%, yet the findings expose that certain Boards devote less time than this. Inadequacies of time allocated to quality at the Board meeting hold concerning implications for quality. If little

For peer review only - http://bmjopen.bmj.com/site/about/guidelines.xhtml

time is taken to consider quality of care matters at the highest level, an inference is that less attention will be paid to prevention and improvement of quality within the hospital. While the position that the item appears on the agenda is deemed of high importance, it is unimportant if the duration on this item is overly brief. In this vein, the inadequate time on quality spent by some may reflect their prioritisation on quality in relation to other matters discussed at the meetings or the value perceived to be gained from discussing it further. It might instead however be indicative of the difficulties in measuring time spent on quality by management. Some of these studies provide us not necessarily with Board managers' time on quality and safety but their time spent on this at Board meetings. The two may not equate and time spent on quality may not necessarily be well-spent.[36] The emerging inference that managers greatly prioritise other work over quality and safety is not explicit, with further research required to identify what time is actually devoted and required from managers inside and outside of the Boardroom. Perhaps encouragingly, the more recent studies present more time spent on quality and safety than the earlier studies. Yet even the most recent empirical studies not included in our review conclude that much improvement is required.[60]

This review presents a wide-range of managerial activities, such as public reporting of quality strategies and driving an improvement culture. It further highlights the activities that appear to affect quality performance. Priorities for Boards/managers are to engage in quality, establish goals and strategy to improve care, and get involved in setting the quality agenda, support and promote a safety and QI culture, cultivate leaders, manage resisters, plan ahead and procure organisational resources for quality. Again, much of the findings support the assertions made in the non-empirical literature. Above all, involvement through action, engagement and commitment have been suggested to positively affect quality and safety.[61] While researchers have stressed the limited empirical evidence showing conclusive connection between management commitment and quality,[21] some supporting evidence however can be unearthed in research that concentrates on organisational factors

For peer review only - http://bmjopen.bmj.com/site/about/guidelines.xhtml

related to changes made to improve quality and safety in healthcare.[62-64] In addition to this evidence, a few studies have specifically investigated the impact that hospital managers have on quality and safety (rather than examination of their role). These studies have shown senior managerial leadership to be associated with a higher degree of QI implementation,[65] promotion of clinical involvement,[66, 67] safety climate attitudes,[68] and increased Board leadership for quality.[57] A clear case for the positive influence of management involvement with quality is emerging both from the findings of our review and related literature.

There is a dearth of empirical research on the role of hospital managers in quality of care and patient safety and quality improvement. This evidence is further weakened by the largely descriptive nature of many of the studies. They most lack theoretical underpinnings and appropriate objective measures. Very few studies reported objective clinical quality outcome measures that better show the influence of managerial actions. Moreover, the content of many of the articles was dominated by the contextual issues surrounding managers' roles, rather than actual manager practices. Some of the outlined managerial actions would further benefit from more detail, for example, the literature fails to present changes made based on data-related activities at the Board or senior management level. Only one study clearly demonstrated that senior management and Board priorities can impact upon middle management quality-related activities and engagement. Considering the likely influence that seniors have on their managers, examination of the interactions between the different roles held (e.g. Boards setting policies on quality and middle managers implementing them) would improve our understanding of how these differences reflect in their time spent and actions undertaken. Supplementary work could also resolve contradictions that were found within the review, clarifying for example the positive impact of managerial expertise versus knowledge on quality and who sets the Board agenda for the discussion on quality. Research on this area is particularly required to examine middle and frontline managers, to take into consideration non-managers' perceptions, and to assess senior managers' time

For peer review only

and tasks outside of the Boardroom. Future studies would benefit from better experimental controls, ideally with more than one time point, verifications and reflections on qualitative work, robust statistical analysis, appropriate study controls, consideration of confounding variables, and transparent reporting of population samples, methodologies, and analyses used.

Review limitations

There are several limitations of the present review pertaining to the search strategy and review process, the limited sample of studies, publication bias, and limitations of the studies themselves. Specifically, the small number of included studies and their varied study aims, design and population samples make generalisations difficult. Grouped demographics, such as middle management, are justified by the overlap between positions. With more literature on this topic, distinctions could be made between job positions. Furthermore, more research on lower levels of management would have provided a better balanced review of hospital managers work and contributions to quality. Restricting the language of studies to English in the search strategy is likely to have biased the findings and misrepresent which countries conduct studies on this topic. There is an over-reliance on perceptions across the studies, which ultimately reduces the validity of the conclusions drawn from their findings. As most of the study findings relied on self-reports, social desirability may have resulted in exaggerated processes and inflated outputs. Although, encouragingly, one of the included studies found that managers that perceived their Boards to be effective in quality oversight were from hospitals that had higher processes-of-care scores and lower risk adjusted mortality. The quality assessment scores should be viewed with caution; such scores are subjective and may not take into consideration factors beyond the quality assessment scale used. Due to the enormity of this review, the publication of this article is some time after the search run date. As there is little evidence published on this topic, we consider this not to greatly impact

For peer review only

on the current relevance of the review, particularly as the literature reviewed spans almost three decades.

Conclusion

The modest literature that exists suggests that managers' time spent, engagement and work can influence quality and safety clinical outcomes, processes and performance. Managerial activities that affect quality performance are especially highlighted by this review, such as establishing goals and strategy to improve care, setting the quality agenda, engaging in quality, promoting a quality improvement culture, managing resisters, and procurement of organisational resources for quality. Positive actions to consider include the establishment of a Board quality committee with a specific item on quality at the Board meeting, a quality performance measurement report and a dashboard with national quality and safety benchmarks, performance evaluation attached to quality and safety, and an infrastructure for staff-manager interactions on quality strategies. However, many of these arrangements were not in place within the study samples. There are also indications of a need for managers to devote more time to quality and safety. More than one study suggest time spent by the Board should exceed 20-25%, yet the findings expose that certain Boards devote less time than this. Much of the content of the articles focused on such contextual factors rather than on the managerial role itself; more empirical research is required to elucidate managers' actual activities. Research is additionally required to examine middle and frontline managers, non-manager perceptions, and to assess senior managers' time and tasks outside of the Boardroom. We present the IPO model to summarise the evidence-based promotion of conditions and activities in order to guide managers on the approaches taken to influence quality performance. More robust empirical research with objective outcome measures could strengthen this guidance.

For peer review only

- There is a dearth of empirical evidence on hospital managerial work and its influence on quality of care.
- There is some evidence that Boards'/managers' time spent, engagement and work can influence quality and safety clinical outcomes, processes and performance.
- Some variables associated with good quality performance were lacking in study hospitals.
- Many Board managers do not spend sufficient time on quality and safety.
- There is a greater focus on the contextual issues surrounding managers' roles than on examining managerial activities.
- Research is required to examine middle and frontline managers, to take into consideration non-managers' perceptions, and to assess senior managers' time and tasks outside of the Boardroom. More robust methodologies with objective outcome measures would strengthen the evidence.
- We present a model to summarise the evidence-based promotion of conditions and activities for managers to best affect quality performance.

Box 2 Key messages from the systematic literature review

CONTRIBUTORS

All co-authors contributed to the study design and review of drafts of the article. The first author screened all of the articles for inclusion in this review and appraised the study quality. Dr Anna Renz and Miss Dina Grishin screened a sample of these at title/abstract and full text, and Miss Ana Wheelock scored the quality of a sample of the included articles.

ACKNOWLEDGEMENTS

We would like to thank Miss Dina Grishin for helping to review the abstracts and Miss Ana Wheelock for helping to assess the quality of the articles.

COMPETING INTERESTS

There are no competing interests.

FUNDING

This work was supported by funding from the Health Foundation and the National Institute for Health Research.

For peer review only

Figure Legends

Figure 1 Review stages based on PRISMA Flow Diagram[33]

Figure 2 The Quality Management IPO model

REFERENCES

1. Kizer KW. Patient Safety: A Call to Action: Safety Is a Critical Component of Quality. Medscape General Medicine 2001;**3:10**.

2. Kizer KW. Establishing health care performance standards in an era of consumerism. Journal of the American Medical Association 2001;**286**:1213-7.

3. Berwick D. Will is the way to win the patient safety war. Health Serv J 2007;**117**:18-9.

4. Institute of Medicine. Crossing the quality chasm: a new health system for the 21st century. Washington: National Academy Press; 2001.

5. Gosfield AG, Reinertsen JL. The 100,000 lives campaign: crystallizing standards of care for hospitals. Health aff 2005;**24**:1560–70.

6. Denham CR. Is your hospital as safe as your bank?...Time to ask your board. J Patient Saf 2009;**5**:122-6.

7. Francis R. Report of the Mid Staffordshire NHS Foundation Trust Public Inquiry London: The Stationery Office 2013 [accessed on 18.03.13]. Available from: http://www.midstaffspublicinquiry.com/report.

8. National Advisory Group on the Safety of Patients in England. A promise to learn – a commitment to act: Improving the Safety of Patients in England 2013 [accessed on 15.08.13]. Available from: https://www.gov.uk/government/uploads/system/uploads/attachment_data/file/226703/Berwick_Report.pdf.

9. O'Dea A, Flin R. Site managers and safety leadership in the offshore oil and gas industry. Safety Sci 2001;**37**:39-57.

10. Zohar D, Luria G. The use of supervisory practices as leverage to improve safety behavior: a cross-level intervention model. Journal of Safety Res 2003;**34**:567-77.

11. Flin R. "Danger-Men at Work": Management Influence on Safety. Hum Factor and Ergon Man 2003;**13**:261-8.

12. Guidotti TL. How can managers visibly promote safety and health protection? J Occ Env Med 2010;**52**:1141-2.

13. Conway J. Getting boards on board: engaging governing boards in quality and safety. Jt Comm J Qual Patient Saf 2008;**34**:214-20.

14. Gautam KS. A call for board leadership on quality in hospitals. Qual Manag Health Care 2005;**14**:18-30.

15. Caldwell C, Butler G, Grah J. Breakthrough quality: what the board must do. Trustee 2008;**61**:32-3, 1.

16. Goeschel CA, Wachter RM, Pronovost PJ. Responsibility for quality improvement and patient safety: hospital board and medical staff leadership challenges. Chest 2010;**138**:171-8.

17. McGinn P, Dave R. What is the board's role in quality? Trustee 2007;**60**:40.

18. Øvretveit J. Leading improvement effectively: Review of research 2009 [accessed on 21.12.12]. Available from: http://www.health.org.uk/public/cms/75/76/313/560/Leading%20imrpovement%20effectively.pdf?realName=YUaqJk.pdf.

19. Flin R, Yule S. Leadership for safety: industrial experience. Qual and Saf Health Care 2004;**13**:45-51.

For peer review only

20. West E. Management matters: the link between hospital organisation and quality of patient care. Qual and Saf Health Care 2001;**10**:40-8.

21. Glickman SW, Baggett KA, Krubert CG, Peterson ED, Schulman KA. Promoting quality: the health-care organization from a management perspective. Int J Qual Health Care 2007;**19**:341-8.

22. Gaucher E, Kratochwill EW. The leader's role in implementing total quality management. Qual Manag Health Care 1993;**1**:10-8.

23. Thorman KE, Capitulo KL, Dubow J, Hanold K, Noonan M, Wehmeyer J. Perinatal patient safety from the perspective of nurse executives: a round table discussion. JOGNN 2006;**35** 409-16.

24. Aspden P, Corrigan JM, Wolcott J, Erickson SM. Patient safety: achieving a new standard of care. Institute of Medicine, editor. Washington: National Academies Press; 2004.

25. AHRQ. AHRQ Patient Safety Network Glossary 2011 [accessed on 19.05.12]. Available from: http://www.psnet.ahrq.gov/glossary.aspx?indexLetter=P.

26. Merali F. NHS managers' commitment to a socially responsible role: the NHS managers' views of their core values and their public image. Social Responsibility Journal 2005;**1**:38-46.

27. Mintzberg H. The Nature of Managerial Work. New York: Harper & Row; 1973.

28. Tanon AA, Champagne F, Contandriopoulos AP, Pomey MP, Vadeboncoeur A, Nguyen H. Patient safety and systematic reviews: finding papers indexed in MEDLINE, EMBASE and CINAHL. Quality saf health care 2010;**19**:452-61.

29. Cohen J. A coefficient for agreement for nominal scales. Educ Psych Meas 1960;**20**:37–46.

30. Cicchetti DV, Feinstein AR. High agreement but low kappa: II. Resolving the paradoxes. J Clin Epidemiol 1990;**43**:551-8.

31. Viera AJ, Garrett JM. Understanding interobserver agreement: the kappa statistic. Family med 2005;**37**:360-3.

32. Baker GR, Denis JL, Pomey MP, MacIntosh-Murray A. Effective governance for quality and patient safety in Canadian Healthcare organizations: a report to the Canadian Health Services Research Foundation and the Canadian Patient Safety Institute. Ottawa: Canadian Health Services Research Foundation and Canadian Patient Safety Institute, 2010.

33. Moher D, Liberati A, Tetzlaff J, Altman DG. Preferred reporting items for systematic reviews and meta-analyses: the PRISMA statement. Ann Intern Med 2009;**151**:264-9.

34. Kmet LM, Lee RC, Cook LS. Standard Quality Assessment Criteria for Evaluating Primary Research Papers from a Variety of Fields. Edmonton: Alberta Heritage Foundation for Medical Research, 2004.

35. Joshi MS, Hines SC. Getting the board on board: Engaging hospital boards in quality and patient safety. Jt Comm J Qual Patient Saf 2006;**32**:179-87.

36. Mastal MF, Joshi M, Schulke K. Nursing leadership: championing quality and patient safety in the boardroom. Nurs Econ 2007;**25**:323-30.

37. Jiang HJ, Lockee C, Bass K, Fraser I. Board engagement in quality: findings of a survey of hospital and system leaders. J Healthc Manag 2008;**53**:121-34.

38. Jiang HJ, Lockee C, Bass K, Fraser I. Board oversight of quality: Any differences in process of care and mortality? J Healthc Manag 2009;**54**:15-29.

39. Walsh D, Downe S. Meta-synthesis method for qualitative research: a literature review. J Adv Nurs 2005;**50**:204-11.

40. Bradley EH, Webster TR, Schlesinger M, Baker D, Inouye SK. The roles of senior management in improving hospital experiences for frail older adults. J Healthc Manag 2006;**51**:323-36.

For peer review

For peer review only - http://bmjopen.bmj.com/site/about/guidelines.xhtml

41. Caine C, Kenrick M. The role of clinical directorate managers in facilitating evidence-based practice: a report of an exploratory study. J Nurs Manag 1997;**5**:157-65.

42. Fox RT, Fox DH, Wells PJ. Performance of first-line management functions on productivity of hospital unit personnel. J Nurs Adm 1999;**29**:12-8.

43. Harris A. Risk management in practice: how are we managing? Clin Perform Qual Health Care 2000;**8**:142-9.

44. Saint S, Kowalski CP, Banaszak-Holl J, Forman J, Damschroder L, Krein SL. The importance of leadership in preventing healthcare-associated infection: Results of a multisite qualitative study. Infect Control Hosp Epidemiol 2010;**31**:901-7.

45. Poniatowski L, Stanley S, Youngberg B. Using information to empower nurse managers to become champions for patient safety. Nurs Adm Q 2005;**29**:72-7.

46. Balding C. Embedding organisational quality improvement through middle manager ownership. Int J Health Care Qual Assur 2005;**18**:271-88.

47. Bradley EH, Holmboe ES, Mattera JA, Roumanis SA, Radford MJ, Krumholz HM. The roles of senior management in quality improvement efforts: what are the key components? J Healthc Manag 2003;**48**:15-28.

48. Levey S, Vaughn T, Koepke M, Moore D, Lehrman W, Sinha S. Hospital Leadership and Quality Improvement: Rhetoric Versus Reality. J Patient Saf 2007;**3**:9-15.

49. Vaughn T, Koepke M, Kroch E, Lehrman W, Sinha S, Levey S. Engagement of Leadership in Quality Improvement Initiatives: Executive Quality Improvement Survey Results. J Patient Saf 2006;**2**:2-9.

50. Jha A, Epstein A. Hospital governance and the quality of care: Health Aff 2010;29:182-187.

51. Prybil LD, Peterson R, Brezinski P, Zamba G, Roach Jr W, Fillmore A. Board oversight of patient care quality in community health systems. Am J Med Qual 2010;**25**:34-41.

52. Braithwaite J, Finnegan TP, Graham EM, Degeling PJ, Hindle D, Westbrook MT. How important are quality and safety for clinician managers? Evidence from triangulated studies. Clin Governance 2004;**9**:34-41.

53. Weingart SN, Page D. Implications for practice: challenges for healthcare leaders in fostering patient safety. Qual Saf Health Care 2004;**13**:52-6.

54. Flin R, Maran N. Identifying and training non-technical skills for teams in acute medicine. Qual Saf Health Care 2004;**13**:80-4.

55. Kunzle B, Kolbe M, Grote G. Ensuring patient safety through effective leadership behaviour: A literature review. Safety Sci 2010;**48**:1-17.

56. Naranjo-Gil D. Strategic performance in hospitals: the use of the balanced scorecard by nurse managers. Health Care Manage Rev 2009;**34**:161-70.

57. Weiner BJ, Alexander JA, Shortell SM. Leadership for quality improvement in health care; empirical evidence on hospital boards, managers, and physicians. Med Care Res Rev 1996;**53**:397-416.

58. Edwards N, Marshall M, McLellan A, Abbasi K. Doctors and managers: A problem without a solution? BMJ 2003;**326**:609-10.

59. Wilkinson JE, Powell A, Davies H. Are clinicians engaged in quality improvement? A review of the literature on healthcare professionals' views on quality improvement initiative: Health Foundation Report; 2011 [accessed on 21.12.12]. Available from: http://www.health.org.uk/public/cms/75/76/313/2440/Are%20clinicians%20engaged%20in%20quality%20improvement.pdf?realName=y8NfS4.pdf.

For peer review only - http://bmjopen.bmj.com/site/about/guidelines.xhtml

60. Jha AK, Epstein AM. A Survey Of Board Chairs Of English Hospitals Shows Greater Attention To Quality Of Care Than Among Their US Counterparts. Health Aff 2013;**32**:677-85.

61. Stanton P. The role of an NHS Board in assuring the quality of clinically governed care and the duty of trust to patients. Clin Governance 2006;**11**:39-49.

62. Lukas CV, Holmes SK, Cohen AB, Restuccia J, Cramer IE, Shwartz M, et al. Transformational change in health care systems: an organizational model. Health Care Manage Rev 2007;**32**:309-20.

63. Shortell SM, Bennett CL, Byck GR. Assessing the impact of continuous quality improvement on clinical practice: what it will take to accelerate progress. The Milbank Q 1998;**76**:593-624, 510.

64. Keroack MA, Youngberg BJ, Cerese JL, Krsek C, Prellwitz LW, Trevelyan EW. Organizational factors associated with high performance in quality and safety in academic medical centers. Acad Med 2007;**82**:1178-86.

65. Parker VA, Wubbenhorst WH, Young GJ, Desai KR, Charns MP. Implementing quality improvement in hospitals: the role of leadership and culture. Am J Medical Qual 1999;**14**:64-9.

66. Weiner B, Shortell S, Alexander J. Promoting clinical involvement in hospital quality improvement efforts: the effects on top management, board, and physician leadership. Health Serv Res 1997;**32**:491-510.

67. Duckers MLA, Stegeman I, Spreeuwenberg P, Wagner C, Sanders K, Groenewegen PP. Consensus on the leadership of hospital CEOs and its impact on the participation of physicians in improvement projects. Health Policy 2009;**91**:306-13.

68. Vogus TJ, Sutcliffe KM. The impact of safety organizing, trusted leadership, and care pathways on reported medication errors in hospital nursing units. Med Care 2007;**45**:997-1002.

For peer review only

The Role of Hospital Managers in Quality and Patient Safety. A Systematic Review

Anam Parand[1], Sue Dopson[2], Anna Renz[1], Charles Vincent[3]

[1]Department of Surgery & Cancer, Imperial College London, London, UK
[2]Said Business School, University of Oxford, Oxford, UK
[3]Department of Experimental Psychology, University of Oxford, Oxford, UK

Correspondence to: Anam Parand, Department of Surgery & Cancer, Imperial College London, 503, 5th Floor Wright Fleming Building, St Mary's Campus, Norfolk Place, London W2 1PG, UK.
Email: a.parand@imperial.ac.uk; Tel: +44 (0) 207594 9726; Fax: +44 (0)20 7594 9726

Key Words: Quality of health care; Patient safety; Systematic literature review; Leadership, Management.

Abstract Word Count: 2~~01~~36 words

Manuscript Word Count: ~~4~~5~~,~~,~~330~~312 words (excluding title page, abstract, references, acknowledgements and tables).

For peer review only

For peer review only - http://bmjopen.bmj.com/site/about/guidelines.xhtml

ABSTRACT

Objectives: To review the empirical literature to identify the activities, time spent and engagement of hospital managers in quality of care.

Design: A systematic review of the literature.

Methods: A search was carried out on the databases MEDLINE, PSYCHINFO, EMBASE, HMIC. The search strategy covered three facets: management, quality of care, and the hospital setting comprising medical subject headings and key terms. Reviewers screened 15,447 titles/abstracts and 423 full texts were checked against inclusion criteria. Data extraction and quality assessment were performed on 19 included articles.

Results: The majority of studies were set in the US and investigated Board/senior level management. The most common research designs were interviews and surveys on the perceptions of managerial quality and safety practices. The evidence indicates that managers are involved in activities that are highly important to the quality and safety of their patients. Activities included strategy-setting and reporting, driving improvement culture and promotion of quality and safety. Significant positive associations with quality included setting the quality agenda and compensation attached to quality. Managerial activities comprised strategy, culture and data-centred activities, such as driving improvement culture and promotion of quality, strategy/goal-setting and providing feedback. Significant positive associations with quality included compensation attached to quality, using quality improvement measures and having a Board quality committee. However there is an inconsistency and -inadequate employment of these conditions and actions* across the sample hospitals.

Conclusion: There is some evidence that managers' time spent and work can influence quality and safety clinical outcomes, processes and performance. However, there is a dearth of empirical studies on their work and its influence, further weakened by a lack of objective

Formatted: Font: +Body (Calibri), Not Italic

For peer review only - http://bmjopen.bmj.com/site/about/guidelines.xhtml

outcome measures and little examination of actual actions undertaken. We present a model to summarise the conditions and activities that affect quality performance.

ARTICLE SUMMARY

Strengths and limitations of this study

- This is the first systematic review of the literature that has considered the evidence on Boards' and managers' time spent, engagement and work within the context of quality and safety. This review adds to the widely anecdotal and commentary pieces that speculate on what managers should be doing by presenting what they are actually doing.
- The review reveals conditions and actions conducive to good quality management and offers a model to transparently present these to managers considering their own part in quality and safety.
- The search for this review has screened a vast amount of the literature (over 15,000 articles) across a number of databases.
- The small number of included studies and their varied study aims, design and population samples make generalisations difficult. With more literature on this topic, distinctions could be made between job positions.
- The quality assessment scores are subjective and may not take into consideration factors beyond the quality assessment scale used.

For peer review only - http://bmjopen.bmj.com/site/about/guidelines.xhtml

INTRODUCTION

Managers in healthcare have a legal and moral obligation to ensure a high quality of patient care and to strive to improve care. These managers are in a prime position to mandate policy, systems, procedures and organisational climates. Accordingly, many have argued that it is evident that healthcare managers possess an important and obvious role in quality of care and patient safety and that it is one of the highest priorities of healthcare managers.[1-3] In line with this, there have been calls for Boards to take responsibility for quality and safety outcomes.[4, 5] One article warned hospital leaders of the dangers of following in the path of bankers falling into recession, constrained by their lack of risk awareness and reluctance to take responsibility.[6] To add to the momentum are some high profile publicity of hospital management failures affecting quality and safety, eliciting strong instruction for managerial leadership for quality at the national level in some countries.[7, 8]

Beyond healthcare, there is clear evidence of managerial impact on workplace safety.[9-12] Within the literature on healthcare, there are non-empirical articles providing propositions and descriptions on managerial attitudes and efforts to improve safety and quality. This literature, made up of opinion articles, editorials and single participant experiences, present an array of insightful suggestions and recommendations for actions that hospital managers should take to improve the quality of patient care delivery in their organisation.[13-17] However, researchers have indicated that there is a limited evidence-base on this topic.[18-21] Others highlight the literature focus on the difficulties of the managers' role and the negative results of poor leadership on quality improvement (QI) rather than considering actions that managers presently undertake on quality and safety.[22, 23] Consequently, little is known about what healthcare managers are doing in practice to ensure and improve quality of care and patient safety, how much time they spend on this, and what research-based guidance is available for managers in order for them to decide on appropriate areas to become involved. Due perhaps to the broad nature of the topic, scientific studies exploring

For peer review only - http://bmjopen.bmj.com/site/about/guidelines.xhtml

these acts and their impact are likely to be a methodological challenge, although a systematic review of the evidence on this subject is notably absent. This present systematic literature review aims to identify empirical studies pertaining to the role of hospital managers in quality of care and patient safety. We define 'role' to comprise of managerial activities, time spent and active engagement in quality and safety and its improvement. Whilst the primary research question is on the managers' role, we take into consideration the contextual factors surrounding this role and its impact or importance as highlighted by the included studies. Our overarching question is 'What is the role of hospital managers in quality and safety and its improvement?' The specific review research questions are as follows:

- How much time is spent by hospital managers on quality and safety and its improvement?
- What are the managerial activities that relate to quality and safety and its improvement?
- How are managers engaged in quality and safety and its improvement?
- What impact do managers have on quality and safety and its improvement?
- How do contextual factors influence the managers' role and impact on quality and safety and its improvement?

METHODS

Concepts and definitions

Quality of care and patient safety were defined on the basis of widely accepted definitions from the Institute of Medicine (IOM) and the Agency for Healthcare Research and Quality Patient Safety Network (ARQH PSN). IOM define quality in healthcare as possessing the

Formatted: Font: Arial

Formatted: Font: Arial

Formatted: Font: Arial

For peer review only - http://bmjopen.bmj.com/site/about/guidelines.xhtml

following dimensions: safe, effective, patient-centred, timely, efficient and equitable.[4] They define patient safety simply as *"the prevention of harm to patients"*,[24] and AHRQ define it is, *"freedom from accidental or preventable injuries produced by medical care."*[25] ~~and~~ ~~l~~Literature was searched for all key terms associated with quality and patient safety to produce an ~~all encompassing~~all-encompassing approach. A manager was defined as an~~y~~ employee that ~~manages~~ has subordinates, oversees staff ~~and~~, ~~is likely to hold~~is ~~managerial~~ responsib~~ilities~~le ~~such as~~for ~~budget responsibilities and~~ staff recruitment and training, and holds budgetary accountabilities. Therefore, all levels of managers including Boards of managers were included in this review with the exception of clinical frontline employees, e.g. doctors or nurses, who may have taken on further managerial responsibilities alongside their work but do not have a primary official role as a manager. Those that have specifically taken on a role for quality of care, e.g. the modern matron, were also excluded. Distinction between senior, middle and frontline management were as follows: senior management hold Trust-wide responsibilities;[26] middle managers are in the middle of the organisational hierarchy chart and have one or more managers reporting to them;[27] frontline managers are defined as managers at the first level of the organisational hierarchy chart who have frontline employees reporting to them. Board managers include all members of the Board. Although, there are overlaps between senior managers and Boards (for example Chief Executive Officers (CEOs) may sit on hospital Boards), we aim to present senior and Board level managers separately due to the differences in their responsibilities and position. Only managers that would manage within or govern hospitals were included, with the exclusion of settings that solely served mental health or that comprised solely of non-acute care community services (in order to keep the sample more homogenous). The definition of 'Role' focused on actual engagement, time spent and activities that do or did occur rather than those recommended that should or could occur.

Formatted: Font: Arial

Formatted: Font: Arial

Formatted: Font: Arial

Formatted: Font: (Default) Arial, Font color: Auto

Formatted: Font: +Body (Calibri)

For peer review only - http://bmjopen.bmj.com/site/about/guidelines.xhtml

Search Strategy

Literature was reviewed between 01 Jan 1983 and 01 Nov 2010. Eligible articles were those that described or tested managerial roles pertaining to quality and safety in the hospital setting. Part of the search strategy was based on guidance by Tanon et al (2010).[28] EMBASE, MEDLINE, Health Management Information Consortium (HMIC) and PSYCHINFO databases were searched. The search strategy involved three facets (i.Management; ii.Quality; iii.Hospital Setting) and five steps. A facet (i.e. a conceptual grouping of related search terms) ~~on~~ for role was not included in the search strategy, as it would have significantly reduced the sensitivity of the search.

Formatted: Font: (Default) Arial

Formatted: Font: (Default) Arial

Multiple iterations and combinations of all search terms were tested to achieve the best level of specificity and sensitivity. In addition to the key terms, Medical Subject Headings (MeSH®) terms were used, which were 'exploded' to include all MeSH subheadings. All databases required slightly different MeSH terms (named Emtree in Embase), therefore four variations of the search strategies were used (see online Appendix 1 for the search strategies). Additional limits placed on the search strategy restricted study subjects to human and the language to English. The search strategy identified 15,447 articles after duplicates had been removed.

Screening

Three reviewers (AP, AR and DG) independently screened the titles and abstracts of the articles for studies that fit the inclusion criteria. One reviewer (AP) screened all 15,447 articles, while two additional reviewers screened 30% of the total sample retrieved from the search strategy: AR screened 20% and DG screened 10%. On testing inter-rater reliability, Cohen's kappa correlations showed low agreement between AR and AP (K=0.157, P<0.01) and between DG and AP (K=0.137, P<0.00).[29] However, there was a high percentage of agreement between raters (95% and 89% respectively), which reveals a good inter-rater

For peer review only - http://bmjopen.bmj.com/site/about/guidelines.xhtml

reliability.[30, 31] Discrepancies were resolved by discussion and consensus. The main inclusion criteria were that: the setting was hospitals; the population sample reported on were managers; the context was quality and safety; the aim was to identify the managerial activities/time/engagement in quality and safety. The full inclusion/exclusion criteria and screening tool can be accessed in the online Appendices 2-3. Figure 1 presents the numbers of articles included and excluded at each stage of the review process.

Four hundred and twenty-three articles remained for full text screening. One reviewer (AP) screened all articles and a second reviewer (AR) reviewed 7% of these. A ~~good~~ moderate agreement inter-rater reliability score was calculated (K=0.615,P<0.001) with 73% agreement. The primary reoccurring difference in agreement was regarding whether the article pertained to quality of care, ~~due~~owing to the broad nature of the definition. Each article was discussed individually until a consensus was reached on whether to include or exclude. Hand searching and cross-referencing were carried out in case articles were missed by the search strategy or from restriction of databases. One additional article was identified from hand searching,[32] totalling 19 articles included in the systematic review.

Formatted: Font:

Formatted: Font: (Default) Arial, Not Italic, Font color: Auto

Formatted: Font: (Default) Arial, Not Italic, Font color: Auto

Formatted: Font: (Default) Arial, Not Italic, Font color: Auto

Formatted: Font: (Default) Arial, Not Italic, Font color: Auto

Formatted: Font: (Default) Arial, Not Italic, Font color: Auto

Formatted: Font:

Enter Figure 1

Data extraction & methodological quality

The characteristics and summary findings of the 19 included studies are presented in Table 1. This Table is a simplified version of a standardised template that was used to ensure consistency in data extracted from each article. Each study was assessed using a quality appraisal tool developed by Kmet et al (2004),[34] which comprised of two checklists (qualitative and quantitative). Random included articles (32%) were scored by AW for

For peer review only

scoring consistency. All articles were scored on up to 24 questions with a score between 0-2~~, ; the total percentage scores are presented in Table 1.~~ Box 1 shows an example definition of what constitutes 'Yes' (2), 'Partial' (1) and 'No' (0) rating criteria. The total percentage scores for each study are presented in Table 1. All studies were included regardless of their quality scores. Some cumulative evidence bias may results from two larger datasets split into more than one study each.[35-38] Through a narrative synthesis, we aimed to maintain the original meanings, interpretations and raw data offered by the articles.[39]

Rating	Criteria to verify whether question or objective is sufficiently described
Yes	Is easily identified in the introductory section (or first paragraph of methods section). Specifies (where applicable, depending on study design) *all* of the following: purpose, subjects/target population, and the *specific* intervention(s) /association(s)/descriptive parameter(s) under investigation. A study purpose that only becomes apparent after studying other parts of the paper is *not* considered sufficiently described.
Partial	Vaguely/incompletely reported (e.g. "describe the effect of" or "examine the role of" or "assess opinion on many issues" or "explore the general attitudes"...); *or* some information has to be gathered from parts of the paper other than the introduction/background/objective section.
No	Question or objective is not reported, or is incomprehensible.
N/A	Should not be checked for this question

Box 1 Example of a rating criteria for Kmet's quality assessment[34]

RESULTS

This results section provides an overview description of the reviewed studies and their key findings. The findings are considered under four main headings: managerial time spent on quality and safety; managerial quality and safety activities; managerial impact on quality and safety; and contextual factors related to managers' quality and safety role. The section ends with a proposed model to summarise the review findings.

Formatted: Font: (Default) Arial
Formatted: Font: (Default) Arial
Formatted: Font: (Default) Arial
Formatted: Font:
Formatted: Font: (Default) Arial
Formatted: Font: (Default) Arial
Formatted: Font:
Formatted: Font: Bold
Formatted: Caption
Formatted Table
Formatted: Font: Arial, 10 pt
Formatted: Centered
Formatted: Font: (Default) Arial, 10 pt
Formatted: Left
Formatted: Font: (Default) Arial, 10 pt, Bold
Formatted: Font: (Default) Arial, 10 pt
Formatted: Left, Indent: Left: 0"
Formatted: Font: (Default) Arial
Formatted: Font: (Default) Arial, 10 pt
Formatted: Left
Formatted: Font: (Default) Arial, 10 pt, Bold
Formatted: Font: (Default) Arial, 10 pt
Formatted: Left, Indent: Left: 0"
Formatted: Font: (Default) Arial
Formatted: Font: (Default) Arial, 10 pt
Formatted: Left
Formatted: Font: (Default) Arial, 10 pt, Bold
Formatted: Font: (Default) Arial, 10 pt
Formatted: Left, Indent: Left: 0"
Formatted: Font: (Default) Arial
Formatted: Font: (Default) Arial, 10 pt
Formatted: Left
Formatted: Font: (Default) Arial, 10 pt, Bold
Formatted: Font: (Default) Arial, 10 pt
Formatted: Left, Indent: Left: 0"
Formatted: Font: (Default) Arial
Formatted: Font: 10 pt, Bold
Formatted: Font: 10 pt
Formatted: Font: 10 pt
Formatted: Font: 10 pt
Formatted: Font: (Default) Arial, 10 pt

For peer review only - http://bmjopen.bmj.com/site/about/guidelines.xhtml

Description of the studies

From the 19 included studies, the majority were carried out and set in the US (14 studies), and investigated senior management and/or Boards (13 studies). Of these, 3 focused on senior managers alone (e.g. Chief Nursing Officers), 9 concentrated on Board managers and 1 included a mixture of managerial levels. Only 3 investigated middle managers (e.g. clinical directorate managers) and 3 examined frontlineexamined frontline staff (e.g. clinical directorate managers and e.g. unit nurse managers). The settings of the study were mostly Trust or hospital-wide; a few articles were set in specific settings or contexts: elderly care,[40] evidence-based medicine,[41] staff productivity,[42] clinical risk management,[43] and hospital acquired infection prevention.[44] Two studies were involved in specific interventions,[45, 46] and 7 studies concentrated specifically on QI rather than quality and safety oversight or routine.[35, 40, 45-49] There were a mixture of 6 qualitative design (interviews or focus groups); 8 quantitative survey designs; and 5 mix-methods designs. The majority of authors opted for self-reports and aAll but one study employed a cross-sectional design.[46].[46] The primary outcome measure used in most studies was perceptions of managerial quality and safety practices. All reported participant perceptions and a majority presented self-reports, that is, either a mixture of self and peer reports, or self-reports alone.[41, 43, 45, 46] Several studies asked participants about their own and/or other managers' involvement in regards to their specific quality improvement intervention or quality/safety issue.[40, 41, 44-47] With some variations, the most common research design was to interview or survey senior manager/Board members (particularly Board chairs, presidents and CEOs) perceptions on the Board/senior managers' functions, practices, priorities, agenda, time spent, engagement, challenges/issues, drivers and literacy (e.g. familiarity of key reports) on quality and safety.[35-38, 48-51] Five of these studies included objective process/outcome measures, such as adjusted mortality rates.[35, 37, 38, 49, 50] No other studies included clinical outcome measures.

For peer review only - http://bmjopen.bmj.com/site/about/guidelines.xhtml

The quality assessment scores ranged between 50-100~~%,~~ %; one study scored (what we consider to be) very low (i.e. <55%), eight studies scored highly (i.e. >75%), two other articles scored highly on one out of two of their studies (quantitative/qualitative), and the remaining eight scored a moderate rating in-between. ~~with little use of verification procedures to establish credibility of interpretation and lack of reflexivity of qualitative accounts. Some quantitative results presented descriptives alone and failed to report variances.~~ Almost half of the articles did not adequately describe their qualitative studies. Specifically, 8 failed to fully describe their qualitative data collection methods, often not mentioning a standardised topic guide, what questions were asked of participants, or no mention of consent and confidentiality assurances. In 7 studies there was no or vague qualitative data analysis description, including omitting the type of qualitative analysis used. Six of the studies showed no or poor use of verification procedures to establish credibility and 9 reported no or poor reflexivity. Positively, all study designs were evident, the context of studies were clear and the authors showed a connection to a wider body of knowledge.

Similarly to the qualitative studies, 7 quantitative studies did not fully describe, justify or use appropriate analysis methods. However, compared with the qualitative studies, the quantitative studies suffered more from sampling issues. Three studies had particularly small samples (e.g. n=35) and one had an especially low response rate of 15%. Subject characteristics were insufficiently described in 5 studies; in one case the authors did not state the number of hospitals included in data analysis. Several studies had obtained ordinal data but only presented percentages, and only one study appropriately controlled for confounding variables. Across all articles, all but 3 studies reported clear objectives and asserted conclusions clearly supported by the data.

Formatted: Font: (Default) Arial, Not Italic

Formatted: Font: (Default) Arial, Not Italic

Formatted: Font color: Red

Formatted: Font: Arial

Formatted: Font: Arial

Formatted: Font: Arial

Formatted: Font: Arial

Formatted: Font: (Default) Arial

Formatted: Font: Arial

Formatted: Font: Arial

Formatted: Font: Arial

Formatted: Font: Arial

Formatted: Font: Arial

Formatted: Font: Arial

For Peer Review Only

Managerial time spent on quality and safety

The studies on Board level managers highlight an inadequate prioritisation of quality and patient safety on the Board agenda and subsequent time spent at Board meetings. Not all hospitals consistently have quality on their Board agenda, for example CEOs and chairpersons across 30 organisations reported that approximately a third of all Board meetings had quality on their agenda,[35] and necessary quality items were not consistently and sometimes never addressed.[36] In all studies examining time spent on quality and safety by the Board, less than half of the total time was spent on quality and safety,[32, 37, 38, 48-51] -with a majority of Boards spending 25% or less on quality.[32, 38, 45, 49-51] Findings imply that this may be too low to have a positive influence on quality and safety, as higher quality performance was demonstrated by Boards that spent above 20%/25% on quality.[49, 50] Board members recognised that the usual time spent is insufficient.[48] However, few reported financial goals as more important than quality and safety goals,[32] and health system Boards only spent slightly more time on financial issues than quality.[51] Similar issues are noted by studies on frontline managers; specifically that they placed less time and importance on QI,[42] identified as the least discussed topic by clinical managers.[52]

Managerial quality and safety activities

A broad range of quality-related activities were identified to be undertaken by managers. These are presented by the following three groupings: strategy-centred; data-centred and culture-centred.

For peer review only - http://bmjopen.bmj.com/site/about/guidelines.xhtml

Strategy-centred

Board priority-setting and planning strategies aligned with quality and safety goals were identified as Board managerial actions carried out in several studies. High percentages (over 80% in two studies) of Boards had formally established strategic goals for quality with specific targets, and aimed to create a quality plan integral to their broader strategic agenda.[32, 37] Contrary findings however suggest that the Board rarely set the agenda for the discussion on quality,[37] did not provide the ideas for their strategies,[32] and were largely uninvolved in strategic planning for QI.[48] In the latter case, the non-clinical Board managers felt that they held "passive" roles in quality decisions. This is important considering evidence that connects the activity of setting the hospital quality agenda with better performance in process of care and mortality.[38] Additionally, Boards that established goals in four areas of quality and publicly disseminated strategic goals and reported quality information were linked to high hospital performance.[35, 38, 50]

Culture-centred

Activities aimed at enhancing patient safety/QI culture emerged from several studies across organisational tiers.[44, 47, 48, 53] Board and senior management's activities included encouraging an organisational culture of QI on norms regarding interdepartmental/multi-disciplinary collaboration and advocating QI efforts to clinicians and fellow senior managers, providing powerful messages of safety commitment and influencing the organisation's patient safety mission.[47, 53] Managers at differing levels focused on cultivating a culture of clinical excellence and articulating the organisational culture to staff.[44] Factors to motivate/engage middle and senior management in QI included senior management commitment, provision of resources and managerial role accountability.[40, 46] Findings revealed connections between senior management and Board priorities and values with hospital performance and on middle management quality-related activities. Ensuring capacity for high quality standards also appears within the remit of management, as

For peer review only - http://bmjopen.bmj.com/site/about/guidelines.xhtml

physician credentialing was identified as a Board managers' responsibility in more than one study.[38, 48] From this review it is unclear to what degree Board involvement in the credentialing process has a significant impact on quality.[38, 41]

Data-centred

Information on quality and safety are continually supplied to the Board.[51] At all levels of management, activities around quality and safety data or information were recognised in 6 studies.[35, 38, 43, 45, 47, 53] Activities included collecting and collating information,[43] reviewing quality information,[35, 38, 53] using measures such as incident reports and infection rates to forge changes,[53] using patient satisfaction surveys,[35] taking corrective action based on adverse incidents or trends emphasised at Board meetings,[38] and providing feedback.[43, 47] The studies do not specify the changes made based on the data-related activities by senior managers; one study identified that frontline managers predominantly used data from an incident reporting tool to change policy/practice and training/education and communication between care providers.[45] However, overseeing data generally was found to be beneficial, as hospitals that carried out performance monitoring activities had significantly higher scores in process of care and lower mortality rates than hospitals that did not.[38]

Managerial impact on quality and safety outcomes

We have considered the associations found between specific managerial involvement and its affect on quality and safety. Here, we summarise the impact and importance of their general role. Of the articles that looked at either outcomes of management involvement in quality or at its perceived importance, ~~5~~ 6 articles suggested that their role was beneficial to quality and safety performance.[32, 35, 38, 40, 49, 53] Senior management support and engagement was identified as one of the primary factors associated with good hospital-wide quality outcomes and QI programme success.[35, 38, 40, 49] Conversely, 6 articles suggest

For peer review only - http://bmjopen.bmj.com/site/about/guidelines.xhtml

that managers' involvement (from the Board, middle and frontline) has little, no or a negative influence on quality and safety.[35, 38, 41, 42, 44, 49] Practices that showed no significant association with quality measures included Board's participation in physician credentialing.[35, 38] Another noted that if other champion leaders are present, management leadership was not deemed necessary.[44] Two articles identified a negative or inhibitory effect on evidence-based practices and staff productivity from frontline and middle managers.[41, 42]

Contextual factors related to managers' quality and safety role

Most of the articles focussed on issues that influenced the managers' role or their impact, as opposed to discussing the role of the managers. These provide an insight in to the types of conditions in which a manager can best undertake their role to affect quality and safety. Unfortunately it appears that many of these conditions are not in place.

Two studies found that a Board quality committee is a positive variable in quality performance, but that fewer than 60% had them.[38, 50] Similarly, compensation and performance evaluation linked to executive quality performance was identified in 4 articles [35, 37, 38, 49] and associated with better quality performance indicators,[38, 49] but quality measures were insufficiently included in CEOs' performance evaluation.[35, 37] The use of the right measures to drive QI was raised in relation to Board managerial engagement in quality [35] and to impact on patient care improvement,[51] yet, almost half of this sample did not formally adopt system-wide measures and standards for quality. To aid them in these tasks, evidence indicates the common use of QI measure tools, such as a dashboard or scorecard, [37, 49, 50] with promising associations between dashboard use and quality outcomes.[38, 50]

For peer review only - http://bmjopen.bmj.com/site/about/guidelines.xhtml

Other factors linked to quality outcomes include management-staff relationship/high interactions between the Board and medical staff when setting quality strategy,[49] and managerial expertise. Although a connection between knowledge with quality outcomes was not found,[38] high performing hospitals have shown higher self-perceived ability to influence care, expertise at the Board and participation in training programs that have a quality component.[50] Disappointingly, there is a low level of CEO knowledge on quality and safety reports,[35] possibly little Boardroom awareness on salient nursing quality issues,[36] and little practice identified to improve quality literacy for the Board.[32, 37] There is however promise for new managers through relevant training at induction and by recruitment of those with relevant expertise.[32]

The Quality Management IPO Model

The input process output (IPO) model is a conceptual framework that helps to structure the review findings in a useful way, please see Figure 2.[54, 55] This literature may be conceptualised by considering what factors contribute (input) to managerial activities (process) that impact on quality and safety (output). The three factors are interrelated and interchangeable, presented by the cyclical interconnecting diagram. This diagram enables a clearer mental picture of what a manager should consider for their role in quality and safety. Specifically, the input factors suggest certain organisational factors that should be put in place alongside individual factors to prepare for such a role (e.g. standardised quality measures, motivation, education and expertise, and a good relationship with clinicians). The processes present the strategy, culture and data-centred areas where managers (according to the literature) are and/or should be involved (e.g. driving improvement culture, goal-setting and providing feedback on corrective actions for adverse events). The outputs identify managerial influences that are positive, negative or have little or no established association with quality performance (e.g. positive outcomes of care, achieving objectives and engaging others in quality of care). This helps to identify areas where it is possible to make an impact through the processes mentioned. With further empirical studies on this topic, this model

Formatted: Justified

Formatted: Font: Arial

Formatted: Font: Arial

For peer review only - http://bmjopen.bmj.com/site/about/guidelines.xhtml

could be strengthened to become a more robust set of evidence-based criteria and outcomes.

For peer review only

For peer review only - http://bmjopen.bmj.com/site/about/guidelines.xhtml

First author; Year [Country]	Methods	Sample size [number of organisations]	Population sample [Level of management reported on (position of managers)]	Outcome Measure	Management Roles (Managerial quality and safety activities, time spent and engagement & key perceived importance and context factors)	Quality assessment score for qualitative studies	Quality assessment score for quantitative studies	Findings pertaining to research questions [Time spent; Activities, Engagement; Impact (including perceived effectiveness); Contextual factors]
Baker et al, 2010 [Canada][32]	Mixed methods (interviews, case studies, surveys)	n=15 interviews; n=4 Board case studies; n=79 surveys [79 organisations]	Managers [Board management]	Perceptions of managers on management Board practices in quality and safety	• Less than half (43%) of Boards reported that they addressed quality and patient safety issues in all meetings<br>• One-third of Boards spend 25% of their time or more on quality and patient safety issues.<br>• More than 80% of Boards have formally established strategic goals for quality with specific targets, but a majority of Board chairs indicate that their Boards did not provide the ideas for strategic direction or initiatives.<br>• Board chairs reported a low participation in education on quality and safety: 43% reported that all the Board members participated, 19% stated that more than half participated and 23% said it was less than a quarter of the Board<br>• Most Board chairs (87%) reported Board member induction training on responsibilities for quality and safety, although almost a third (30%) reported few or no opportunities for education on this, 42% reported some opportunities and 28% reported many).<br>• Approximately half (57%) of the Board chairs acknowledged recruitment of individuals that have knowledge, skills and experience in quality and patient safety onto the Board. A Board skills matrix included quality and safety as one of the competency areas<br>• Over half (55%) of board chairs rated their board's effectiveness in quality and safety oversight as very/extremely effective and 40% as somewhat effective.	16/20 (80%)	12/22 (55%)	Time Activities Impact Context
Balding 2005 [Australia] [46]	Mixed methods (action research, surveys & focus groups)	n=35 [1 hospital]	Managers [Middle management (Nursing managers and allied health managers)]	Self-reported ~~Perceptions~~ perceptions of managers on their engagement in a QI programme	Five elements deemed essential to middle manager engagement:<br>• (1) Senior management commitment and leadership (e.g. senior management provides strategic direction for QI plan)<br>• (2) Provision of resources and opportunities for QI education and information dissemination (e.g. basic QI skills provided to all staff)<br>• (3) Senior and middle manager role accountability (e.g.: senior managers and middle managers agree QI roles and expectations)<br>• (4) Middle manager involvement in QI planning (e.g. senior and middle managers plan together)<br>• (5) Middle managers own and operate QI program (e.g. ongoing review and evaluation of the progress of the QI program by the middle and senior managers)	14/20 (70%)	15/22 (68%)	Activities Engagement Impact ~~Context?~~

Formatted Table

For peer review only - http://bmjopen.bmj.com/site/about/guidelines.xhtml

Bradely et al, 2003 [US][47]	Qualitative (interviews)	n=45 [8 hospitals]	Clinical staff and senior management [Senior management (unspecified)]	Perceptions of roles and activities that comprise senior management's involvement in quality improvement efforts	Five common roles and activities that captured the variation in management involvement in quality improvement efforts:<br>• (1) Personal engagement of senior managers<br>• (2) Management's relationship with clinical staff<br>• (3) Promotion of an organisational culture of quality improvement<br>• (4) Support of quality improvement with organisational structures<br>• (5) Procurement of organisational resources for quality improvement efforts	19/20 (95%)	NA	Activities<br>Engagement<br>Impact
Bradely et al, 2006 [US][40]	Mixed-methods (surveys & interviews)	n=63 survey respondents [63 hospitals]; n=102 interviewees [13 hospitals]	Managers [Senior management (chief operating officer, vice president, medical director, chief nursing officer, director of volunteers, program director)]	~~Predominantly self-reported p~~Perceptions of management-related factors around the HELP programme	• Providing resources for needed staffing or staff training<br>• Promoting the program among the governing Board, physicians who were initially less involved, and other administrators<br>• Senior management support reported as the primary enabling factor in the implementation of such programmes (96.6%), along with a lack of support as the primary reason for not implementing the programme (65.0%)<br>• The interviews supported that having an administrative champion was considered essential to their program's success	19/20 (95%)	17/22 (77%)	Activities<br>Engagement<br>Impact<br>Context
Braithwaite et al, 2004 [Australia] [52]	Mixed methods (ethnographic work, observations & focus groups)	n=64 managers in focus groups [1 hospital]; Ethnographic case studies and n=4 observed [2 hospitals]	Managers [Frontline management (Medical managers, nurse managers & allied health managers)]	Observations and self-reported perceptions of clinician-managers' activities	• Quality was the least discussed topic (e.g. Continuous quality improvement)<br>• The most discussed topic was people (e.g. staffing, delegating) and organisational issues, e.g. beds and, equipment	16/20 (80%)	NA	Time

For peer review only

Caine & Kenwrick, 1997[UK][41]	Qualitative (interviews)	n=10 [2 hospitals]	Managers [Middle management (Clinical directorate managers)]	Self-reported pPerceptions of managers on the managers' role in facilitating evidence-based practice in their nursing teams	• Managers saw their role in research implementation as a facilitator, ensuring quality and financial objectives and standards were met<br>• Managers perceived their facilitatory behaviours produced a low level of clinical change.<br>• Managers are not actively advocating research-based practice and failing to integrate it into everyday practice. Their behaviour inhibited the development of evidence-based nursing practice<br>• Devolved responsibility of use of research to individual professionals	14/20 (70%)	NA	Activities Impact
Fox, Fox & Wells 1999 [US][42]	Quantitative (surveys & self kept activity logs)	n=16 [1 hospital]	Managers [Frontline management (Nurse administrative managers (NAMs))]	Self-reported Perceptions perceptions of managers on their activities impacting unit personnel productivity & monitored time/effort allocated to each function & managers' hours worked, patient admissions and length of stay.	• The small amount of total management allocated to QI (2.6%) was the least time spent of all management functions<br>• A negative relationship between time spent in QI activities and unit personnel productivity. An increase (from 2.5% to 5%) in QI time/effort by NAMs would reduce staff productivity significantly by approximately 8%.<br>• The greater the experiences of NAMs as managers, the more time spent on QI. These seasoned NAMs spent more time on monitoring, reporting QI results, and quality improvement teams (statistics nor provided)	NA	13/22 (59%)	Time Activities Impact (Objective outcome measure)
Harris, 2000 [UK][43]	Quantitative (surveys)	n=42 [42 hospitals]	Managers [Middle management (Nurse managers)]	Self-reported pPerceptions of managers on managers' quality and safety practices	• The majority of managers (91%) who received collated incident information used it to feed back to their own staff. 60% always fed back to staff, 28% sometimes did, 2% never did.<br>• Of the Trusts that had written guidance on types of clinical incident to report, 80% of managers had general guidance and fewer (20%) had written speciality specific guidance.<br>• 76% of managers reported information collation of clinical incidents. Of these, 59% were involved in data collection themselves.	NA	13/22 (59%)	Activities

For peer review only

Jha and Epstein, 2010 [US][50]	Quantitative (surveys)	n=722 [767 hospitals]	Managers [Board]	Perceptions of managers on the role of managers in quality and safety & quality outcome measurement (from Hospital Quality Alliance) i.e. 19 practices for care in 3 clinical conditions	• Two-thirds (63%) of Boards had quality as an agenda item at every meeting<br>• Fewer than half (42%) of the hospitals spent at least 20% of the Board's time on clinical quality<br>• 72% of Boards regularly reviewed a quality dashboard<br>• Most respondents reported that their Boards had established, endorsed, or approved goals in four areas of quality: hospital-acquired infections (82%), medication errors (83%), the HQA/Joint commission core measures (72%), and patient satisfaction (91%)<br>High-performing hospitals were more likely than low-performing hospitals to have:<br>• Board reviews of a quality dashboard regularly (<0.001) & of clinical measures (all <0.05)<br>• Quality performance on the agenda at every Board meeting (0.003)<br>• At least 20% of Board time on clinical quality (0.001)<br>• Has a quality subcommittee (0.001)	NA	22/22 (100%)	Time<br>Activities<br>Impact (Objective outcome measure)<br>Context
Jiang et al, 2008 [US][37]	Quantitative (surveys)	n=562 [387 hospitals]	Managers [Board & Senior management (presidents/ CEOs)]	Perceptions of managers on managers' practices in quality and safety; and outcomes of care (composite scores of risk-adjusted mortality indicators)	• 75% of CEOs reported that most to all of the Board meetings have a specific agenda item devoted to quality. Only 41% indicated that the Boards spend more than 20 percent of its meeting time on the specific item of quality.<br>The following activities were most reported to be performed:<br>• Board establishing strategic goals for QI (81.3%)<br>• Use quality dashboards to track performance (86%)<br>• Follow up corrective actions related to adverse events (83%)<br>The following activities were least reported to be performed:<br>• Board involvement in setting the agenda for the discussion on quality (42.4%)<br>• Inclusion of the quality measures in the CEO's performance evaluation (54.6%)<br>• Improvement of quality literacy of Board members (48.9%)<br>• Board written policy on quality and formally communicated it (30.8%)	NA	20/26 (77%)	Time<br>Activities<br>Impact (Objective outcome measure)<br>Context
Jiang et al, 2009 [US][38]	Quantitative (surveys)	n=490 [490 hospitals]	Managers [Board & Senior management (CEOS & Hospital presidents reports)]	Perceptions of managers on manager's practices in quality and safety; and process of care measures (20 measures in 4 clinical areas); and outcome measures (composite scores of risk-adjusted	Board practices found to be associated with better performance (all P<0.05) in process of care (POC) and adjusted mortality (M) included:<br>• Having a Board quality committee (83.8%POC, 6.2M versus 80.2%POC, 7.9M without a committee)<br>• Establishing strategic goals for quality improvement (82.8%POC, 6.6M versus 80.3%POC, 7.9M)<br>• Being involved in setting the quality agenda for the hospital (83.2%POC, 6.4M versus 80.9%POC, 7.7M)<br>• Including a specific item on quality in Board meetings (83.2%POC, 6.5M versus 78.5%POC, 8.6M)<br>• Using a dashboard with national benchmarks and internal data that includes indicators for clinical quality, patient safety, and patient satisfaction (all above 80%POC & below 6.5M versus all below 80%POC and above 7M)<br>• Linking senior executives' performance evaluation to quality and patient safety indicators (83.1%POC, 6.6M versus 80.4%POC, 7.6M)<br>Practices that did NOT show significant association with the quality measures for process and mortality include:<br>• Reporting to the Board of any corrective action related to adverse events	NA	22/24 (92%)	Activities<br>Impact (Objective outcome measure)<br>Context

For peer review only - http://bmjopen.bmj.com/site/about/guidelines.xhtml

				mortality indicators)	(82.5%POC, 7.0M versus 81.8%POC, 6.6M)<br>• Board's participation in physician credentialing (82.8%POC, 6.9M versus 81.5%POC, 6.9M)<br>• Orientation for new Board members on quality(82.9%POC, 6.8M versus 81.7%POC, 7.0M)<br>• Education of Board members on quality issues (82.8%POC, 7.0M versus 81.9%POC, 6.9M)			
Joshi & Hines, 2006 [US][35]	Mixed-methods (surveys & interviews)	n=37 survey respondents; n=47 interviewees [30 hospitals]	Managers [Board & Senior management (CEOs, Board chairs)]	Perceptions of managers on managers' practices in quality and safety and appropriate care measure (ACM) and risk adjusted mortality.	• Board engagement in quality was reported as satisfactory (7.58 by CEOs and 8.10 by Chairs on a 1-10 scale where 10 indicates greatest satisfaction)<br>• Board engagement was positively associated with perceptions of the rate of progress in improvement (r=.44, p = .05), and marginally associated with ACM scores (r=.41, p=.07)<br>• Approximately one third of Board meetings are devoted to discussing quality issues (reported at 35% by CEOs and 27% by Chairs)<br>• Integrating Quality Planning and Strategic Planning was also rated as satisfactory (7.67 by CEOs and 8.85 by Chairs)<br>• Approximately two-thirds of respondents reported using patient satisfaction surveys (70% and 65% reported by CEOs and Chairs respectively)<br>• Low level of CEO expertise in QI, as reported by themselves (2.70) and by Board Chairs (3.35%) on a scale of 1-10 where 1 is low familiarity and 10 is high familiarity.	12/20 (60%)	16/20 (80%)	Time<br>Activities<br>Engagement<br>Impact<br>(Objective outcome measure)<br>Context
Levey et al, 2007 [US][48]	Qualitative (Interviews)	n=96 [18 hospitals]	Managers [Board & Senior management (Hospital Board members, CEOs, Chief Medical Officers, Chief Quality Officers, medical staff leaders)]	Perceptions of managers on managers' role in quality and safety	• Few CEOs were willing to take the lead for transformation to a "culture of quality"<br>• Board members were largely uninvolved in strategic planning for QI<br>• In terms of the Board's quality functions, respondents largely agreed that physician credentialing was their critical responsibility<br>• Non-physicians reported that they felt relegated to "passive" roles in decisions on quality and seemed reluctant to assume leadership roles in the quality domain.<br>• Board meeting agendas maintained a focus on financial issues, although patient safety/care and QI were gaining prominence<br>• About half of the respondents said that quality was not sufficiently highlighted during meetings. Estimates of time devoted to quality and safety issues at Board meetings were between 10% to 35%	13/20 (65%)	NA	Time<br>Activities<br>Engagement<br>Context
Mastal, Joshi & Shulke 2007 [US][36]	Qualitative (interviews and a focus group)	n=73 interviewees; 1 focus group [63 hospitals]	Managers [Board & Senior management (Board chairs, CEOs, Chief Nurse Officers)]	Perceptions of managers on managers' role in quality and safety	• Two chief nursing officers (CNOs) reported that nursing quality was never addressed at Board meetings.<br>• Few of the CNOs, CEOs, and Board chairs responded that issues are discussed more frequently, such as at every meeting.<br>• Quality and patient safety measures for nurses are not consistently addressed during all hospital Board meetings.<br>• Staffing concerns are the most frequent measure of nursing quality reported at the Board level	12/20 (60%)	NA	Time<br>Context

For peer review only - http://bmjopen.bmj.com/site/about/guidelines.xhtml

Poniatowski, Stanley & Youngberg, 2005 [US][45]	Quantitative (surveys)	n=515 [16 academic medical centers]	Managers [Frontline management –unclear whether frontline or middle managers (Unit Nurse managers)]	Self-reported ~~Perceptions~~ perceptions of managers on their practices with Patient Safety Net (PSN)	• Managers reviewed on average 65% of the PSN events reported<br>As a result of what was learned from PSN data, 162 managers detailed their changes made to:<br>• Policies and practices (59%)<br>• Training, education and communication between care providers (27%)<br>• Purchase of new equipment and supplies (8%)<br>• Staffing (6%)	N/A	10/20 (50%)	Activities
Prybil et al, 2010(US) [51]	Quantitative (surveys)	n=123 [712 hospitals]	Managers [Board & Senior management (CEOs & Boards)]	Perceptions of managers on their role in quality and safety	• Health system Boards spent 23% of their Board meeting time on quality and safety issues. They only spent slightly more on financial issues (25.2%) and strategic planning (27.2%)<br>• Almost all (96%) CEOs said that the Boards regularly received formal written reports on quality targets<br>• 88% of CEOs said that the Boards had assigned quality and safety oversight to a standing Board committee<br>• All but one (98.9%) of the CEOs stated that they have specific performance expectations and criteria related to quality and safety<br>• CEOs reported 59% of the Boards formally adopted system-wide measures and standards for quality	N/A	14/22 (64%)	Time Activities Context
Saint et al, 2010 [US][44]	Qualitative (interviews)	n=86 (interviewees) [14 hospitals]	Senior hospital staff and managers [Mixed levels (Nurse managers, chief physicians, Chairs of medicine, Chief of Staffs, hospital directors, CEOs & clinical non-managerial staff)]	Perceptions of managers on managers' practices in healthcare-associated infection (HAI)	• Although committed leadership by CEOs can be helpful, it was not always necessary, provided that other hospital leaders were committed to infection prevention<br>Behaviours of leaders who successfully implemented/facilitated practices to prevent HAI:<br>• Cultivated a culture of clinical excellence and kept their eye on improving patient care<br>• Developed a vision<br>• Articulated the organisational culture well and conveyed that to staff at all levels.<br>• Focused on overcoming barriers and dealing directly with resistant staff or process issues that impeded prevention of HAI<br>• Cultivated leadership skills and inspired the people they supervised (motivating and energising them to work towards the goal of preventing HAI)<br>• Thought strategically while acting locally; planned ahead and left few things to chance<br>• They did the politicking before issues arose for committee votes<br>• They leveraged personal prestige to move initiatives forward<br>• They worked well across disciplines	16/20 (80%)	NA	Activities Engagement Impact

For peer review only

For peer review only - http://bmjopen.bmj.com/site/about/guidelines.xhtml

Vaughn et al, 2006 (US)[49]	Quantitative (surveys)	n=413 [413 hospitals]	Managers [Board & Senior management (chief executives & senior quality executives; Board, executives, clinical leadership)]	Perceptions of managers on managers' role in QI & observed hospital Quality index outcomes (risk-adjusted measures of morbidity, mortality, and medical complications)	• 72% of hospital Boards spent one-quarter of their time or less on quality-of-care issues. About 5% of Boards spent more than half of their time on these issues<br>• A majority of respondents reported great influence from government and regulatory agencies (87%), consumers (72%) and accrediting bodies (74%) on quality priorities. Although 44% of respondents also noted that multiple government and regulatory requirements were unhelpful<br>Better quality index scores (QIS) are associated with hospitals where the Board:<br>• Spends more than 25% of their time on quality issues (QIS 83 – QIS mean 100 across hospitals)<br>• Receives a formal quality performance measurement report (QIS 302)<br>• Bases the senior executives' compensation in part on QI performance (QIS 239)<br>• Engages in a great amount of interaction with the medical staff on quality strategy	NA	21/22 (95%)	Time Activities Engagement Impact (Objective outcome measure) Context
Weingart & Page, 2004 [US][53]	Qualitative (case study documentation analysis and meeting discussions and focus group)	n=30 [10 hospitals and other stakeholder organisations]	Managers [Senior management (Executives)]	Perceptions of managers on manager's practices in quality and safety	Executives developed and tested a set of governance best practices in patient safety, such as:<br>• Creation of a Board committee with explicit responsibility for patient safety<br>• Development of Board level safety reports, introduction of educational activities for Board members<br>• Participation of Board members in executive walk rounds.<br>• Executives reviewed measures to assess safety (e.g. incident reports, infection rates, pharmacist interventions, readmissions, etc)<br>• Executives endorsed a statement of public commitment to patient safety.<br>• Executives believed their behaviours affected their organisations' patient safety mission	14/20 (70%)	NA	Activities Impact

Formatted: Justified

Table 1 Table of characteristics and summary findings of included studies

---

Enter Figure 2

For peer review only - http://bmjopen.bmj.com/site/about/guidelines.xhtml

DISCUSSION

Our review examined the role of managers in maintaining and promoting safe, quality care. The existing studies detail the time spent, activities and engagement of hospital managers and Boards, and suggest that these can positively influence quality and safety performance. They further reveal that such involvement is often absent, as are certain conditions that may help them in their work.

Evidence from the review promotes hospitals to have a Board quality committee, with a specific item on quality at the Board meeting, a quality performance measurement report and a dashboard with national quality and safety benchmarks along with standardised quality and safety measures. Outside of the Boardroom, the implications are for senior managers to build a good infrastructure for staff-manager interactions on quality strategies and attach compensation and performance evaluation to quality and safety achievements. For QI programmes, managers should keep in mind its consistency with the hospital's mission and provide commitment, resources, education, and role accountability. Literature elsewhere supports much of these findings, such as the use of quality measurement tools [21, 56] better quality-associated compensation, a separate quality committee,[16, 57] and has also emphasised poor manager-clinician relationships as damaging to patients and QI.[58, 59]

Some of the variables that were shown to be associated with good quality performance, such as having a Board committee, compensation/performance and adoption of system-wide measures, were lacking within the study hospitals. There are also indications of the need to develop Board and senior managerial knowledge and training on quality and safety. Furthermore, this review indicates that many managers do not spend sufficient time on quality and safety. The included studies suggest time spent by the Board should exceed 20-25%, yet the findings expose that certain Boards devote less time than this. Inadequacies of time allocated to quality at the Board meeting hold concerning implications for quality. If little

For peer review only - http://bmjopen.bmj.com/site/about/guidelines.xhtml

time is taken to consider quality of care matters at the highest level, an inference is that less attention will be paid to prevention and improvement of quality within the hospital. While the position that the item appears on the agenda is deemed of high importance, it is unimportant if the duration on this item is overly brief. In this vein, the inadequate time on quality spent by some may reflect their prioritisation on quality in relation to other matters discussed at the meetings or the value perceived to be gained from discussing it further. It might instead however be indicative of the difficulties in measuring time spent on quality by management. Some of these studies provide us not necessarily with Board managers' time on quality and safety but their time spent on this at Board meetings. The two may not equate and time spent on quality may not necessarily be well-spent.[36] The emerging inference that managers greatly prioritise other work over quality and safety is not explicit, with further research required to identify what time is actually devoted and required from managers inside and outside of the Boardroom. Perhaps encouragingly, the more recent studies present more time spent on quality and safety than the earlier studies. Yet even the most recent empirical studies not included in our review conclude that much improvement is required~~, further corroborated by very recent empirical research~~.[60]

This review presents a wide-range of managerial activities, such as public reporting of quality strategies and driving an improvement culture. It further highlights the activities that appear to affect quality performance. Priorities for Boards/managers are to engage in quality, establish goals and strategy to improve care, and get involved in setting the quality agenda, support and promote a safety and QI culture, cultivate leaders, manage resisters, plan ahead and procure organisational resources for quality. Again, much of the findings support the assertions made in the non-empirical literature. Above all, involvement through action, engagement and commitment have been suggested to positively affect quality and safety.[61] While researchers have stressed the limited empirical evidence showing conclusive connection between management commitment and quality,[21] some supporting evidence however can be unearthed in research that concentrates on organisational factors

For peer review only - http://bmjopen.bmj.com/site/about/guidelines.xhtml

related to changes made to improve quality and safety in healthcare.[62-64] In addition to this evidence, a few studies have specifically investigated the impact that hospital managers have on quality and safety (rather than examination of their role). These studies have shown senior managerial leadership to be associated with a higher degree of QI implementation,[65] promotion of clinical involvement,[66, 67] safety climate attitudes,[68] and increased Board leadership for quality.[57] A clear case for the positive influence of management involvement with quality is emerging both from the findings of our review and related literature.

There is a dearth of empirical research on the role of hospital managers in quality of care and patient safety and quality improvement. This evidence is further weakened by the largely descriptive nature of many of the studies. They most lack theoretical underpinnings and appropriate objective measures. Very few studies reported objective clinical quality outcome measures that better show the influence of managerial actions. Moreover, ~~Much of~~ the ~~literature~~ content of many of the articles was dominated~~reviewed focused more~~ ~~on~~ by the contextual issues surrounding managers' roles, rather than actual manager practices. Some of the~~se~~ outlined managerial actions would further benefit from more detail, for example, the literature fails to present changes made based on data-related activities at the Board or senior management level. Only one study clearly demonstrated that senior management and Board priorities can impact upon middle management quality-related activities and engagement. Considering the likely influence that seniors have on their managers, examination of the interactions between the different roles held (e.g. Boards setting policies on quality and middle managers implementing them) would improve our understanding of how these differences reflect in their time spent and actions undertaken. Supplementary work could also resolve contradictions that were found within the review, clarifying for example the positive impact of managerial expertise versus knowledge on quality and who sets the Board agenda for the discussion on quality.~~-~~ Research on this area is particularly required to examine middle and frontline managers, to take into consideration non-

Formatted: Font: Not Italic

Formatted: Font: Not Italic

Formatted: Font: Not Italic

Formatted: Font: Not Italic

Formatted: Font: Not Italic

Formatted: Font:

Formatted: Font: (Default) Arial

Formatted: Font: (Default) Arial

Formatted: Font:

For peer review only - http://bmjopen.bmj.com/site/about/guidelines.xhtml

managers' perceptions, and to assess senior managers' time and tasks outside of the Boardroom. Future studies would benefit from better experimental controls, ideally with more than one time point, verifications and reflections on qualitative work, robust statistical analysis, appropriate study controls, consideration of confounding variables, and and more transparent reporting of population samples, methodologies, and analyses used. reflections on qualitative work.

Formatted: Font: 11 pt

Formatted: Font: 11 pt

Formatted: Font: Not Italic

Formatted: Font: 11 pt

Review limitations

There are several limitations of the present review pertaining to the search strategy and review process, the limited sample of studies, publication bias, and limitations of the studies themselves. Specifically, the small number of included studies and their varied study aims, design and population samples make generalisations difficult. Grouped demographics, such as middle management, are justified by the overlap between positions. With more literature on this topic, distinctions could be made between job positions. Furthermore, more research on lower levels of management would have provided a better balanced review of hospital managers work and contributions to quality. Restricting the language of studies to English in the search strategy is likely to have biased the findings and misrepresent which countries conduct studies on this topic. There is an over-reliance on perceptions across the studies, which ultimately reduces the validity of the conclusions drawn from their findings. As most of the study findings relied on self-reports, social desirability may have resulted in exaggerated processes and inflated outputs. Although, encouragingly, one of the included studies found that managers that perceived their Boards to be effective in quality oversight were from hospitals that had higher processes-of-care scores and lower risk adjusted mortality. The quality assessment scores should be viewed with caution; such scores are subjective and may not take into consideration factors beyond the quality assessment scale used. Due to the enormity of this review, the publication of this article is some time after the search run date. As is evident from the number of included articles, there is little evidence published on

Formatted: Font: (Default) Arial

Formatted: Font: (Default) Arial

Formatted: Font: (Default) Arial

Formatted: Font: (Default) Arial, Not Italic, Font color: Auto, English (U.K.)

Formatted: Font: (Default) Arial, Not Italic, Font color: Auto, English (U.K.)

Formatted: Font: (Default) Arial, Not Italic, Font color: Auto, English (U.K.)

Formatted: Font: (Default) Arial, Not Italic, Font color: Auto, English (U.K.)

Formatted: Font: (Default) Arial, Not Italic, Font color: Auto, English (U.K.)

For peer review only - http://bmjopen.bmj.com/site/about/guidelines.xhtml

this topic, so we consider this not to greatly impact on the current usefulnessrelevance of tthis articlee review, particularly as the literature reviewed spans almost three decades.

Formatted: Font: (Default) Arial, Not Italic, Font color: Auto, English (U.K.)

Conclusion

The modest literature that exists suggests that managers' time spent, engagement and work can influence quality and safety clinical outcomes, processes and performance. Managerial activities that affect quality performance are especially highlighted by this review, such as engagement in quality, establishing goals and strategy to improve care, setting the quality agenda, engaging in quality, promoting a quality improvement culture, managing resisters, and procurement of organisational resources for quality. Positive actions to consider include the establishment of a Board quality committee with a specific item on quality at the Board meeting, a quality performance measurement report and a dashboard with national quality and safety benchmarks, performance evaluation attached to quality and safety, and an infrastructure for staff-manager interactions on quality strategies. However, the studies additionally show that many of these contextual factorsarrangements and activities awere lacking not in place within the management of quality within the hospital settingstudy samples. There are also indications of a need for managers to devote more time to quality and safety. MMore than one studyMoreover, there are indications of a need to develop managerial knowledge and training on quality and safety and to increase time devoted to quality and safety. The review suggest time spent by the Board should exceed 20-25%, yet the findings expose that certain Boards devote less time than this. There are some indications of a need to develop managerial knowledge and training on this topic. Much of the content of the articles focused on these such contextual factors rather than information on the managerial role itself; more empirical research is required to elucidate managers' actual activities. Research is additionally required to examine middle and frontline managers, non-manager perceptions, and to assess senior managers' time and tasks outside of the Boardroom. We present the IPO model to summarise the evidence-based promotion of

Formatted: Font: Arial

For peer review only - http://bmjopen.bmj.com/site/about/guidelines.xhtml

conditions and activities in order to guide managers on the approaches taken to influence quality performance. More robust empirical research with objective outcome measures could strengthen this guidance.

Formatted: Font: Arial

Formatted: Font: Arial

Formatted: Font: Arial

For peer review only

For peer review only - http://bmjopen.bmj.com/site/about/guidelines.xhtml

- There is a dearth of empirical evidence on hospital managerial work and its influence on quality of care.
- There is some evidence that Boards'/managers' time spent, engagement and work can influence quality and safety clinical outcomes, processes and performance.
- Some variables associated with good quality performance were lacking in study hospitals.
- Many Board managers do not spend sufficient time on quality and safety and need to develop their knowledge on quality and safety.
- There is a greater focus on the contextual issues surrounding managers' roles than on examining managerial activities.
- Research is required to examine middle and frontline managers, to take into consideration non-managers' perceptions, and to assess senior managers' time and tasks outside of the Boardroom. More robust methodologies with objective outcome measures would strengthen the evidence.

Formatted: Line spacing: 1.5 lines, Bulleted + Level: 1 + Aligned at: 0.25" + Indent at:

Formatted: Indent: Left: 0.5", No bullets or numbering

Formatted: Line spacing: 1.5 lines, Bulleted + Level: 1 + Aligned at: 0.25" + Indent at:

Formatted: Font: 11 pt

Box 1 2 Key messages from the systematic literature review

CONTRIBUTORS

All co-authors contributed to the study design and review of drafts of the article. The first author screened all of the articles for inclusion in this review and appraised the study quality. Dr Anna Renz and Miss Dina Grishin screened a sample of these at title/abstract and full text, and Miss Ana Wheelock scored the quality of a sample of the included articles.

For peer review only - http://bmjopen.bmj.com/site/about/guidelines.xhtml

ACKNOWLEDGEMENTS

We would like to thank Miss Dina Grishin for helping to review the abstracts and Miss Ana Wheelock for helping to assess the quality of the articles.

COMPETING INTERESTS

There are no competing interests.

FUNDING

This work was supported by funding from the Health Foundation and the National Institute for Health Research.

Figure Legends

Figure 1 Review stages based on PRISMA Flow Diagram[33]
Figure 2 The Quality Management IPO model

For peer review only

REFERENCES

1. Kizer KW. Patient Safety: A Call to Action: Safety Is a Critical Component of Quality. Medscape General Medicine 2001;**3:10**.
2. Kizer KW. Establishing health care performance standards in an era of consumerism. Journal of the American Medical Association 2001;**286**:1213-7.
3. Berwick D. Will is the way to win the patient safety war. Health Serv J 2007;**117**:18-9.
4. Institute of Medicine. Crossing the quality chasm: a new health system for the 21st century. Washington: National Academy Press; 2001.
5. Gosfield AG, Reinertsen JL. The 100,000 lives campaign: crystallizing standards of care for hospitals. Health aff 2005;**24**:1560–70.
6. Denham CR. Is your hospital as safe as your bank?...Time to ask your board. J Patient Saf 2009;**5**:122-6.
7. Francis R. Report of the Mid Staffordshire NHS Foundation Trust Public Inquiry London: The Stationery Office 2013 [accessed on 18.03.13]. Available from: http://www.midstaffspublicinquiry.com/report.
8. National Advisory Group on the Safety of Patients in England. A promise to learn – a commitment to act: Improving the Safety of Patients in England 2013 [accessed on 15.08.13]. Available from: https://www.gov.uk/government/uploads/system/uploads/attachment_data/file/226703/Berwick_Report.pdf.
9. O'Dea A, Flin R. Site managers and safety leadership in the offshore oil and gas industry. Safety Sci 2001;**37**:39-57.
10. Zohar D, Luria G. The use of supervisory practices as leverage to improve safety behavior: a cross-level intervention model. Journal of Safety Res 2003;**34**:567-77.
11. Flin R. "Danger-Men at Work": Management Influence on Safety. Hum Factor and Ergon Man 2003;**13**:261-8.
12. Guidotti TL. How can managers visibly promote safety and health protection? J Occ Env Med 2010;**52**:1141-2.
13. Conway J. Getting boards on board: engaging governing boards in quality and safety. Jt Comm J Qual Patient Saf 2008;**34**:214-20.
14. Gautam KS. A call for board leadership on quality in hospitals. Qual Manag Health Care 2005;**14**:18-30.
15. Caldwell C, Butler G, Grah J. Breakthrough quality: what the board must do. Trustee 2008;**61**:32-3, 1.
16. Goeschel CA, Wachter RM, Pronovost PJ. Responsibility for quality improvement and patient safety: hospital board and medical staff leadership challenges. Chest 2010;**138**:171-8.
17. McGinn P, Dave R. What is the board's role in quality? Trustee 2007;**60**:40.
18. Øvretveit J. Leading improvement effectively: Review of research 2009 [accessed on 21.12.12]. Available from: http://www.health.org.uk/public/cms/75/76/313/560/Leading%20imrpovement%20effectively.pdf?realName=YUaqJk.pdf.
19. Flin R, Yule S. Leadership for safety: industrial experience. Qual and Saf Health Care 2004;**13**:45-51.

For peer review only - http://bmjopen.bmj.com/site/about/guidelines.xhtml

20. West E. Management matters: the link between hospital organisation and quality of patient care. Qual and Saf Health Care 2001;**10**:40-8.

21. Glickman SW, Baggett KA, Krubert CG, Peterson ED, Schulman KA. Promoting quality: the health-care organization from a management perspective. Int J Qual Health Care 2007;**19**:341-8.

22. Gaucher E, Kratochwill EW. The leader's role in implementing total quality management. Qual Manag Health Care 1993;**1**:10-8.

23. Thorman KE, Capitulo KL, Dubow J, Hanold K, Noonan M, Wehmeyer J. Perinatal patient safety from the perspective of nurse executives: a round table discussion. JOGNN 2006;**35** 409-16.

24. Aspden P, Corrigan JM, Wolcott J, Erickson SM. Patient safety: achieving a new standard of care. Institute of Medicine, editor. Washington: National Academies Press; 2004.

25. AHRQ. AHRQ Patient Safety Network Glossary 2011 [accessed on 19.05.12]. Available from: http://www.psnet.ahrq.gov/glossary.aspx?indexLetter=P.

26. Merali F. NHS managers' commitment to a socially responsible role: the NHS managers' views of their core values and their public image. Social Responsibility Journal 2005;**1**:38-46.

27. Mintzberg H. The Nature of Managerial Work. New York: Harper & Row; 1973.

28. Tanon AA, Champagne F, Contandriopoulos AP, Pomey MP, Vadeboncoeur A, Nguyen H. Patient safety and systematic reviews: finding papers indexed in MEDLINE, EMBASE and CINAHL. Quality saf health care 2010;**19**:452-61.

29. Cohen J. A coefficient for agreement for nominal scales. Educ Psych Meas 1960;**20**:37–46.

30. Cicchetti DV, Feinstein AR. High agreement but low kappa: II. Resolving the paradoxes. J Clin Epidemiol 1990;**43**:551-8.

31. Viera AJ, Garrett JM. Understanding interobserver agreement: the kappa statistic. Family med 2005;**37**:360-3.

32. Baker GR, Denis JL, Pomey MP, MacIntosh-Murray A. Effective governance for quality and patient safety in Canadian Healthcare organizations: a report to the Canadian Health Services Research Foundation and the Canadian Patient Safety Institute. Ottawa: Canadian Health Services Research Foundation and Canadian Patient Safety Institute, 2010.

33. Moher D, Liberati A, Tetzlaff J, Altman DG. Preferred reporting items for systematic reviews and meta-analyses: the PRISMA statement. Ann Intern Med 2009;**151**:264-9.

34. Kmet LM, Lee RC, Cook LS. Standard Quality Assessment Criteria for Evaluating Primary Research Papers from a Variety of Fields. Edmonton: Alberta Heritage Foundation for Medical Research, 2004.

35. Joshi MS, Hines SC. Getting the board on board: Engaging hospital boards in quality and patient safety. Jt Comm J Qual Patient Saf 2006;**32**:179-87.

36. Mastal MF, Joshi M, Schulke K. Nursing leadership: championing quality and patient safety in the boardroom. Nurs Econ 2007;**25**:323-30.

37. Jiang HJ, Lockee C, Bass K, Fraser I. Board engagement in quality: findings of a survey of hospital and system leaders. J Healthc Manag 2008;**53**:121-34.

38. Jiang HJ, Lockee C, Bass K, Fraser I. Board oversight of quality: Any differences in process of care and mortality? J Healthc Manag 2009;**54**:15-29.

39. Walsh D, Downe S. Meta-synthesis method for qualitative research: a literature review. J Adv Nurs 2005;**50**:204-11.

40. Bradley EH, Webster TR, Schlesinger M, Baker D, Inouye SK. The roles of senior management in improving hospital experiences for frail older adults. J Healthc Manag 2006;**51**:323-36.

For peer review only - http://bmjopen.bmj.com/site/about/guidelines.xhtml

41. Caine C, Kenrick M. The role of clinical directorate managers in facilitating evidence-based practice: a report of an exploratory study. J Nurs Manag 1997;**5**:157-65.

42. Fox RT, Fox DH, Wells PJ. Performance of first-line management functions on productivity of hospital unit personnel. J Nurs Adm 1999;**29**:12-8.

43. Harris A. Risk management in practice: how are we managing? Clin Perform Qual Health Care 2000;**8**:142-9.

44. Saint S, Kowalski CP, Banaszak-Holl J, Forman J, Damschroder L, Krein SL. The importance of leadership in preventing healthcare-associated infection: Results of a multisite qualitative study. Infect Control Hosp Epidemiol 2010;**31**:901-7.

45. Poniatowski L, Stanley S, Youngberg B. Using information to empower nurse managers to become champions for patient safety. Nurs Adm Q 2005;**29**:72-7.

46. Balding C. Embedding organisational quality improvement through middle manager ownership. Int J Health Care Qual Assur 2005;**18**:271-88.

47. Bradley EH, Holmboe ES, Mattera JA, Roumanis SA, Radford MJ, Krumholz HM. The roles of senior management in quality improvement efforts: what are the key components? J Healthc Manag 2003;**48**:15-28.

48. Levey S, Vaughn T, Koepke M, Moore D, Lehrman W, Sinha S. Hospital Leadership and Quality Improvement: Rhetoric Versus Reality. J Patient Saf 2007;**3**:9-15.

49. Vaughn T, Koepke M, Kroch E, Lehrman W, Sinha S, Levey S. Engagement of Leadership in Quality Improvement Initiatives: Executive Quality Improvement Survey Results. J Patient Saf 2006;**2**:2-9.

50. Jha A, Epstein A. Hospital governance and the quality of care: Health Aff 2010;29:182-187.

51. Prybil LD, Peterson R, Brezinski P, Zamba G, Roach Jr W, Fillmore A. Board oversight of patient care quality in community health systems. Am J Med Qual 2010;**25**:34-41.

52. Braithwaite J, Finnegan TP, Graham EM, Degeling PJ, Hindle D, Westbrook MT. How important are quality and safety for clinician managers? Evidence from triangulated studies. Clin Governance 2004;**9**:34-41.

53. Weingart SN, Page D. Implications for practice: challenges for healthcare leaders in fostering patient safety. Qual Saf Health Care 2004;**13**:52-6.

54. Flin R, Maran N. Identifying and training non-technical skills for teams in acute medicine. Qual Saf Health Care 2004;**13**:80-4.

55. Kunzle B, Kolbe M, Grote G. Ensuring patient safety through effective leadership behaviour: A literature review. Safety Sci 2010;**48**:1-17.

56. Naranjo-Gil D. Strategic performance in hospitals: the use of the balanced scorecard by nurse managers. Health Care Manage Rev 2009;**34**:161-70.

57. Weiner BJ, Alexander JA, Shortell SM. Leadership for quality improvement in health care; empirical evidence on hospital boards, managers, and physicians. Med Care Res Rev 1996;**53**:397-416.

58. Edwards N, Marshall M, McLellan A, Abbasi K. Doctors and managers: A problem without a solution? BMJ 2003;**326**:609-10.

59. Wilkinson JE, Powell A, Davies H. Are clinicians engaged in quality improvement? A review of the literature on healthcare professionals' views on quality improvement initiative: Health Foundation Report; 2011 [accessed on 21.12.12]. Available from: http://www.health.org.uk/public/cms/75/76/313/2440/Are%20clinicians%20engaged%20in%20quality%20improvement.pdf?realName=y8NfS4.pdf.

For peer review only - http://bmjopen.bmj.com/site/about/guidelines.xhtml

60. Jha AK, Epstein AM. A Survey Of Board Chairs Of English Hospitals Shows Greater Attention To Quality Of Care Than Among Their US Counterparts. Health Aff 2013;**32**:677-85.

61. Stanton P. The role of an NHS Board in assuring the quality of clinically governed care and the duty of trust to patients. Clin Governance 2006;**11**:39-49.

62. Lukas CV, Holmes SK, Cohen AB, Restuccia J, Cramer IE, Shwartz M, et al. Transformational change in health care systems: an organizational model. Health Care Manage Rev 2007;**32**:309-20.

63. Shortell SM, Bennett CL, Byck GR. Assessing the impact of continuous quality improvement on clinical practice: what it will take to accelerate progress. The Milbank Q 1998;**76**:593-624, 510.

64. Keroack MA, Youngberg BJ, Cerese JL, Krsek C, Prellwitz LW, Trevelyan EW. Organizational factors associated with high performance in quality and safety in academic medical centers. Acad Med 2007;**82**:1178-86.

65. Parker VA, Wubbenhorst WH, Young GJ, Desai KR, Charns MP. Implementing quality improvement in hospitals: the role of leadership and culture. Am J Medical Qual 1999;**14**:64-9.

66. Weiner B, Shortell S, Alexander J. Promoting clinical involvement in hospital quality improvement efforts: the effects on top management, board, and physician leadership. Health Serv Res 1997;**32**:491-510.

67. Duckers MLA, Stegeman I, Spreeuwenberg P, Wagner C, Sanders K, Groenewegen PP. Consensus on the leadership of hospital CEOs and its impact on the participation of physicians in improvement projects. Health Policy 2009;**91**:306-13.

68. Vogus TJ, Sutcliffe KM. The impact of safety organizing, trusted leadership, and care pathways on reported medication errors in hospital nursing units. Med Care 2007;**45**:997-1002.

Formatted: Line spacing: Multiple 1.15 li

For peer review only

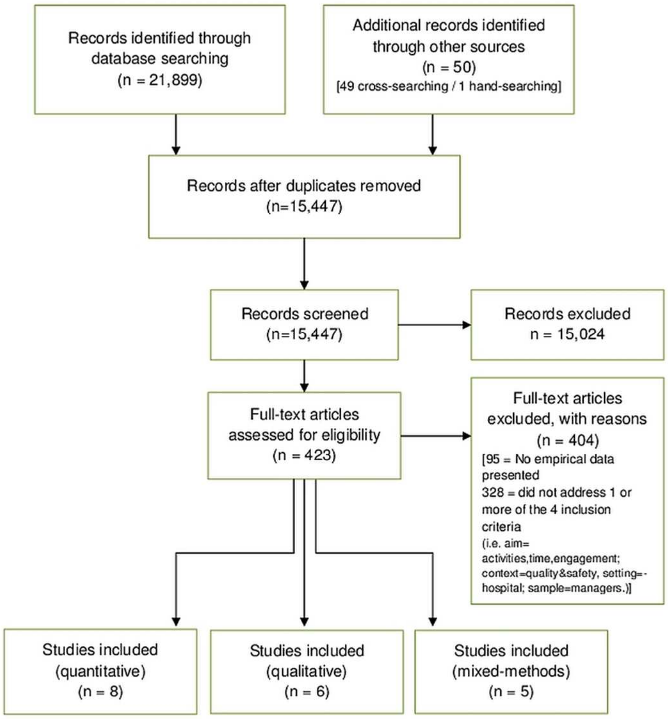

Figure 1 Review stages based on PRISMA Flow Diagram[33]
90x127mm (300 x 300 DPI)

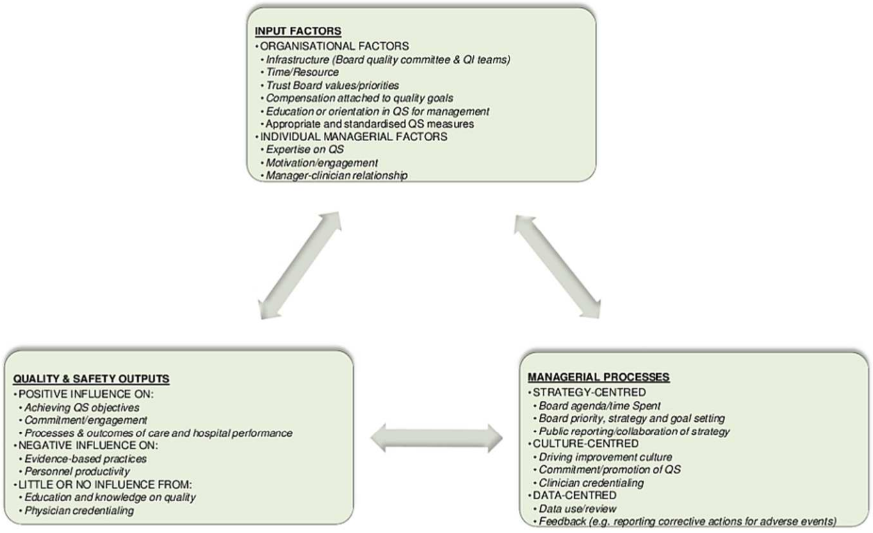

Figure 2 The Quality Management IPO model
90x63mm (300 x 300 DPI)

view only

APPENDIX 1

The Cochrane Library search strategy:

ID	Search
#1	MeSH descriptor: [Recombinant Fusion Proteins] explode all trees
#2	MeSH descriptor: [Antibodies, Monoclonal] explode all trees
#3	MeSH descriptor: [Receptors, Tumor Necrosis Factor] explode all trees
#4	MeSH descriptor: [Receptors, Interleukin-1] explode all trees
#5	MeSH descriptor: [Receptors, Interleukin-6] explode all trees
#6	MeSH descriptor: [Monokines] explode all trees
#7	monoclonal antibody ca2
#8	TNFR-Fc fusion protein
#9	MeSH descriptor: [Interleukin 1 Receptor Antagonist Protein] explode all trees
#10	etanercept
#11	enbrel
#12	infliximab
#13	remicade
#14	adalimumab
#15	humira
#16	D2E7
#17	anakinra
#18	kineret
#19	antril
#20	abatacept
#21	CTLA4Ig
#22	orencia
#23	rituximab
#24	rituxan
#25	idec c2b8
#26	golimumab
#27	simponi
#28	cnto-148
#29	tocilizumab
#30	atlizumab
#31	actemra
#32	roactemra
#33	certolizumab
#34	CDP870
#35	cimzia
#36	"TNFR:Fc":ti,ab,kw (Word variations have been searched)
#37	tofacitinib:ti,ab,kw (Word variations have been searched)

For peer review only

#38	MeSH descriptor: [Janus Kinases] explode all trees
#39	Xeljanz:ti,ab,kw (Word variations have been searched)
#40	#1 or #2 or #3 or #4 or #5 or #6 or #7 or #8 or #9 or #10 or #11 or #12 or #13 or #14 or #15 or #16 or #17 or #18 or #19 or #20 or #21 or #22 or #23 or #24 or #25 or #26 or #27 or #28 or #29 or #30 or #31 or #32 or #33 or #34 or #35 or #36 or #37 or #38 or #39
#41	MeSH descriptor: [Arthritis, Rheumatoid] explode all trees
#42	Rheumatoid:ti or Rheumatoid:ab (Word variations have been searched)
#43	arthriti*:ti or arthriti*:ab (Word variations have been searched)
#44	#42 and #43
#45	#41 or #44
#46	#40 and #45 in Trials

EMBASE search strategy:

1	abatacept.mp.
2	adalimumab.mp.
3	certolizumab.mp.
4	etanercept.mp.
5	CDP870.mp.
6	golimumab.mp.
7	infliximab.mp.
8	rituximab.mp.
9	tocilizumab.mp.
10	humira.mp.
11	trudexa.mp.
12	orencia.mp.
13	cimzia.mp.
14	enbrel.mp.
15	simponi.mp.
16	rituxan.mp.
17	mabthera.mp.
18	actemra.mp.
19	RoActemra.mp.
20	monoclonal antibodies.mp. or exp Antibodies, Monoclonal/
21	exp Monokines/

For peer review only

22	exp Receptors, Interleukin-1/
23	exp Receptors, Interleukin-6/
24	exp Polyethylene Glycols/
25	exp Immunoglobulin G/
26	exp Immunoconjugates/
27	immunoglobulin fab fragments.mp. or exp Immunoglobulin Fab Fragments/
28	t-lymphocytes.mp. or exp T-Lymphocytes/
29	exp tumor necrosis factor inhibitor/
30	exp interleukin 1 receptor blocking agent/
31	D2E7.mp.
32	anakinra.mp.
33	kineret.mp.
34	antril.mp.
35	CTLA4Ig.mp.
36	idec c2b8.mp.
37	cnto-148.mp.
38	atlizumab.mp.
39	tofacitinib.mp.
40	exp Janus kinase inhibitor/
41	*tumor necrosis factor receptor/dt [Drug Therapy]
42	or/1-41
43	exp Random Allocation/
44	exp Single-Blind Method/
45	exp Double-Blind Method/
46	Placebo.mp.
47	Randomi?ed controlled trial$.mp.
48	rct.mp.
49	Random allocation.mp.
50	Randomly allocated.mp.
51	Allocated randomly.mp.
52	(allocated adj2 random).mp.
53	Single blind$.mp.
54	Double blind$.mp.
55	((treble or triple) adj blind$).mp.
56	Placebo$.mp.

For peer review only

57	or/43-56
58	rheumatoid.ti,ab.
59	*rheumatoid arthritis/
60	58 or 59
61	42 and 57 and 60
62	limit 61 to (book or book series or conference abstract or conference paper or conference proceeding or "conference review")
63	61 not 62

LILACS search strategy:

(tw:(tw:((tw:(rheumatoid)) AND (tw:(etanercept)) OR (tw:(enbrel)) OR (tw:(infliximab)) OR (tw:(remicade)) OR (tw:(adalimumab)) OR (tw:(humira)) OR (tw:(d2e7)) OR (tw:(anakinra)) OR (tw:(kineret)) OR (tw:(antril)) OR (tw:(abatacept)) OR (tw:(ctla4ig)) OR (tw:(orencia)) OR (tw:(rituximab)) OR (tw:(rituxan)) OR (tw:(idec c2b8)) OR (tw:(golimumab)) OR (tw:(simponi)) OR (tw:(cnto-148)) OR (tw:(tocilizumab)) OR (tw:(atlizumab)) OR (tw:(actemra)) OR (tw:(roactemra)) OR (tw:(certolizumab)) OR (tw:(cdp870)) OR (tw:(cimzia)) OR (tw:(tnfr:fc)) OR (tw:(tofacitinib)) OR (tw:(janus kinases)) OR (tw:(xeljanz))) AND db:("LILACS"))) AND (tw:(random*))

For peer review only

PRISMA 2009 Checklist

Section/topic	#	Checklist item	Reported on page #
**TITLE**			
Title	1	Identify the report as a systematic review, meta-analysis, or both.	1
**ABSTRACT**			
Structured summary	2	Provide a structured summary including, as applicable: background; objectives; data sources; study eligibility criteria, participants, and interventions; study appraisal and synthesis methods; results; limitations; conclusions and implications of key findings; systematic review registration number.	2
**INTRODUCTION**			
Rationale	3	Describe the rationale for the review in the context of what is already known.	4
Objectives	4	Provide an explicit statement of questions being addressed with reference to participants, interventions, comparisons, outcomes, and study design (PICOS).	NA (not a review of interventions)
**METHODS**			
Protocol and registration	5	Indicate if a review protocol exists, if and where it can be accessed (e.g., Web address), and, if available, provide registration information including registration number.	All protocol info in appendices
Eligibility criteria	6	Specify study characteristics (e.g., PICOS, length of follow-up) and report characteristics (e.g., years considered, language, publication status) used as criteria for eligibility, giving rationale.	7
Information sources	7	Describe all information sources (e.g., databases with dates of coverage, contact with study authors to identify additional studies) in the search and date last searched.	6
Search	8	Present full electronic search strategy for at least one database, including any limits used, such that it could be repeated.	in appendices
Study selection	9	State the process for selecting studies (i.e., screening, eligibility, included in systematic review, and, if applicable, included in the meta-analysis).	7
Data collection process	10	Describe method of data extraction from reports (e.g., piloted forms, independently, in duplicate) and any processes for obtaining and confirming data from investigators.	10
Data items	11	List and define all variables for which data were sought (e.g., PICOS, funding sources) and any assumptions and simplifications made.	6
Risk of bias in individual studies	12	Describe methods used for assessing risk of bias of individual studies (including specification of whether this was done at the study or outcome level), and how this information is to be used in any data synthesis.	10
Summary measures	13	State the principal summary measures (e.g., risk ratio, difference in means).	NA
Synthesis of results	14	Describe the methods of handling data and combining results of studies, if done, including measures of consistency (e.g., I^2) for each meta-analysis.	10

For peer review only

PRISMA 2009 Checklist

Section/topic	#	Checklist item	Reported on page #
Risk of bias across studies	15	Specify any assessment of risk of bias that may affect the cumulative evidence (e.g., publication bias, selective reporting within studies).	10
Additional analyses	16	Describe methods of additional analyses (e.g., sensitivity or subgroup analyses, meta-regression), if done, indicating which were pre-specified.	NA
**RESULTS**			
Study selection	17	Give numbers of studies screened, assessed for eligibility, and included in the review, with reasons for exclusions at each stage, ideally with a flow diagram.	9
Study characteristics	18	For each study, present characteristics for which data were extracted (e.g., study size, PICOS, follow-up period) and provide the citations.	16
Risk of bias within studies	19	Present data on risk of bias of each study and, if available, any outcome level assessment (see item 12).	16
Results of individual studies	20	For all outcomes considered (benefits or harms), present, for each study: (a) simple summary data for each intervention group (b) effect estimates and confidence intervals, ideally with a forest plot.	NA
Synthesis of results	21	Present results of each meta-analysis done, including confidence intervals and measures of consistency.	NA
Risk of bias across studies	22	Present results of any assessment of risk of bias across studies (see Item 15).	11
Additional analysis	23	Give results of additional analyses, if done (e.g., sensitivity or subgroup analyses, meta-regression [see Item 16]).	NA
**DISCUSSION**			
Summary of evidence	24	Summarize the main findings including the strength of evidence for each main outcome; consider their relevance to key groups (e.g., healthcare providers, users, and policy makers).	24
Limitations	25	Discuss limitations at study and outcome level (e.g., risk of bias), and at review-level (e.g., incomplete retrieval of identified research, reporting bias).	26
Conclusions	26	Provide a general interpretation of the results in the context of other evidence, and implications for future research.	27
**FUNDING**			
Funding	27	Describe sources of funding for the systematic review and other support (e.g., supply of data); role of funders for the systematic review.	30

From: Moher D, Liberati A, Tetzlaff J, Altman DG, The PRISMA Group (2009). Preferred Reporting Items for Systematic Reviews and Meta-Analyses: The PRISMA Statement. PLoS Med 6(6): e1000097. doi:10.1371/journal.pmed1000097

For more information, visit: **www.prisma-statement.org**.

For peer review only - http://bmjopen.bmj.com/site/about/guidelines.xhtml

The Role of Hospital Managers in Quality and Patient Safety. A Systematic Review

Journal:	*BMJ Open*
Manuscript ID:	bmjopen-2014-005055.R2
Article Type:	Research
Date Submitted by the Author:	19-Aug-2014
Complete List of Authors:	Parand, Anam; Imperial College London, Surgery and cancer<br>Dopson, Sue; University of Oxford, Said Business School<br>Renz, Anna; Imperial College London, Surgery and cancer<br>Vincent, Charles; University of Oxford, Experimental Psychology
**Primary Subject Heading**:	Medical management
Secondary Subject Heading:	Health services research
Keywords:	HEALTH SERVICES ADMINISTRATION & MANAGEMENT, Quality in health care < HEALTH SERVICES ADMINISTRATION & MANAGEMENT, Systematic literature review, Patient Safety, Leadership

For peer review only - http://bmjopen.bmj.com/site/about/guidelines.xhtml

The Role of Hospital Managers in Quality and Patient Safety. A Systematic Review

Anam Parand[1], Sue Dopson[2], Anna Renz[1], Charles Vincent[3]

[1]Department of Surgery & Cancer, Imperial College London, London, UK
[2]Said Business School, University of Oxford, Oxford, UK
[3]Department of Experimental Psychology, University of Oxford, Oxford, UK

Correspondence to: Anam Parand, Department of Surgery & Cancer, Imperial College London, 503, 5th Floor Wright Fleming Building, St Mary's Campus, Norfolk Place, London W2 1PG, UK.
Email: a.parand@imperial.ac.uk; Tel: +44 (0) 207594 9726; Fax: +44 (0)20 7594 9726

Key Words: Quality of health care; Patient safety; Systematic literature review; Leadership, Management.

Abstract Word Count: 236 words

Manuscript Word Count: 5,329 words (excluding title page, abstract, references, acknowledgements and tables).

For peer review only - http://bmjopen.bmj.com/site/about/guidelines.xhtml

For peer review only

ABSTRACT

Objectives: To review the empirical literature to identify the activities, time spent and engagement of hospital managers in quality of care.

Design: A systematic review of the literature.

Methods: A search was carried out on the databases MEDLINE, PSYCHINFO, EMBASE, HMIC. The search strategy covered three facets: management, quality of care, and the hospital setting comprising medical subject headings and key terms. Reviewers screened 15,447 titles/abstracts and 423 full texts were checked against inclusion criteria. Data extraction and quality assessment were performed on 19 included articles.

Results: The majority of studies were set in the US and investigated Board/senior level management. The most common research designs were interviews and surveys on the perceptions of managerial quality and safety practices. Managerial activities comprised strategy, culture and data-centred activities, such as driving improvement culture and promotion of quality, strategy/goal-setting and providing feedback. Significant positive associations with quality included compensation attached to quality, using quality improvement measures and having a Board quality committee. However there is an inconsistency and inadequate employment of these conditions and actions across the sample hospitals.

Conclusion: There is some evidence that managers' time spent and work can influence quality and safety clinical outcomes, processes and performance. However, there is a dearth of empirical studies, further weakened by a lack of objective outcome measures and little examination of actual actions undertaken. We present a model to summarise the conditions and activities that affect quality performance.

For peer review only - http://bmjopen.bmj.com/site/about/guidelines.xhtml

ARTICLE SUMMARY

Strengths and limitations of this study

- This is the first systematic review of the literature that has considered the evidence on Boards' and managers' time spent, engagement and work within the context of quality and safety. This review adds to the widely anecdotal and commentary pieces that speculate on what managers should be doing by presenting what they are actually doing.
- The review reveals conditions and actions conducive to good quality management and offers a model to transparently present these to managers considering their own part in quality and safety.
- The search for this review has screened a vast amount of the literature (over 15,000 articles) across a number of databases.
- The small number of included studies and their varied study aims, design and population samples make generalisations difficult. With more literature on this topic, distinctions could be made between job positions.
- The quality assessment scores are subjective and may not take into consideration factors beyond the quality assessment scale used.

For peer review only - http://bmjopen.bmj.com/site/about/guidelines.xhtml

For peer review only

INTRODUCTION

Managers in healthcare have a legal and moral obligation to ensure a high quality of patient care and to strive to improve care. These managers are in a prime position to mandate policy, systems, procedures and organisational climates. Accordingly, many have argued that it is evident that healthcare managers possess an important and obvious role in quality of care and patient safety and that it is one of the highest priorities of healthcare managers.[1-3] In line with this, there have been calls for Boards to take responsibility for quality and safety outcomes.[4, 5] One article warned hospital leaders of the dangers of following in the path of bankers falling into recession, constrained by their lack of risk awareness and reluctance to take responsibility.[6] To add to the momentum are some high profile publicity of hospital management failures affecting quality and safety, eliciting strong instruction for managerial leadership for quality at the national level in some countries.[7, 8]

Beyond healthcare, there is clear evidence of managerial impact on workplace safety.[9-12] Within the literature on healthcare, there are non-empirical articles providing propositions and descriptions on managerial attitudes and efforts to improve safety and quality. This literature, made up of opinion articles, editorials and single participant experiences, present an array of insightful suggestions and recommendations for actions that hospital managers should take to improve the quality of patient care delivery in their organisation.[13-17] However, researchers have indicated that there is a limited evidence-base on this topic.[18-21] Others highlight the literature focus on the difficulties of the managers' role and the negative results of poor leadership on quality improvement (QI) rather than considering actions that managers presently undertake on quality and safety.[22, 23] Consequently, little is known about what healthcare managers are doing in practice to ensure and improve quality of care and patient safety, how much time they spend on this, and what research-based guidance is available for managers in order for them to decide on appropriate areas to become involved. Due perhaps to the broad nature of the topic, scientific studies exploring

For peer review only - http://bmjopen.bmj.com/site/about/guidelines.xhtml

these acts and their impact are likely to be a methodological challenge, although a systematic review of the evidence on this subject is notably absent. This present systematic literature review aims to identify empirical studies pertaining to the role of hospital managers in quality of care and patient safety. We define 'role' to comprise of managerial activities, time spent and active engagement in quality and safety and its improvement. Whilst the primary research question is on the managers' role, we take into consideration the contextual factors surrounding this role and its impact or importance as highlighted by the included studies. Our overarching question is 'What is the role of hospital managers in quality and safety and its improvement?' The specific review research questions are as follows:

- How much time is spent by hospital managers on quality and safety and its improvement?
- What are the managerial activities that relate to quality and safety and its improvement?
- How are managers engaged in quality and safety and its improvement?
- What impact do managers have on quality and safety and its improvement?
- How do contextual factors influence the managers' role and impact on quality and safety and its improvement?

METHODS

Concepts and definitions

Quality of care and patient safety were defined on the basis of widely accepted definitions from the Institute of Medicine (IOM) and the Agency for Healthcare Research and Quality Patient Safety Network (ARQH PSN). IOM define quality in healthcare as possessing the

For peer review only - http://bmjopen.bmj.com/site/about/guidelines.xhtml

following dimensions: safe, effective, patient-centred, timely, efficient and equitable.[4] They define patient safety simply as *"the prevention of harm to patients",*[24] and AHRQ define it is *"freedom from accidental or preventable injuries produced by medical care."*[25] Literature was searched for all key terms associated with quality and patient safety to produce an all-encompassing approach. A manager was defined as an employee that has subordinates, oversees staff, is responsible for staff recruitment and training, and holds budgetary accountabilities. Therefore, all levels of managers including Boards of managers were included in this review with the exception of clinical frontline employees, e.g. doctors or nurses, who may have taken on further managerial responsibilities alongside their work but do not have a primary official role as a manager. Those that have specifically taken on a role for quality of care, e.g. the modern matron, were also excluded. Distinction between senior, middle and frontline management were as follows: senior management hold Trust-wide responsibilities;[26] middle managers are in the middle of the organisational hierarchy chart and have one or more managers reporting to them;[27] frontline managers are defined as managers at the first level of the organisational hierarchy chart who have frontline employees reporting to them. Board managers include all members of the Board. Although, there are overlaps between senior managers and Boards (for example Chief Executive Officers (CEOs) may sit on hospital Boards), we aim to present senior and Board level managers separately due to the differences in their responsibilities and position. Only managers that would manage within or govern hospitals were included, with the exclusion of settings that solely served mental health or that comprised solely of non-acute care community services (in order to keep the sample more homogenous). The definition of 'Role' focused on actual engagement, time spent and activities that do or did occur rather than those recommended that should or could occur.

For peer review only - http://bmjopen.bmj.com/site/about/guidelines.xhtml

Search Strategy

Literature was reviewed between 01 Jan 1983 and 01 Nov 2010. Eligible articles were those that described or tested managerial roles pertaining to quality and safety in the hospital setting. Part of the search strategy was based on guidance by Tanon et al (2010).[28] EMBASE, MEDLINE, Health Management Information Consortium (HMIC) and PSYCHINFO databases were searched. The search strategy involved three facets (i.Management; ii.Quality; iii.Hospital Setting) and five steps. A facet (i.e. a conceptual grouping of related search terms) for role was not included in the search strategy, as it would have significantly reduced the sensitivity of the search.

Multiple iterations and combinations of all search terms were tested to achieve the best level of specificity and sensitivity. In addition to the key terms, Medical Subject Headings (MeSH®) terms were used, which were 'exploded' to include all MeSH subheadings. All databases required slightly different MeSH terms (named Emtree in Embase), therefore four variations of the search strategies were used (see online Appendix 1 for the search strategies). Additional limits placed on the search strategy restricted study subjects to human and the language to English. The search strategy identified 15,447 articles after duplicates had been removed.

Screening

Three reviewers (AP, AR and DG) independently screened the titles and abstracts of the articles for studies that fit the inclusion criteria. One reviewer (AP) screened all 15,447 articles, while two additional reviewers screened 30% of the total sample retrieved from the search strategy: AR screened 20% and DG screened 10%. On testing inter-rater reliability, Cohen's kappa correlations showed low agreement between AR and AP ($K=0.157$, $P<0.01$) and between DG and AP ($K=0.137$, $P<0.00$).[29] However, there was a high percentage of agreement between raters (95% and 89% respectively), which reveals a good inter-rater

For peer review only - http://bmjopen.bmj.com/site/about/guidelines.xhtml

reliability.[30, 31] Discrepancies were resolved by discussion and consensus. The main inclusion criteria were that: the setting was hospitals; the population sample reported on were managers; the context was quality and safety; the aim was to identify the managerial activities/time/engagement in quality and safety. The full inclusion/exclusion criteria and screening tool can be accessed in the online Appendices 2-3. Figure 1 presents the numbers of articles included and excluded at each stage of the review process.

Four hundred and twenty-three articles remained for full text screening. One reviewer (AP) screened all articles and a second reviewer (AR) reviewed 7% of these. A moderate agreement inter-rater reliability score was calculated (K=0.615,P<0.001) with 73% agreement. The primary reoccurring difference in agreement was regarding whether the article pertained to quality of care, owing to the broad nature of the definition. Each article was discussed individually until a consensus was reached on whether to include or exclude. Hand searching and cross-referencing were carried out in case articles were missed by the search strategy or from restriction of databases. One additional article was identified from hand searching,[32] totalling 19 articles included in the systematic review.

Enter Figure 1

Data extraction & methodological quality

The characteristics and summary findings of the 19 included studies are presented in Table 1. This Table is a simplified version of a standardised template that was used to ensure consistency in data extracted from each article. Each study was assessed using a quality appraisal tool developed by Kmet et al (2004),[34] which comprised of two checklists (qualitative and quantitative). Random included articles (32%) were scored by AW for

For peer review only - http://bmjopen.bmj.com/site/about/guidelines.xhtml

scoring consistency. All articles were scored on up to 24 questions with a score between 0-2; Box 1 shows an example definition of what constitutes ‘Yes’ (2), ‘Partial’ (1) and ‘No’ (0) rating criteria. The total percentage scores for each study are presented in Table 1. All studies were included regardless of their quality scores. Some cumulative evidence bias may results from two larger datasets split into more than one study each.[35-38] Through a narrative synthesis, we aimed to maintain the original meanings, interpretations and raw data offered by the articles.[39]

Rating	Criteria to verify whether question or objective is sufficiently described
**Yes**	Is easily identified in the introductory section (or first paragraph of methods section). Specifies (where applicable, depending on study design) *all* of the following: purpose, subjects/target population, and the *specific* intervention(s) /association(s)/descriptive parameter(s) under investigation. A study purpose that only becomes apparent after studying other parts of the paper is *not* considered sufficiently described.
**Partial**	Vaguely/incompletely reported (e.g. “describe the effect of” or “examine the role of” or “assess opinion on many issues” or “explore the general attitudes”...); *or* some information has to be gathered from parts of the paper other than the introduction/background/objective section.
**No**	Question or objective is not reported, or is incomprehensible.
**N/A**	Should not be checked for this question

Box 1 Example of a rating criteria for Kmet’s quality assessment[34]

RESULTS

This results section provides an overview description of the reviewed studies and their key findings. The findings are considered under four main headings: managerial time spent on quality and safety; managerial quality and safety activities; managerial impact on quality and safety; and contextual factors related to managers’ quality and safety role. The section ends with a proposed model to summarise the review findings.

For peer review only - http://bmjopen.bmj.com/site/about/guidelines.xhtml

Description of the studies

From the 19 included studies, the majority were carried out and set in the US (14 studies), and investigated senior management and/or Boards (13 studies). Of these, 3 focused on senior managers alone (e.g. Chief Nursing Officers), 9 concentrated on Board managers and 1 included a mixture of managerial levels. Only 3 investigated middle managers and 3 examined frontline staff (e.g. clinical directorate managers and unit nurse managers). The settings of the study were mostly Trust or hospital-wide; a few articles were set in specific settings or contexts: elderly care,[40] evidence-based medicine,[41] staff productivity,[42] clinical risk management,[43] and hospital acquired infection prevention.[44] Two studies involved specific interventions,[45, 46] and 7 studies concentrated specifically on QI rather than quality and safety oversight or routine.[35, 40, 45-49] There were a mixture of 6 qualitative design (interviews or focus groups); 8 quantitative survey designs; and 5 mix-methods designs. All but one study employed a cross-sectional design[46].[46] The primary outcome measure used in most studies was perceptions of managerial quality and safety practices. All reported participant perceptions and a majority presented self-reports, that is, either a mixture of self and peer reports, or self-reports alone.[41, 43, 45, 46] Several studies asked participants about their own and/or other managers' involvement in regards to their specific quality improvement intervention or quality/safety issue.[40, 41, 44-47] With some variations, the most common research design was to interview or survey senior manager/Board members (particularly Board chairs, presidents and CEOs) perceptions on the Board/senior managers' functions, practices, priorities, agenda, time spent, engagement, challenges/issues, drivers and literacy (e.g. familiarity of key reports) on quality and safety.[35-38, 48-51] Five of these studies included objective process/outcome measures, such as adjusted mortality rates.[35, 37, 38, 49, 50] No other studies included clinical outcome measures.

The quality assessment scores ranged between 50-100%; one study scored (what we consider to be) very low (i.e. <55%), eight studies scored highly (i.e. >75%), two other

For peer review only - http://bmjopen.bmj.com/site/about/guidelines.xhtml

articles scored highly on one out of two of their studies (quantitative/qualitative), and the remaining eight scored a moderate rating in-between. Almost half of the articles did not adequately describe their qualitative studies. Specifically, 8 failed to fully describe their qualitative data collection methods, often not mentioning a standardised topic guide, what questions were asked of participants, or no mention of consent and confidentiality assurances. In 7 studies there was no or vague qualitative data analysis description, including omitting the type of qualitative analysis used. Six of the studies showed no or poor use of verification procedures to establish credibility and 9 reported no or poor reflexivity. Positively, all study designs were evident, the context of studies were clear and the authors showed a connection to a wider body of knowledge.

Similarly to the qualitative studies, 7 quantitative studies did not fully describe, justify or use appropriate analysis methods. However, compared with the qualitative studies, the quantitative studies suffered more from sampling issues. Three studies had particularly small samples (e.g. n=35) and one had an especially low response rate of 15%. Subject characteristics were insufficiently described in 5 studies; in one case the authors did not state the number of hospitals included in data analysis. Several studies had obtained ordinal data but only presented percentages, and only one study appropriately controlled for confounding variables. Across all articles, all but 3 studies reported clear objectives and asserted conclusions clearly supported by the data.

Managerial time spent on quality and safety

The studies on Board level managers highlight an inadequate prioritisation of quality and patient safety on the Board agenda and subsequent time spent at Board meetings. Not all hospitals consistently have quality on their Board agenda, for example CEOs and chairpersons across 30 organisations reported that approximately a third of all Board meetings had quality on their agenda,[35] and necessary quality items were not consistently

For peer review only - http://bmjopen.bmj.com/site/about/guidelines.xhtml

and sometimes never addressed.[36] In all studies examining time spent on quality and safety by the Board, less than half of the total time was spent on quality and safety,[32, 37, 38, 48-51] with a majority of Boards spending 25% or less on quality.[32, 38, 45, 49-51] Findings imply that this may be too low to have a positive influence on quality and safety, as higher quality performance was demonstrated by Boards that spent above 20%/25% on quality.[49, 50] Board members recognised that the usual time spent is insufficient.[48] However, few reported financial goals as more important than quality and safety goals,[32] and health system Boards only spent slightly more time on financial issues than quality.[51] Similar issues are noted by studies on frontline managers; specifically that they placed less time and importance on QI,[42] identified as the least discussed topic by clinical managers.[52]

Managerial quality and safety activities

A broad range of quality-related activities were identified to be undertaken by managers. These are presented by the following three groupings: strategy-centred; data-centred and culture-centred.

Strategy-centred

Board priority-setting and planning strategies aligned with quality and safety goals were identified as Board managerial actions carried out in several studies. High percentages (over 80% in two studies) of Boards had formally established strategic goals for quality with specific targets, and aimed to create a quality plan integral to their broader strategic agenda.[32, 37] Contrary findings however suggest that the Board rarely set the agenda for the discussion on quality,[37] did not provide the ideas for their strategies,[32] and were largely uninvolved in strategic planning for QI.[48] In the latter case, the non-clinical Board managers felt that they held "passive" roles in quality decisions. This is important considering evidence that connects the activity of setting the hospital quality agenda with

For peer review only - http://bmjopen.bmj.com/site/about/guidelines.xhtml

For peer review only

better performance in process of care and mortality.[38] Additionally, Boards that established goals in four areas of quality and publicly disseminated strategic goals and reported quality information were linked to high hospital performance.[35, 38, 50]

Culture-centred

Activities aimed at enhancing patient safety/QI culture emerged from several studies across organisational tiers.[44, 47, 48, 53] Board and senior management's activities included encouraging an organisational culture of QI on norms regarding interdepartmental/multi-disciplinary collaboration and advocating QI efforts to clinicians and fellow senior managers, providing powerful messages of safety commitment and influencing the organisation's patient safety mission.[47, 53] Managers at differing levels focused on cultivating a culture of clinical excellence and articulating the organisational culture to staff.[44] Factors to motivate/engage middle and senior management in QI included senior management commitment, provision of resources and managerial role accountability.[40, 46] Findings revealed connections between senior management and Board priorities and values with hospital performance and on middle management quality-related activities. Ensuring capacity for high quality standards also appears within the remit of management, as physician credentialing was identified as a Board managers' responsibility in more than one study.[38, 48] From this review it is unclear to what degree Board involvement in the credentialing process has a significant impact on quality.[38, 41]

Data-centred

Information on quality and safety are continually supplied to the Board.[51] At all levels of management, activities around quality and safety data or information were recognised in 6 studies.[35, 38, 43, 45, 47, 53] Activities included collecting and collating information,[43] reviewing quality information,[35, 38, 53] using measures such as incident reports and infection rates to forge changes,[53] using patient satisfaction surveys,[35] taking corrective

For peer review only - http://bmjopen.bmj.com/site/about/guidelines.xhtml

action based on adverse incidents or trends emphasised at Board meetings,[38] and providing feedback.[43, 47] The studies do not specify the changes made based on the data-related activities by senior managers; one study identified that frontline managers predominantly used data from an incident reporting tool to change policy/practice and training/education and communication between care providers.[45] However, overseeing data generally was found to be beneficial, as hospitals that carried out performance monitoring activities had significantly higher scores in process of care and lower mortality rates than hospitals that did not.[38]

Managerial impact on quality and safety outcomes

We have considered the associations found between specific managerial involvement and its affect on quality and safety. Here, we summarise the impact and importance of their general role. Of the articles that looked at either outcomes of management involvement in quality or at its perceived importance, 6 articles suggested that their role was beneficial to quality and safety performance.[32, 35, 38, 40, 49, 53] Senior management support and engagement was identified as one of the primary factors associated with good hospital-wide quality outcomes and QI programme success.[35, 38, 40, 49] Conversely, 6 articles suggest that managers' involvement (from the Board, middle and frontline) has little, no or a negative influence on quality and safety.[35, 38, 41, 42, 44, 49] Practices that showed no significant association with quality measures included Board's participation in physician credentialing.[35, 38] Another noted that if other champion leaders are present, management leadership was not deemed necessary.[44] Two articles identified a negative or inhibitory effect on evidence-based practices and staff productivity from frontline and middle managers.[41, 42]

For peer review only - http://bmjopen.bmj.com/site/about/guidelines.xhtml

For peer review only

Contextual factors related to managers' quality and safety role

Most of the articles focussed on issues that influenced the managers' role or their impact, as opposed to discussing the role of the managers. These provide an insight in to the types of conditions in which a manager can best undertake their role to affect quality and safety. Unfortunately it appears that many of these conditions are not in place.

Two studies found that a Board quality committee is a positive variable in quality performance, but that fewer than 60% had them.[38, 50] Similarly, compensation and performance evaluation linked to executive quality performance was identified in 4 articles [35, 37, 38, 49] and associated with better quality performance indicators,[38, 49] but quality measures were insufficiently included in CEOs' performance evaluation.[35, 37] The use of the right measures to drive QI was raised in relation to Board managerial engagement in quality [35] and to impact on patient care improvement,[51] yet, almost half of this sample did not formally adopt system-wide measures and standards for quality. To aid them in these tasks, evidence indicates the common use of QI measure tools, such as a dashboard or scorecard, [37, 49, 50] with promising associations between dashboard use and quality outcomes.[38, 50]

Other factors linked to quality outcomes include management-staff relationship/high interactions between the Board and medical staff when setting quality strategy,[49] and managerial expertise. Although a connection between knowledge with quality outcomes was not found,[38] high performing hospitals have shown higher self-perceived ability to influence care, expertise at the Board and participation in training programs that have a quality component.[50] Disappointingly, there is a low level of CEO knowledge on quality and safety reports,[35] possibly little Boardroom awareness on salient nursing quality issues,[36] and little practice identified to improve quality literacy for the Board.[32, 37] There is however promise for new managers through relevant training at induction and by recruitment of those with relevant expertise.[32]

For peer review only - http://bmjopen.bmj.com/site/about/guidelines.xhtml

The Quality Management IPO Model

The input process output (IPO) model is a conceptual framework that helps to structure the review findings in a useful way, please see Figure 2.[54, 55] This literature may be conceptualised by considering what factors contribute (input) to managerial activities (process) that impact on quality and safety (output). The three factors are interrelated and interchangeable, presented by the cyclical interconnecting diagram. This diagram enables a clearer mental picture of what a manager should consider for their role in quality and safety. Specifically, the input factors suggest certain organisational factors that should be put in place alongside individual factors to prepare for such a role (e.g. standardised quality measures, motivation, education and expertise, and a good relationship with clinicians). The processes present the strategy, culture and data-centred areas where managers (according to the literature) are and/or should be involved (e.g. driving improvement culture, goal-setting and providing feedback on corrective actions for adverse events). The outputs identify managerial influences that are positive, negative or have little or no established association with quality performance (e.g. positive outcomes of care, achieving objectives and engaging others in quality of care). This helps to identify areas where it is possible to make an impact through the processes mentioned. With further empirical studies on this topic, this model could be strengthened to become a more robust set of evidence-based criteria and outcomes.

For peer review only

First author; Year [Country]	Methods	Sample size [number of organisations]	Population sample [Level of management reported on (position of managers)]	Outcome Measure	Management Roles (Managerial quality and safety activities, time spent and engagement & key perceived importance and context factors)	Quality assessment score for qualitative studies	Quality assessment score for quantitative studies	Findings pertaining to research questions *[Time spent; Activities, Engagement; Impact (including perceived effectiveness); Contextual factors]*
Baker et al, 2010 [Canada][32]	Mixed methods (interviews, case studies, surveys)	n=15 interviews; n=4 Board case studies; n=79 surveys [79 organisations]	Managers [Board management]	Perceptions of managers on management Board practices in quality and safety	• Less than half (43%) of Boards reported that they addressed quality and patient safety issues in all meetings<br>• One-third of Boards spend 25% of their time or more on quality and patient safety issues<br>• More than 80% of Boards have formally established strategic goals for quality with specific targets, but a majority of Board chairs indicate that their Boards did not provide the ideas for strategic direction or initiatives<br>• Board chairs reported a low participation in education on quality and safety: 43% reported that all the Board members participated, 19% stated that more than half participated and 23% said it was less than a quarter of the Board<br>• Most Board chairs (87%) reported Board member induction training on responsibilities for quality and safety, although almost a third (30%) reported few or no opportunities for education on this, 42% reported some opportunities and 28% reported many)<br>• Approximately half (57%) of the Board chairs acknowledged recruitment of individuals that have knowledge, skills and experience in quality and patient safety onto the Board. A Board skills matrix included quality and safety as one of the competency areas<br>• Over half (55%) of board chairs rated their board's effectiveness in quality and safety oversight as very/extremely effective and 40% as somewhat effective.	16/20 (80%)	12/22 (55%)	Time Activities Impact Context
Balding 2005 [Australia] [46]	Mixed methods (action research, surveys & focus groups)	n=35 [1 hospital]	Managers [Middle management (Nursing managers and allied health managers)]	Self-reported perceptions of managers on their engagement in a QI programme	Five elements deemed essential to middle manager engagement:<br>• (1) Senior management commitment and leadership (e.g. senior management provides strategic direction for QI plan)<br>• (2) Provision of resources and opportunities for QI education and information dissemination (e.g. basic QI skills provided to all staff)<br>• (3) Senior and middle manager role accountability (e.g.: senior managers and middle managers agree QI roles and expectations)<br>• (4) Middle manager involvement in QI planning (e.g. senior and middle managers plan together)<br>• (5) Middle managers own and operate QI program (e.g. ongoing review and evaluation of the progress of the QI program by the middle and senior managers)	14/20 (70%)	15/22 (68%)	Activities Engagement Impact

For peer review only - http://bmjopen.bmj.com/site/about/guidelines.xhtml

Bradely et al, 2003 [US][47]	Qualitative (interviews)	n=45 [8 hospitals]	Clinical staff and senior management [Senior management (unspecified)]	Perceptions of roles and activities that comprise senior management's involvement in quality improvement efforts	Five common roles and activities that captured the variation in management involvement in quality improvement efforts:<br>• (1) Personal engagement of senior managers<br>• (2) Management's relationship with clinical staff<br>• (3) Promotion of an organisational culture of quality improvement<br>• (4) Support of quality improvement with organisational structures<br>• (5) Procurement of organisational resources for quality improvement efforts	19/20 (95%)	NA	Activities Engagement Impact
Bradely et al, 2006 [US][40]	Mixed-methods (surveys & interviews)	n=63 survey respondents [63 hospitals]; n=102 interviewees [13 hospitals]	Managers [Senior management (chief operating officer, vice president, medical director, chief nursing officer, director of volunteers, program director)]	Perceptions of management-related factors around the HELP programme	• Providing resources for needed staffing or staff training<br>• Promoting the program among the governing Board, physicians who were initially less involved, and other administrators<br>• Senior management support reported as the primary enabling factor in the implementation of such programmes (96.6%), along with a lack of support as the primary reason for not implementing the programme (65.0%)<br>• The interviews supported that having an administrative champion was considered essential to their program's success	19/20 (95%)	17/22 (77%)	Activities Engagement Impact Context
Braithwaite et al, 2004 [Australia] [52]	Mixed methods (ethnographic work, observations & focus groups)	n=64 managers in focus groups [1 hospital]; Ethnographic case studies and n=4 observed [2 hospitals]	Managers [Frontline management (Medical managers, nurse managers & allied health managers)]	Observations and self-reported perceptions of clinician-managers' activities	• Quality was the least discussed topic (e.g. Continuous quality improvement)<br>• The most discussed topic was people (e.g. staffing, delegating) and organisational issues, e.g. beds and, equipment	16/20 (80%)	NA	Time

For peer review only

Caine & Kenwrick, 1997[UK][41]	Qualitative (interviews)	n=10 [2 hospitals]	Managers [Middle management (Clinical directorate managers)]	Self-reported perceptions of managers on the managers' role in facilitating evidence-based practice in their nursing teams	• Managers saw their role in research implementation as a facilitator, ensuring quality and financial objectives and standards were met<br>• Managers perceived their facilitatory behaviours produced a low level of clinical change.<br>• Managers are not actively advocating research-based practice and failing to integrate it into everyday practice. Their behaviour inhibited the development of evidence-based nursing practice<br>• Devolved responsibility of use of research to individual professionals	14/20 (70%)	NA	Activities Impact
Fox, Fox & Wells 1999 [US][42]	Quantitative (surveys & self kept activity logs)	n=16 [1 hospital]	Managers [Frontline management (Nurse administrative managers (NAMs))]	Self-reported perceptions of managers on their activities impacting unit personnel productivity & monitored time/effort allocated to each function & managers' hours worked, patient admissions and length of stay.	• The small amount of total management allocated to QI (2.6%) was the least time spent of all management functions<br>• A negative relationship between time spent in QI activities and unit personnel productivity. An increase (from 2.5% to 5%) in QI time/effort by NAMs would reduce staff productivity significantly by approximately 8%.<br>• The greater the experiences of NAMs as managers, the more time spent on QI. These seasoned NAMs spent more time on monitoring, reporting QI results, and quality improvement teams (statistics nor provided)	NA	13/22 (59%)	Time Activities Impact (Objective outcome measure)
Harris, 2000 [UK][43]	Quantitative (surveys)	n=42 [42 hospitals]	Managers [Middle management (Nurse managers)]	Self-reported perceptions of managers on managers' quality and safety practices	• The majority of managers (91%) who received collated incident information used it to feed back to their own staff. 60% always fed back to staff, 28% sometimes did, 2% never did.<br>• Of the Trusts that had written guidance on types of clinical incident to report, 80% of managers had general guidance and fewer (20%) had written speciality specific guidance.<br>• 76% of managers reported information collation of clinical incidents. Of these, 59% were involved in data collection themselves	NA	13/22 (59%)	Activities

For peer review only - http://bmjopen.bmj.com/site/about/guidelines.xhtml

Jha and Epstein, 2010 [US][50]	Quantitative (surveys)	n=722 [767 hospitals]	Managers [Board]	Perceptions of managers on the role of managers in quality and safety & quality outcome measurement (from Hospital Quality Alliance) i.e. 19 practices for care in 3 clinical conditions	• Two-thirds (63%) of Boards had quality as an agenda item at every meeting<br>• Fewer than half (42%) of the hospitals spent at least 20% of the Board's time on clinical quality<br>• 72% of Boards regularly reviewed a quality dashboard<br>• Most respondents reported that their Boards had established, endorsed, or approved goals in four areas of quality: hospital-acquired infections (82%), medication errors (83%), the HQA/Joint commission core measures (72%), and patient satisfaction (91%)<br>High-performing hospitals were more likely than low-performing hospitals to have:<br>• Board reviews of a quality dashboard regularly (<0.001) & of clinical measures (all <0.05)<br>• Quality performance on the agenda at every Board meeting (0.003)<br>• At least 20% of Board time on clinical quality (0.001)<br>• Has a quality subcommittee (0.001)	NA	22/22 (100%)	Time Activities Impact (Objective outcome measure) Context
Jiang et al, 2008 [US][37]	Quantitative (surveys)	n=562 [387 hospitals]	Managers [Board & Senior management (presidents/ CEOs)]	Perceptions of managers on managers' practices in quality and safety; and outcomes of care (composite scores of risk-adjusted mortality indicators)	• 75% of CEOs reported that most to all of the Board meetings have a specific agenda item devoted to quality. Only 41% indicated that the Boards spend more than 20 percent of its meeting time on the specific item of quality.<br>The following activities were most reported to be performed:<br>• Board establishing strategic goals for QI (81.3%)<br>• Use quality dashboards to track performance (86%)<br>• Follow up corrective actions related to adverse events (83%)<br>The following activities were least reported to be performed:<br>• Board involvement in setting the agenda for the discussion on quality (42.4%)<br>• Inclusion of the quality measures in the CEO's performance evaluation (54.6%)<br>• Improvement of quality literacy of Board members (48.9%)<br>• Board written policy on quality and formally communicated it (30.8%)	NA	20/26 (77%)	Time Activities Impact (Objective outcome measure) Context
Jiang et al, 2009 [US][38]	Quantitative (surveys)	n=490 [490 hospitals]	Managers [Board & Senior management (CEOS & Hospital presidents reports)]	Perceptions of managers on manager's practices in quality and safety; and process of care measures (20 measures in 4 clinical areas); and outcome measures (composite scores of risk-adjusted	Board practices found to be associated with better performance (all P<0.05) in process of care (POC) and adjusted mortality (M) included:<br>• Having a Board quality committee (83.8%POC, 6.2M versus 80.2%POC, 7.9M without a committee)<br>• Establishing strategic goals for quality improvement (82.8%POC, 6.6M versus 80.3%POC, 7.9M)<br>• Being involved in setting the quality agenda for the hospital (83.2%POC, 6.4M versus 80.9%POC, 7.7M)<br>• Including a specific item on quality in Board meetings (83.2%POC, 6.5M versus 78.5%POC, 8.6M)<br>• Using a dashboard with national benchmarks and internal data that includes indicators for clinical quality, patient safety, and patient satisfaction (all above 80%POC & below 6.5M versus all below 80%POC and above 7M)<br>• Linking senior executives' performance evaluation to quality and patient safety indicators (83.1%POC, 6.6M versus 80.4%POC, 7.6M)<br>Practices that did NOT show significant association with the quality measures for process and mortality include:<br>• Reporting to the Board of any corrective action related to adverse events	NA	22/24 (92%)	Activities Impact (Objective outcome measure) Context

For peer review only - http://bmjopen.bmj.com/site/about/guidelines.xhtml

For peer review only

				mortality indicators)	(82.5%POC, 7.0M versus 81.8%POC, 6.6M)<br>• Board's participation in physician credentialing (82.8%POC, 6.9M versus 81.5%POC, 6.9M)<br>• Orientation for new Board members on quality(82.9%POC, 6.8M versus 81.7%POC, 7.0M)<br>• Education of Board members on quality issues (82.8%POC, 7.0M versus 81.9%POC, 6.9M)			
Joshi & Hines, 2006 [US][35]	Mixed-methods (surveys & interviews)	n=37 survey respondents; n=47 interviewees [30 hospitals]	Managers [Board & Senior management (CEOs, Board chairs)]	Perceptions of managers on managers' practices in quality and safety and appropriate care measure (ACM) and risk adjusted mortality.	• Board engagement in quality was reported as satisfactory (7.58 by CEOs and 8.10 by Chairs on a 1-10 scale where 10 indicates greatest satisfaction)<br>• Board engagement was positively associated with perceptions of the rate of progress in improvement (r=.44, p = .05), and marginally associated with ACM scores (r=.41, p=.07)<br>• Approximately one third of Board meetings are devoted to discussing quality issues (reported at 35% by CEOs and 27% by Chairs)<br>• Integrating Quality Planning and Strategic Planning was also rated as satisfactory (7.67 by CEOs and 8.85 by Chairs)<br>• Approximately two-thirds of respondents reported using patient satisfaction surveys (70% and 65% reported by CEOs and Chairs respectively)<br>• Low level of CEO expertise in QI, as reported by themselves (2.70) and by Board Chairs (3.35%) on a scale of 1-10 where 1 is low familiarity and 10 is high familiarity.	12/20 (60%)	16/20 (80%)	Time Activities Engagement Impact (Objective outcome measure) Context
Levey et al, 2007 [US][48]	Qualitative (Interviews)	n=96 [18 hospitals]	Managers [Board & Senior management (Hospital Board members, CEOs, Chief Medical Officers, Chief Quality Officers, medical staff leaders)]	Perceptions of managers on managers' role in quality and safety	• Few CEOs were willing to take the lead for transformation to a "culture of quality"<br>• Board members were largely uninvolved in strategic planning for QI<br>• In terms of the Board's quality functions, respondents largely agreed that physician credentialing was their critical responsibility<br>• Non-physicians reported that they felt relegated to "passive" roles in decisions on quality and seemed reluctant to assume leadership roles in the quality domain.<br>• Board meeting agendas maintained a focus on financial issues, although patient safety/care and QI were gaining prominence<br>• About half of the respondents said that quality was not sufficiently highlighted during meetings. Estimates of time devoted to quality and safety issues at Board meetings were between 10% to 35%	13/20 (65%)	NA	Time Activities Engagement Context
Mastal, Joshi & Shulke 2007 [US][36]	Qualitative (interviews and a focus group)	n=73 interviewees; 1 focus group [63 hospitals]	Managers [Board & Senior management (Board chairs, CEOs, Chief Nurse Officers)]	Perceptions of managers on managers' role in quality and safety	• Two chief nursing officers (CNOs) reported that nursing quality was never addressed at Board meetings.<br>• Few of the CNOs, CEOs, and Board chairs responded that issues are discussed more frequently, such as at every meeting.<br>• Quality and patient safety measures for nurses are not consistently addressed during all hospital Board meetings.<br>• Staffing concerns are the most frequent measure of nursing quality reported at the Board level	12/20 (60%)	NA	Time Context

For peer review only

Poniatowski, Stanley & Youngberg, 2005 [US][45]	Quantitative (surveys)	n=515 [16 academic medical centers]	Managers [Frontline management –unclear whether frontline or middle managers (Unit Nurse managers)]	Self-reported perceptions of managers on their practices with Patient Safety Net (PSN)	• Managers reviewed on average 65% of the PSN events reported<br>As a result of what was learned from PSN data, 162 managers detailed their changes made to:<br>• Policies and practices (59%)<br>• Training, education and communication between care providers (27%)<br>• Purchase of new equipment and supplies (8%)<br>• Staffing (6%)	N/A	10/20 (50%)	Activities
Prybil et al, 2010(US) [51]	Quantitative (surveys)	n=123 [712 hospitals]	Managers [Board & Senior management (CEOs & Boards)]	Perceptions of managers on their role in quality and safety	• Health system Boards spent 23% of their Board meeting time on quality and safety issues. They only spent slightly more on financial issues (25.2%) and strategic planning (27.2%)<br>• Almost all (96%) CEOs said that the Boards regularly received formal written reports on quality targets<br>• 88% of CEOs said that the Boards had assigned quality and safety oversight to a standing Board committee<br>• All but one (98.9%) of the CEOs stated that they have specific performance expectations and criteria related to quality and safety<br>• CEOs reported 59% of the Boards formally adopted system-wide measures and standards for quality	N/A	14/22 (64%)	Time Activities Context
Saint et al, 2010 [US][44]	Qualitative (interviews)	n=86 (interviewees) [14 hospitals]	Senior hospital staff and managers [Mixed levels (Nurse managers, chief physicians, Chairs of medicine, Chief of Staffs, hospital directors, CEOs & clinical non-managerial staff)]	Perceptions of managers on managers' practices in healthcare-associated infection (HAI)	• Although committed leadership by CEOs can be helpful, it was not always necessary, provided that other hospital leaders were committed to infection prevention<br>Behaviours of leaders who successfully implemented/facilitated practices to prevent HAI:<br>• Cultivated a culture of clinical excellence and kept their eye on improving patient care<br>• Developed a vision<br>• Articulated the organisational culture well and conveyed that to staff at all levels.<br>• Focused on overcoming barriers and dealing directly with resistant staff or process issues that impeded prevention of HAI<br>• Cultivated leadership skills and inspired the people they supervised (motivating and energising them to work towards the goal of preventing HAI)<br>• Thought strategically while acting locally; planned ahead and left few things to chance<br>• They did the politicking before issues arose for committee votes<br>• They leveraged personal prestige to move initiatives forward<br>• They worked well across disciplines	16/20 (80%)	NA	Activities Engagement Impact

For peer review only

Vaughn et al, 2006 (US)[49]	Quantitative (surveys)	n=413 [413 hospitals]	Managers [Board & Senior management (chief executives & senior quality executives; Board, executives, clinical leadership)]	Perceptions of managers on managers' role in QI & observed hospital Quality index outcomes (risk-adjusted measures of morbidity, mortality, and medical complications)	• 72% of hospital Boards spent one-quarter of their time or less on quality-of-care issues. About 5% of Boards spent more than half of their time on these issues<br>• A majority of respondents reported great influence from government and regulatory agencies (87%), consumers (72%) and accrediting bodies (74%) on quality priorities. Although 44% of respondents also noted that multiple government and regulatory requirements were unhelpful<br>Better quality index scores (QIS) are associated with hospitals where the Board:<br>• Spends more than 25% of their time on quality issues (QIS 83 – QIS mean 100 across hospitals)<br>• Receives a formal quality performance measurement report (QIS 302)<br>• Bases the senior executives' compensation in part on QI performance (QIS 239)<br>• Engages in a great amount of interaction with the medical staff on quality strategy	NA	21/22 (95%)	Time<br>Activities<br>Engagement<br>Impact (Objective outcome measure)<br>Context
Weingart & Page, 2004 [US][53]	Qualitative (case study documentation analysis and meeting discussions and focus group)	n=30 [10 hospitals and other stakeholder organisations]	Managers [Senior management (Executives)]	Perceptions of managers on manager's practices in quality and safety	Executives developed and tested a set of governance best practices in patient safety, such as:<br>• Creation of a Board committee with explicit responsibility for patient safety<br>• Development of Board level safety reports, introduction of educational activities for Board members<br>• Participation of Board members in executive walk rounds.<br>• Executives reviewed measures to assess safety (e.g. incident reports, infection rates, pharmacist interventions, readmissions, etc)<br>• Executives endorsed a statement of public commitment to patient safety.<br>• Executives believed their behaviours affected their organisations' patient safety mission	14/20 (70%)	NA	Activities<br>Impact

Table 1 Table of characteristics and summary findings of included studies

----

Enter Figure 2

For peer review only - http://bmjopen.bmj.com/site/about/guidelines.xhtml

DISCUSSION

Our review examined the role of managers in maintaining and promoting safe, quality care. The existing studies detail the time spent, activities and engagement of hospital managers and Boards, and suggest that these can positively influence quality and safety performance. They further reveal that such involvement is often absent, as are certain conditions that may help them in their work.

Evidence from the review promotes hospitals to have a Board quality committee, with a specific item on quality at the Board meeting, a quality performance measurement report and a dashboard with national quality and safety benchmarks along with standardised quality and safety measures. Outside of the Boardroom, the implications are for senior managers to build a good infrastructure for staff-manager interactions on quality strategies and attach compensation and performance evaluation to quality and safety achievements. For QI programmes, managers should keep in mind its consistency with the hospital's mission and provide commitment, resources, education, and role accountability. Literature elsewhere supports much of these findings, such as the use of quality measurement tools [21, 56] better quality-associated compensation, a separate quality committee,[16, 57] and has also emphasised poor manager-clinician relationships as damaging to patients and QI.[58, 59]

Some of the variables that were shown to be associated with good quality performance, such as having a Board committee, compensation/performance and adoption of system-wide measures, were lacking within the study hospitals. There are also indications of the need to develop Board and senior managerial knowledge and training on quality and safety. Furthermore, this review indicates that many managers do not spend sufficient time on quality and safety. The included studies suggest time spent by the Board should exceed 20-25%, yet the findings expose that certain Boards devote less time than this. Inadequacies of time allocated to quality at the Board meeting hold concerning implications for quality. If little

For peer review only - http://bmjopen.bmj.com/site/about/guidelines.xhtml

time is taken to consider quality of care matters at the highest level, an inference is that less attention will be paid to prevention and improvement of quality within the hospital. While the position that the item appears on the agenda is deemed of high importance, it is unimportant if the duration on this item is overly brief. In this vein, the inadequate time on quality spent by some may reflect their prioritisation on quality in relation to other matters discussed at the meetings or the value perceived to be gained from discussing it further. It might instead however be indicative of the difficulties in measuring time spent on quality by management. Some of these studies provide us not necessarily with Board managers' time on quality and safety but their time spent on this at Board meetings. The two may not equate and time spent on quality may not necessarily be well-spent.[36] The emerging inference that managers greatly prioritise other work over quality and safety is not explicit, with further research required to identify what time is actually devoted and required from managers inside and outside of the Boardroom. Perhaps encouragingly, the more recent studies present more time spent on quality and safety than the earlier studies. Yet even the most recent empirical studies not included in our review conclude that much improvement is required.[60]

This review presents a wide-range of managerial activities, such as public reporting of quality strategies and driving an improvement culture. It further highlights the activities that appear to affect quality performance. Priorities for Boards/managers are to engage in quality, establish goals and strategy to improve care, and get involved in setting the quality agenda, support and promote a safety and QI culture, cultivate leaders, manage resisters, plan ahead and procure organisational resources for quality. Again, much of the findings support the assertions made in the non-empirical literature. Above all, involvement through action, engagement and commitment have been suggested to positively affect quality and safety.[61] While researchers have stressed the limited empirical evidence showing conclusive connection between management commitment and quality,[21] some supporting evidence however can be unearthed in research that concentrates on organisational factors

For peer review only

related to changes made to improve quality and safety in healthcare.[62-64] In addition to this evidence, a few studies have specifically investigated the impact that hospital managers have on quality and safety (rather than examination of their role). These studies have shown senior managerial leadership to be associated with a higher degree of QI implementation,[65] promotion of clinical involvement,[66, 67] safety climate attitudes,[68] and increased Board leadership for quality.[57] A clear case for the positive influence of management involvement with quality is emerging both from the findings of our review and related literature.

There is a dearth of empirical research on the role of hospital managers in quality of care and patient safety and quality improvement. This evidence is further weakened by the largely descriptive nature of many of the studies. They most lack theoretical underpinnings and appropriate objective measures. Very few studies reported objective clinical quality outcome measures that better show the influence of managerial actions. Moreover, the content of many of the articles was dominated by the contextual issues surrounding managers' roles, rather than actual manager practices. Some of the outlined managerial actions would further benefit from more detail, for example, the literature fails to present changes made based on data-related activities at the Board or senior management level. Only one study clearly demonstrated that senior management and Board priorities can impact upon middle management quality-related activities and engagement. Considering the likely influence that seniors have on their managers, examination of the interactions between the different roles held (e.g. Boards setting policies on quality and middle managers implementing them) would improve our understanding of how these differences reflect in their time spent and actions undertaken. Supplementary work could also resolve contradictions that were found within the review, clarifying for example the positive impact of managerial expertise versus knowledge on quality and who sets the Board agenda for the discussion on quality. Research on this area is particularly required to examine middle and frontline managers, to take into consideration non-managers' perceptions, and to assess senior managers' time

For peer review only - http://bmjopen.bmj.com/site/about/guidelines.xhtml

and tasks outside of the Boardroom. Future studies would benefit from better experimental controls, ideally with more than one time point, verifications and reflections on qualitative work, robust statistical analysis, appropriate study controls, consideration of confounding variables, and transparent reporting of population samples, methodologies, and analyses used. Box 2 presents the key messages from this review.

Review limitations

There are several limitations of the present review pertaining to the search strategy and review process, the limited sample of studies, publication bias, and limitations of the studies themselves. Specifically, the small number of included studies and their varied study aims, design and population samples make generalisations difficult. Grouped demographics, such as middle management, are justified by the overlap between positions. With more literature on this topic, distinctions could be made between job positions. Furthermore, more research on lower levels of management would have provided a better balanced review of hospital managers work and contributions to quality. Restricting the language of studies to English in the search strategy is likely to have biased the findings and misrepresent which countries conduct studies on this topic. There is an over-reliance on perceptions across the studies, which ultimately reduces the validity of the conclusions drawn from their findings. As most of the study findings relied on self-reports, social desirability may have resulted in exaggerated processes and inflated outputs. Although, encouragingly, one of the included studies found that managers that perceived their Boards to be effective in quality oversight were from hospitals that had higher processes-of-care scores and lower risk adjusted mortality. The quality assessment scores should be viewed with caution; such scores are subjective and may not take into consideration factors beyond the quality assessment scale used. Due to the enormity of this review, the publication of this article is some time after the search run date. As there is little evidence published on this topic, we consider this not to greatly impact on the current relevance of the review, particularly as the literature reviewed spans almost

For peer review only

three decades. However, we acknowledge the need for an update of the data as a limitation of this review.

Conclusion

The modest literature that exists suggests that managers' time spent, engagement and work can influence quality and safety clinical outcomes, processes and performance. Managerial activities that affect quality performance are especially highlighted by this review, such as establishing goals and strategy to improve care, setting the quality agenda, engaging in quality, promoting a quality improvement culture, managing resisters, and procurement of organisational resources for quality. Positive actions to consider include the establishment of a Board quality committee with a specific item on quality at the Board meeting, a quality performance measurement report and a dashboard with national quality and safety benchmarks, performance evaluation attached to quality and safety, and an infrastructure for staff-manager interactions on quality strategies. However, many of these arrangements were not in place within the study samples. There are also indications of a need for managers to devote more time to quality and safety. More than one study suggest time spent by the Board should exceed 20-25%, yet the findings expose that certain Boards devote less time than this. Much of the content of the articles focused on such contextual factors rather than on the managerial role itself; more empirical research is required to elucidate managers' actual activities. Research is additionally required to examine middle and frontline managers, non-manager perceptions, and to assess senior managers' time and tasks outside of the Boardroom. We present the IPO model to summarise the evidence-based promotion of conditions and activities in order to guide managers on the approaches taken to influence quality performance. More robust empirical research with objective outcome measures could strengthen this guidance.

For peer review only

- There is a dearth of empirical evidence on hospital managerial work and its influence on quality of care.
- There is some evidence that Boards'/managers' time spent, engagement and work can influence quality and safety clinical outcomes, processes and performance.
- Some variables associated with good quality performance were lacking in study hospitals.
- Many Board managers do not spend sufficient time on quality and safety.
- There is a greater focus on the contextual issues surrounding managers' roles than on examining managerial activities.
- Research is required to examine middle and frontline managers, to take into consideration non-managers' perceptions, and to assess senior managers' time and tasks outside of the Boardroom. More robust methodologies with objective outcome measures would strengthen the evidence.
- We present a model to summarise the evidence-based promotion of conditions and activities for managers to best affect quality performance.

Box 2 Key messages from the systematic literature review

For peer review only - http://bmjopen.bmj.com/site/about/guidelines.xhtml

ACKNOWLEDGEMENTS

We would like to thank Miss Dina Grishin for helping to review the abstracts and Miss Ana Wheelock for helping to assess the quality of the articles.

FUNDING

This work was supported by funding from the Health Foundation and the National Institute for Health Research.

CONTRIBUTORS

All co-authors contributed to the study design and review of drafts of the article. The first author screened all of the articles for inclusion in this review and appraised the study quality. Dr Anna Renz and Miss Dina Grishin screened a sample of these at title/abstract and full text, and Miss Ana Wheelock scored the quality of a sample of the included articles.

COMPETING INTERESTS

There are no competing interests.

DATA SHARING STATEMENT

The extraction table of the included studies and individual study quality scores can be made available on request to the corresponding author, Dr Anam Parand, at a.parand@imperial.ac.uk.

For peer review only

REFERENCES

1. Kizer KW. Patient Safety: A Call to Action: Safety Is a Critical Component of Quality. Medscape General Medicine 2001;**3:10**.

2. Kizer KW. Establishing health care performance standards in an era of consumerism. Journal of the American Medical Association 2001;**286**:1213-7.

3. Berwick D. Will is the way to win the patient safety war. Health Serv J 2007;**117**:18-9.

4. Institute of Medicine. Crossing the quality chasm: a new health system for the 21st century. Washington: National Academy Press; 2001.

5. Gosfield AG, Reinertsen JL. The 100,000 lives campaign: crystallizing standards of care for hospitals. Health aff 2005;**24**:1560–70.

6. Denham CR. Is your hospital as safe as your bank?...Time to ask your board. J Patient Saf 2009;**5**:122-6.

7. Francis R. Report of the Mid Staffordshire NHS Foundation Trust Public Inquiry London: The Stationery Office 2013 [accessed on 18.03.13]. Available from: http://www.midstaffspublicinquiry.com/report.

8. National Advisory Group on the Safety of Patients in England. A promise to learn – a commitment to act: Improving the Safety of Patients in England 2013 [accessed on 15.08.13]. Available from: https://www.gov.uk/government/uploads/system/uploads/attachment_data/file/226703/Berwick_Report.pdf.

9. O'Dea A, Flin R. Site managers and safety leadership in the offshore oil and gas industry. Safety Sci 2001;**37**:39-57.

10. Zohar D, Luria G. The use of supervisory practices as leverage to improve safety behavior: a cross-level intervention model. Journal of Safety Res 2003;**34**:567-77.

11. Flin R. "Danger-Men at Work": Management Influence on Safety. Hum Factor and Ergon Man 2003;**13**:261-8.

12. Guidotti TL. How can managers visibly promote safety and health protection? J Occ Env Med 2010;**52**:1141-2.

13. Conway J. Getting boards on board: engaging governing boards in quality and safety. Jt Comm J Qual Patient Saf 2008;**34**:214-20.

14. Gautam KS. A call for board leadership on quality in hospitals. Qual Manag Health Care 2005;**14**:18-30.

15. Caldwell C, Butler G, Grah J. Breakthrough quality: what the board must do. Trustee 2008;**61**:32-3, 1.

16. Goeschel CA, Wachter RM, Pronovost PJ. Responsibility for quality improvement and patient safety: hospital board and medical staff leadership challenges. Chest 2010;**138**:171-8.

17. McGinn P, Dave R. What is the board's role in quality? Trustee 2007;**60**:40.

18. Øvretveit J. Leading improvement effectively: Review of research 2009 [accessed on 21.12.12]. Available from: http://www.health.org.uk/public/cms/75/76/313/560/Leading%20imrpovement%20effectively.pdf?realName=YUaqJk.pdf.

19. Flin R, Yule S. Leadership for safety: industrial experience. Qual and Saf Health Care 2004;**13**:45-51.

For peer review only

20. West E. Management matters: the link between hospital organisation and quality of patient care. Qual and Saf Health Care 2001;**10**:40-8.

21. Glickman SW, Baggett KA, Krubert CG, et al. Promoting quality: the health-care organization from a management perspective. Int J Qual Health Care 2007;**19**:341-8.

22. Gaucher E, Kratochwill EW. The leader's role in implementing total quality management. Qual Manag Health Care 1993;**1**:10-8.

23. Thorman KE, Capitulo KL, Dubow J, et al. Perinatal patient safety from the perspective of nurse executives: a round table discussion. JOGNN 2006;**35** 409-16.

24. Aspden P, Corrigan JM, Wolcott J, Erickson SM. Patient safety: achieving a new standard of care. Institute of Medicine, editor. Washington: National Academies Press; 2004.

25. AHRQ. AHRQ Patient Safety Network Glossary 2011 [accessed on 19.05.12]. Available from: http://www.psnet.ahrq.gov/glossary.aspx?indexLetter=P.

26. Merali F. NHS managers' commitment to a socially responsible role: the NHS managers' views of their core values and their public image. Social Responsibility Journal 2005;**1**:38-46.

27. Mintzberg H. The Nature of Managerial Work. New York: Harper & Row; 1973.

28. Tanon AA, Champagne F, Contandriopoulos AP, Pomey MP, Vadeboncoeur A, Nguyen H. Patient safety and systematic reviews: finding papers indexed in MEDLINE, EMBASE and CINAHL. Quality saf health care 2010;**19**:452-61.

29. Cohen J. A coefficient for agreement for nominal scales. Educ Psych Meas 1960;**20**:37–46.

30. Cicchetti DV, Feinstein AR. High agreement but low kappa: II. Resolving the paradoxes. J Clin Epidemiol 1990;**43**:551-8.

31. Viera AJ, Garrett JM. Understanding interobserver agreement: the kappa statistic. Family med 2005;**37**:360-3.

32. Baker GR, Denis JL, Pomey MP, et al. Effective governance for quality and patient safety in Canadian Healthcare organizations: a report to the Canadian Health Services Research Foundation and the Canadian Patient Safety Institute. Ottawa: Canadian Health Services Research Foundation and Canadian Patient Safety Institute, 2010.

33. Moher D, Liberati A, Tetzlaff J, Altman DG. Preferred reporting items for systematic reviews and meta-analyses: the PRISMA statement. Ann Intern Med 2009;**151**:264-9.

34. Kmet LM, Lee RC, Cook LS. Standard Quality Assessment Criteria for Evaluating Primary Research Papers from a Variety of Fields. Edmonton: Alberta Heritage Foundation for Medical Research, 2004.

35. Joshi MS, Hines SC. Getting the board on board: Engaging hospital boards in quality and patient safety. Jt Comm J Qual Patient Saf 2006;**32**:179-87.

36. Mastal MF, Joshi M, Schulke K. Nursing leadership: championing quality and patient safety in the boardroom. Nurs Econ 2007;**25**:323-30.

37. Jiang HJ, Lockee C, Bass K, et al. Board engagement in quality: findings of a survey of hospital and system leaders. J Healthc Manag 2008;**53**:121-34.

38. Jiang HJ, Lockee C, Bass K, et al. Board oversight of quality: Any differences in process of care and mortality? J Healthc Manag 2009;**54**:15-29.

39. Walsh D, Downe S. Meta-synthesis method for qualitative research: a literature review. J Adv Nurs 2005;**50**:204-11.

40. Bradley EH, Webster TR, Schlesinger M, et al. The roles of senior management in improving hospital experiences for frail older adults. J Healthc Manag 2006;**51**:323-36.

For peer review only

41. Caine C, Kenrick M. The role of clinical directorate managers in facilitating evidence-based practice: a report of an exploratory study. J Nurs Manag 1997;**5**:157-65.

42. Fox RT, Fox DH, Wells PJ. Performance of first-line management functions on productivity of hospital unit personnel. J Nurs Adm 1999;**29**:12-8.

43. Harris A. Risk management in practice: how are we managing? Clin Perform Qual Health Care 2000;**8**:142-9.

44. Saint S, Kowalski CP, Banaszak-Holl J, et al. The importance of leadership in preventing healthcare-associated infection: Results of a multisite qualitative study. Infect Control Hosp Epidemiol 2010;**31**:901-7.

45. Poniatowski L, Stanley S, Youngberg B. Using information to empower nurse managers to become champions for patient safety. Nurs Adm Q 2005;**29**:72-7.

46. Balding C. Embedding organisational quality improvement through middle manager ownership. Int J Health Care Qual Assur 2005;**18**:271-88.

47. Bradley EH, Holmboe ES, Mattera JA, et al. The roles of senior management in quality improvement efforts: what are the key components? J Healthc Manag 2003;**48**:15-28.

48. Levey S, Vaughn T, Koepke M, et al. Hospital Leadership and Quality Improvement: Rhetoric Versus Reality. J Patient Saf 2007;**3**:9-15.

49. Vaughn T, Koepke M, Kroch E, et al. Engagement of Leadership in Quality Improvement Initiatives: Executive Quality Improvement Survey Results. J Patient Saf 2006;**2**:2-9.

50. Jha A, Epstein A. Hospital governance and the quality of care: Health Aff 2010;29:182-187.

51. Prybil LD, Peterson R, Brezinski P, et al. Board oversight of patient care quality in community health systems. Am J Med Qual 2010;**25**:34-41.

52. Braithwaite J, Finnegan TP, Graham EM, et al. How important are quality and safety for clinician managers? Evidence from triangulated studies. Clin Governance 2004;**9**:34-41.

53. Weingart SN, Page D. Implications for practice: challenges for healthcare leaders in fostering patient safety. Qual Saf Health Care 2004;**13**:52-6.

54. Flin R, Maran N. Identifying and training non-technical skills for teams in acute medicine. Qual Saf Health Care 2004;**13**:80-4.

55. Kunzle B, Kolbe M, Grote G. Ensuring patient safety through effective leadership behaviour: A literature review. Safety Sci 2010;**48**:1-17.

56. Naranjo-Gil D. Strategic performance in hospitals: the use of the balanced scorecard by nurse managers. Health Care Manage Rev 2009;**34**:161-70.

57. Weiner BJ, Alexander JA, Shortell SM. Leadership for quality improvement in health care; empirical evidence on hospital boards, managers, and physicians. Med Care Res Rev 1996;**53**:397-416.

58. Edwards N, Marshall M, McLellan A, et al. Doctors and managers: A problem without a solution? BMJ 2003;**326**:609-10.

59. Wilkinson JE, Powell A, Davies H. Are clinicians engaged in quality improvement? A review of the literature on healthcare professionals' views on quality improvement initiative: Health Foundation Report; 2011 [accessed on 21.12.12]. Available from: http://www.health.org.uk/public/cms/75/76/313/2440/Are%20clinicians%20engaged%20in%20quality%20improvement.pdf?realName=y8NfS4.pdf.

60. Jha AK, Epstein AM. A Survey Of Board Chairs Of English Hospitals Shows Greater Attention To Quality Of Care Than Among Their US Counterparts. Health Aff 2013;**32**:677-85.

For peer review only

61. Stanton P. The role of an NHS Board in assuring the quality of clinically governed care and the duty of trust to patients. Clin Governance 2006;**11**:39-49.

62. Lukas CV, Holmes SK, Cohen AB, et al. Transformational change in health care systems: an organizational model. Health Care Manage Rev 2007;**32**:309-20.

63. Shortell SM, Bennett CL, Byck GR. Assessing the impact of continuous quality improvement on clinical practice: what it will take to accelerate progress. The Milbank Q 1998;**76**:593-624, 510.

64. Keroack MA, Youngberg BJ, Cerese JL, et al. Organizational factors associated with high performance in quality and safety in academic medical centers. Acad Med 2007;**82**:1178-86.

65. Parker VA, Wubbenhorst WH, Young GJ, et al. Implementing quality improvement in hospitals: the role of leadership and culture. Am J Medical Qual 1999;**14**:64-9.

66. Weiner B, Shortell S, Alexander J. Promoting clinical involvement in hospital quality improvement efforts: the effects on top management, board, and physician leadership. Health Serv Res 1997;**32**:491-510.

67. Duckers MLA, Stegeman I, Spreeuwenberg P, et al. Consensus on the leadership of hospital CEOs and its impact on the participation of physicians in improvement projects. Health Policy 2009;**91**:306-13.

68. Vogus TJ, Sutcliffe KM. The impact of safety organizing, trusted leadership, and care pathways on reported medication errors in hospital nursing units. Med Care 2007;**45**:997-1002.

Figure Legends

Figure 1 Review stages based on PRISMA Flow Diagram[33]
Figure 2 The Quality Management IPO model

For peer review only

The Role of Hospital Managers in Quality and Patient Safety. A Systematic Review

For peer review only

Anam Parand[1], Sue Dopson[2], Anna Renz[1], Charles Vincent[3]

[1]Department of Surgery & Cancer, Imperial College London, London, UK
[2]Said Business School, University of Oxford, Oxford, UK
[3]Department of Experimental Psychology, University of Oxford, Oxford, UK

Correspondence to: Anam Parand, Department of Surgery & Cancer, Imperial College London, 503, 5th Floor Wright Fleming Building, St Mary's Campus, Norfolk Place, London W2 1PG, UK.
Email: a.parand@imperial.ac.uk; Tel: +44 (0) 207594 9726; Fax: +44 (0)20 7594 9726

Key Words: Quality of health care; Patient safety; Systematic literature review; Leadership, Management.

Abstract Word Count: 236 words

Manuscript Word Count: 5,3~~1~~29 words (excluding title page, abstract, references, acknowledgements and tables).

For peer review only - http://bmjopen.bmj.com/site/about/guidelines.xhtml

ABSTRACT

Objectives: To review the empirical literature to identify the activities, time spent and engagement of hospital managers in quality of care.

Design: A systematic review of the literature.

Methods: A search was carried out on the databases MEDLINE, PSYCHINFO, EMBASE, HMIC. The search strategy covered three facets: management, quality of care, and the hospital setting comprising medical subject headings and key terms. Reviewers screened 15,447 titles/abstracts and 423 full texts were checked against inclusion criteria. Data extraction and quality assessment were performed on 19 included articles.

Results: The majority of studies were set in the US and investigated Board/senior level management. The most common research designs were interviews and surveys on the perceptions of managerial quality and safety practices. Managerial activities comprised strategy, culture and data-centred activities, such as driving improvement culture and promotion of quality, strategy/goal-setting and providing feedback. Significant positive associations with quality included compensation attached to quality, using quality improvement measures and having a Board quality committee. However there is an inconsistency and inadequate employment of these conditions and actions across the sample hospitals.

Conclusion: There is some evidence that managers' time spent and work can influence quality and safety clinical outcomes, processes and performance. However, there is a dearth of empirical studies, further weakened by a lack of objective outcome measures and little examination of actual actions undertaken. We present a model to summarise the conditions and activities that affect quality performance.

For peer review only - http://bmjopen.bmj.com/site/about/guidelines.xhtml

ARTICLE SUMMARY

Strengths and limitations of this study

- This is the first systematic review of the literature that has considered the evidence on Boards' and managers' time spent, engagement and work within the context of quality and safety. This review adds to the widely anecdotal and commentary pieces that speculate on what managers should be doing by presenting what they are actually doing.
- The review reveals conditions and actions conducive to good quality management and offers a model to transparently present these to managers considering their own part in quality and safety.
- The search for this review has screened a vast amount of the literature (over 15,000 articles) across a number of databases.
- The small number of included studies and their varied study aims, design and population samples make generalisations difficult. With more literature on this topic, distinctions could be made between job positions.
- The quality assessment scores are subjective and may not take into consideration factors beyond the quality assessment scale used.

For peer review only

For peer review only - http://bmjopen.bmj.com/site/about/guidelines.xhtml

INTRODUCTION

Managers in healthcare have a legal and moral obligation to ensure a high quality of patient care and to strive to improve care. These managers are in a prime position to mandate policy, systems, procedures and organisational climates. Accordingly, many have argued that it is evident that healthcare managers possess an important and obvious role in quality of care and patient safety and that it is one of the highest priorities of healthcare managers.[1-3] In line with this, there have been calls for Boards to take responsibility for quality and safety outcomes.[4, 5] One article warned hospital leaders of the dangers of following in the path of bankers falling into recession, constrained by their lack of risk awareness and reluctance to take responsibility.[6] To add to the momentum are some high profile publicity of hospital management failures affecting quality and safety, eliciting strong instruction for managerial leadership for quality at the national level in some countries.[7, 8]

Beyond healthcare, there is clear evidence of managerial impact on workplace safety.[9-12] Within the literature on healthcare, there are non-empirical articles providing propositions and descriptions on managerial attitudes and efforts to improve safety and quality. This literature, made up of opinion articles, editorials and single participant experiences, present an array of insightful suggestions and recommendations for actions that hospital managers should take to improve the quality of patient care delivery in their organisation.[13-17] However, researchers have indicated that there is a limited evidence-base on this topic.[18-21] Others highlight the literature focus on the difficulties of the managers' role and the negative results of poor leadership on quality improvement (QI) rather than considering actions that managers presently undertake on quality and safety.[22, 23] Consequently, little is known about what healthcare managers are doing in practice to ensure and improve quality of care and patient safety, how much time they spend on this, and what research-based guidance is available for managers in order for them to decide on appropriate areas to become involved. Due perhaps to the broad nature of the topic, scientific studies exploring

For peer review only - http://bmjopen.bmj.com/site/about/guidelines.xhtml

these acts and their impact are likely to be a methodological challenge, although a systematic review of the evidence on this subject is notably absent. This present systematic literature review aims to identify empirical studies pertaining to the role of hospital managers in quality of care and patient safety. We define 'role' to comprise of managerial activities, time spent and active engagement in quality and safety and its improvement. Whilst the primary research question is on the managers' role, we take into consideration the contextual factors surrounding this role and its impact or importance as highlighted by the included studies. Our overarching question is 'What is the role of hospital managers in quality and safety and its improvement?' The specific review research questions are as follows:

- How much time is spent by hospital managers on quality and safety and its improvement?
- What are the managerial activities that relate to quality and safety and its improvement?
- How are managers engaged in quality and safety and its improvement?
- What impact do managers have on quality and safety and its improvement?
- How do contextual factors influence the managers' role and impact on quality and safety and its improvement?

METHODS

Concepts and definitions

Quality of care and patient safety were defined on the basis of widely accepted definitions from the Institute of Medicine (IOM) and the Agency for Healthcare Research and Quality Patient Safety Network (ARQH PSN). IOM define quality in healthcare as possessing the

For peer review only - http://bmjopen.bmj.com/site/about/guidelines.xhtml

following dimensions: safe, effective, patient-centred, timely, efficient and equitable.[4] They define patient safety simply as *"the prevention of harm to patients",*[24] and AHRQ define it is *"freedom from accidental or preventable injuries produced by medical care."*[25] Literature was searched for all key terms associated with quality and patient safety to produce an all-encompassing approach. A manager was defined as an employee that has subordinates, oversees staff, is responsible for staff recruitment and training, and holds budgetary accountabilities. Therefore, all levels of managers including Boards of managers were included in this review with the exception of clinical frontline employees, e.g. doctors or nurses, who may have taken on further managerial responsibilities alongside their work but do not have a primary official role as a manager. Those that have specifically taken on a role for quality of care, e.g. the modern matron, were also excluded. Distinction between senior, middle and frontline management were as follows: senior management hold Trust-wide responsibilities;[26] middle managers are in the middle of the organisational hierarchy chart and have one or more managers reporting to them;[27] frontline managers are defined as managers at the first level of the organisational hierarchy chart who have frontline employees reporting to them. Board managers include all members of the Board. Although, there are overlaps between senior managers and Boards (for example Chief Executive Officers (CEOs) may sit on hospital Boards), we aim to present senior and Board level managers separately due to the differences in their responsibilities and position. Only managers that would manage within or govern hospitals were included, with the exclusion of settings that solely served mental health or that comprised solely of non-acute care community services (in order to keep the sample more homogenous). The definition of 'Role' focused on actual engagement, time spent and activities that do or did occur rather than those recommended that should or could occur.

For peer review only - http://bmjopen.bmj.com/site/about/guidelines.xhtml

Search Strategy

Literature was reviewed between 01 Jan 1983 and 01 Nov 2010. Eligible articles were those that described or tested managerial roles pertaining to quality and safety in the hospital setting. Part of the search strategy was based on guidance by Tanon et al (2010).[28] EMBASE, MEDLINE, Health Management Information Consortium (HMIC) and PSYCHINFO databases were searched. The search strategy involved three facets (i.Management; ii.Quality; iii.Hospital Setting) and five steps. A facet (i.e. a conceptual grouping of related search terms) for role was not included in the search strategy, as it would have significantly reduced the sensitivity of the search.

Multiple iterations and combinations of all search terms were tested to achieve the best level of specificity and sensitivity. In addition to the key terms, Medical Subject Headings (MeSH®) terms were used, which were 'exploded' to include all MeSH subheadings. All databases required slightly different MeSH terms (named Emtree in Embase), therefore four variations of the search strategies were used (see online Appendix 1 for the search strategies). Additional limits placed on the search strategy restricted study subjects to human and the language to English. The search strategy identified 15,447 articles after duplicates had been removed.

Screening

Three reviewers (AP, AR and DG) independently screened the titles and abstracts of the articles for studies that fit the inclusion criteria. One reviewer (AP) screened all 15,447 articles, while two additional reviewers screened 30% of the total sample retrieved from the search strategy: AR screened 20% and DG screened 10%. On testing inter-rater reliability, Cohen's kappa correlations showed low agreement between AR and AP ($K=0.157$, $P<0.01$) and between DG and AP ($K=0.137$, $P<0.00$).[29] However, there was a high percentage of agreement between raters (95% and 89% respectively), which reveals a good inter-rater

For peer review only - http://bmjopen.bmj.com/site/about/guidelines.xhtml

For peer review only

reliability.[30, 31] Discrepancies were resolved by discussion and consensus. The main inclusion criteria were that: the setting was hospitals; the population sample reported on were managers; the context was quality and safety; the aim was to identify the managerial activities/time/engagement in quality and safety. The full inclusion/exclusion criteria and screening tool can be accessed in the online Appendices 2-3. Figure 1 presents the numbers of articles included and excluded at each stage of the review process.

Four hundred and twenty-three articles remained for full text screening. One reviewer (AP) screened all articles and a second reviewer (AR) reviewed 7% of these. A moderate agreement inter-rater reliability score was calculated ($K=0.615, P<0.001$) with 73% agreement. The primary reoccurring difference in agreement was regarding whether the article pertained to quality of care, owing to the broad nature of the definition. Each article was discussed individually until a consensus was reached on whether to include or exclude. Hand searching and cross-referencing were carried out in case articles were missed by the search strategy or from restriction of databases. One additional article was identified from hand searching,[32] totalling 19 articles included in the systematic review.

Enter Figure 1

Data extraction & methodological quality

The characteristics and summary findings of the 19 included studies are presented in Table 1. This Table is a simplified version of a standardised template that was used to ensure consistency in data extracted from each article. Each study was assessed using a quality appraisal tool developed by Kmet et al (2004),[34] which comprised of two checklists (qualitative and quantitative). Random included articles (32%) were scored by AW for

For peer review only

scoring consistency. All articles were scored on up to 24 questions with a score between 0-2; Box 1 shows an example definition of what constitutes 'Yes' (2), 'Partial' (1) and 'No' (0) rating criteria. The total percentage scores for each study are presented in Table 1. All studies were included regardless of their quality scores. Some cumulative evidence bias may results from two larger datasets split into more than one study each.[35-38] Through a narrative synthesis, we aimed to maintain the original meanings, interpretations and raw data offered by the articles.[39]

Rating	Criteria to verify whether question or objective is sufficiently described
**Yes**	Is easily identified in the introductory section (or first paragraph of methods section). Specifies (where applicable, depending on study design) *all* of the following: purpose, subjects/target population, and the *specific* intervention(s) /association(s)/descriptive parameter(s) under investigation. A study purpose that only becomes apparent after studying other parts of the paper is *not* considered sufficiently described.
**Partial**	Vaguely/incompletely reported (e.g. "describe the effect of" or "examine the role of" or "assess opinion on many issues" or "explore the general attitudes"...); *or* some information has to be gathered from parts of the paper other than the introduction/background/objective section.
**No**	Question or objective is not reported, or is incomprehensible.
**N/A**	Should not be checked for this question

Box 1 Example of a rating criteria for Kmet's quality assessment[34]

RESULTS

This results section provides an overview description of the reviewed studies and their key findings. The findings are considered under four main headings: managerial time spent on quality and safety; managerial quality and safety activities; managerial impact on quality and safety; and contextual factors related to managers' quality and safety role. The section ends with a proposed model to summarise the review findings.

For peer review only - http://bmjopen.bmj.com/site/about/guidelines.xhtml

Description of the studies

From the 19 included studies, the majority were carried out and set in the US (14 studies), and investigated senior management and/or Boards (13 studies). Of these, 3 focused on senior managers alone (e.g. Chief Nursing Officers), 9 concentrated on Board managers and 1 included a mixture of managerial levels. Only 3 investigated middle managers and 3 examined frontline staff (e.g. clinical directorate managers and unit nurse managers). The settings of the study were mostly Trust or hospital-wide; a few articles were set in specific settings or contexts: elderly care,[40] evidence-based medicine,[41] staff productivity,[42] clinical risk management,[43] and hospital acquired infection prevention.[44] Two studies involved specific interventions,[45, 46] and 7 studies concentrated specifically on QI rather than quality and safety oversight or routine.[35, 40, 45-49] There were a mixture of 6 qualitative design (interviews or focus groups); 8 quantitative survey designs; and 5 mix-methods designs. All but one study employed a cross-sectional design[46].[46] The primary outcome measure used in most studies was perceptions of managerial quality and safety practices. All reported participant perceptions and a majority presented self-reports, that is, either a mixture of self and peer reports, or self-reports alone.[41, 43, 45, 46] Several studies asked participants about their own and/or other managers' involvement in regards to their specific quality improvement intervention or quality/safety issue.[40, 41, 44-47] With some variations, the most common research design was to interview or survey senior manager/Board members (particularly Board chairs, presidents and CEOs) perceptions on the Board/senior managers' functions, practices, priorities, agenda, time spent, engagement, challenges/issues, drivers and literacy (e.g. familiarity of key reports) on quality and safety.[35-38, 48-51] Five of these studies included objective process/outcome measures, such as adjusted mortality rates.[35, 37, 38, 49, 50] No other studies included clinical outcome measures.

The quality assessment scores ranged between 50-100%; one study scored (what we consider to be) very low (i.e. <55%), eight studies scored highly (i.e. >75%), two other

For peer review only - http://bmjopen.bmj.com/site/about/guidelines.xhtml

articles scored highly on one out of two of their studies (quantitative/qualitative), and the remaining eight scored a moderate rating in-between. Almost half of the articles did not adequately describe their qualitative studies. Specifically, 8 failed to fully describe their qualitative data collection methods, often not mentioning a standardised topic guide, what questions were asked of participants, or no mention of consent and confidentiality assurances. In 7 studies there was no or vague qualitative data analysis description, including omitting the type of qualitative analysis used. Six of the studies showed no or poor use of verification procedures to establish credibility and 9 reported no or poor reflexivity. Positively, all study designs were evident, the context of studies were clear and the authors showed a connection to a wider body of knowledge.

Similarly to the qualitative studies, 7 quantitative studies did not fully describe, justify or use appropriate analysis methods. However, compared with the qualitative studies, the quantitative studies suffered more from sampling issues. Three studies had particularly small samples (e.g. n=35) and one had an especially low response rate of 15%. Subject characteristics were insufficiently described in 5 studies; in one case the authors did not state the number of hospitals included in data analysis. Several studies had obtained ordinal data but only presented percentages, and only one study appropriately controlled for confounding variables. Across all articles, all but 3 studies reported clear objectives and asserted conclusions clearly supported by the data.

Managerial time spent on quality and safety

The studies on Board level managers highlight an inadequate prioritisation of quality and patient safety on the Board agenda and subsequent time spent at Board meetings. Not all hospitals consistently have quality on their Board agenda, for example CEOs and chairpersons across 30 organisations reported that approximately a third of all Board meetings had quality on their agenda,[35] and necessary quality items were not consistently

For peer review only - http://bmjopen.bmj.com/site/about/guidelines.xhtml

and sometimes never addressed.[36] In all studies examining time spent on quality and safety by the Board, less than half of the total time was spent on quality and safety,[32, 37, 38, 48-51] with a majority of Boards spending 25% or less on quality.[32, 38, 45, 49-51] Findings imply that this may be too low to have a positive influence on quality and safety, as higher quality performance was demonstrated by Boards that spent above 20%/25% on quality.[49, 50] Board members recognised that the usual time spent is insufficient.[48] However, few reported financial goals as more important than quality and safety goals,[32] and health system Boards only spent slightly more time on financial issues than quality.[51] Similar issues are noted by studies on frontline managers; specifically that they placed less time and importance on QI,[42] identified as the least discussed topic by clinical managers.[52]

Managerial quality and safety activities

A broad range of quality-related activities were identified to be undertaken by managers. These are presented by the following three groupings: strategy-centred; data-centred and culture-centred.

Strategy-centred

Board priority-setting and planning strategies aligned with quality and safety goals were identified as Board managerial actions carried out in several studies. High percentages (over 80% in two studies) of Boards had formally established strategic goals for quality with specific targets, and aimed to create a quality plan integral to their broader strategic agenda.[32, 37] Contrary findings however suggest that the Board rarely set the agenda for the discussion on quality,[37] did not provide the ideas for their strategies,[32] and were largely uninvolved in strategic planning for QI.[48] In the latter case, the non-clinical Board managers felt that they held "passive" roles in quality decisions. This is important considering evidence that connects the activity of setting the hospital quality agenda with

For peer review only - http://bmjopen.bmj.com/site/about/guidelines.xhtml

For peer review only

better performance in process of care and mortality.[38] Additionally, Boards that established goals in four areas of quality and publicly disseminated strategic goals and reported quality information were linked to high hospital performance.[35, 38, 50]

Culture-centred

Activities aimed at enhancing patient safety/QI culture emerged from several studies across organisational tiers.[44, 47, 48, 53] Board and senior management's activities included encouraging an organisational culture of QI on norms regarding interdepartmental/multi-disciplinary collaboration and advocating QI efforts to clinicians and fellow senior managers, providing powerful messages of safety commitment and influencing the organisation's patient safety mission.[47, 53] Managers at differing levels focused on cultivating a culture of clinical excellence and articulating the organisational culture to staff.[44] Factors to motivate/engage middle and senior management in QI included senior management commitment, provision of resources and managerial role accountability.[40, 46] Findings revealed connections between senior management and Board priorities and values with hospital performance and on middle management quality-related activities. Ensuring capacity for high quality standards also appears within the remit of management, as physician credentialing was identified as a Board managers' responsibility in more than one study.[38, 48] From this review it is unclear to what degree Board involvement in the credentialing process has a significant impact on quality.[38, 41]

For peer review only

Data-centred

Information on quality and safety are continually supplied to the Board.[51] At all levels of management, activities around quality and safety data or information were recognised in 6 studies.[35, 38, 43, 45, 47, 53] Activities included collecting and collating information,[43] reviewing quality information,[35, 38, 53] using measures such as incident reports and infection rates to forge changes,[53] using patient satisfaction surveys,[35] taking corrective

For peer review only - http://bmjopen.bmj.com/site/about/guidelines.xhtml

action based on adverse incidents or trends emphasised at Board meetings,[38] and providing feedback.[43, 47] The studies do not specify the changes made based on the data-related activities by senior managers; one study identified that frontline managers predominantly used data from an incident reporting tool to change policy/practice and training/education and communication between care providers.[45] However, overseeing data generally was found to be beneficial, as hospitals that carried out performance monitoring activities had significantly higher scores in process of care and lower mortality rates than hospitals that did not.[38]

Managerial impact on quality and safety outcomes

We have considered the associations found between specific managerial involvement and its affect on quality and safety. Here, we summarise the impact and importance of their general role. Of the articles that looked at either outcomes of management involvement in quality or at its perceived importance, 6 articles suggested that their role was beneficial to quality and safety performance.[32, 35, 38, 40, 49, 53] Senior management support and engagement was identified as one of the primary factors associated with good hospital-wide quality outcomes and QI programme success.[35, 38, 40, 49] Conversely, 6 articles suggest that managers' involvement (from the Board, middle and frontline) has little, no or a negative influence on quality and safety.[35, 38, 41, 42, 44, 49] Practices that showed no significant association with quality measures included Board's participation in physician credentialing.[35, 38] Another noted that if other champion leaders are present, management leadership was not deemed necessary.[44] Two articles identified a negative or inhibitory effect on evidence-based practices and staff productivity from frontline and middle managers.[41, 42]

For peer review only - http://bmjopen.bmj.com/site/about/guidelines.xhtml

Contextual factors related to managers' quality and safety role

Most of the articles focussed on issues that influenced the managers' role or their impact, as opposed to discussing the role of the managers. These provide an insight in to the types of conditions in which a manager can best undertake their role to affect quality and safety. Unfortunately it appears that many of these conditions are not in place.

Two studies found that a Board quality committee is a positive variable in quality performance, but that fewer than 60% had them.[38, 50] Similarly, compensation and performance evaluation linked to executive quality performance was identified in 4 articles [35, 37, 38, 49] and associated with better quality performance indicators,[38, 49] but quality measures were insufficiently included in CEOs' performance evaluation.[35, 37] The use of the right measures to drive QI was raised in relation to Board managerial engagement in quality [35] and to impact on patient care improvement,[51] yet, almost half of this sample did not formally adopt system-wide measures and standards for quality. To aid them in these tasks, evidence indicates the common use of QI measure tools, such as a dashboard or scorecard, [37, 49, 50] with promising associations between dashboard use and quality outcomes.[38, 50]

Other factors linked to quality outcomes include management-staff relationship/high interactions between the Board and medical staff when setting quality strategy,[49] and managerial expertise. Although a connection between knowledge with quality outcomes was not found,[38] high performing hospitals have shown higher self-perceived ability to influence care, expertise at the Board and participation in training programs that have a quality component.[50] Disappointingly, there is a low level of CEO knowledge on quality and safety reports,[35] possibly little Boardroom awareness on salient nursing quality issues,[36] and little practice identified to improve quality literacy for the Board.[32, 37] There is however promise for new managers through relevant training at induction and by recruitment of those with relevant expertise.[32]

For peer review only - http://bmjopen.bmj.com/site/about/guidelines.xhtml

The Quality Management IPO Model

The input process output (IPO) model is a conceptual framework that helps to structure the review findings in a useful way, please see Figure 2.[54, 55] This literature may be conceptualised by considering what factors contribute (input) to managerial activities (process) that impact on quality and safety (output). The three factors are interrelated and interchangeable, presented by the cyclical interconnecting diagram. This diagram enables a clearer mental picture of what a manager should consider for their role in quality and safety. Specifically, the input factors suggest certain organisational factors that should be put in place alongside individual factors to prepare for such a role (e.g. standardised quality measures, motivation, education and expertise, and a good relationship with clinicians). The processes present the strategy, culture and data-centred areas where managers (according to the literature) are and/or should be involved (e.g. driving improvement culture, goal-setting and providing feedback on corrective actions for adverse events). The outputs identify managerial influences that are positive, negative or have little or no established association with quality performance (e.g. positive outcomes of care, achieving objectives and engaging others in quality of care). This helps to identify areas where it is possible to make an impact through the processes mentioned. With further empirical studies on this topic, this model could be strengthened to become a more robust set of evidence-based criteria and outcomes.

For peer review only

First author; Year [Country]	Methods	Sample size [number of organisations]	Population sample [Level of management reported on (position of managers)]	Outcome Measure	Management Roles (Managerial quality and safety activities, time spent and engagement & key perceived importance and context factors)	Quality assessment score for qualitative studies	Quality assessment score for quantitative studies	Findings pertaining to research questions *[Time spent; Activities, Engagement; Impact (including perceived effectiveness); Contextual factors]*
Baker et al, 2010 [Canada][32]	Mixed methods (interviews, case studies, surveys)	n=15 interviews; n=4 Board case studies; n=79 surveys [79 organisations]	Managers [Board management]	Perceptions of managers on management Board practices in quality and safety	• Less than half (43%) of Boards reported that they addressed quality and patient safety issues in all meetings<br>• One-third of Boards spend 25% of their time or more on quality and patient safety issues<br>• More than 80% of Boards have formally established strategic goals for quality with specific targets, but a majority of Board chairs indicate that their Boards did not provide the ideas for strategic direction or initiatives<br>• Board chairs reported a low participation in education on quality and safety: 43% reported that all the Board members participated, 19% stated that more than half participated and 23% said it was less than a quarter of the Board<br>• Most Board chairs (87%) reported Board member induction training on responsibilities for quality and safety, although almost a third (30%) reported few or no opportunities for education on this, 42% reported some opportunities and 28% reported many)<br>• Approximately half (57%) of the Board chairs acknowledged recruitment of individuals that have knowledge, skills and experience in quality and patient safety onto the Board. A Board skills matrix included quality and safety as one of the competency areas<br>• Over half (55%) of board chairs rated their board's effectiveness in quality and safety oversight as very/extremely effective and 40% as somewhat effective.	16/20 (80%)	12/22 (55%)	Time Activities Impact Context
Balding 2005 [Australia] [46]	Mixed methods (action research, s urveys & focus groups)	n=35 [1 hospital]	Managers [Middle management (Nursing managers and allied health managers)]	Self-reported perceptions of managers on their engagement in a QI programme	Five elements deemed essential to middle manager engagement:<br>• (1) Senior management commitment and leadership (e.g. senior management provides strategic direction for QI plan)<br>• (2) Provision of resources and opportunities for QI education and information dissemination (e.g. basic QI skills provided to all staff)<br>• (3) Senior and middle manager role accountability (e.g.: senior managers and middle managers agree QI roles and expectations)<br>• (4) Middle manager involvement in QI planning (e.g. senior and middle managers plan together)<br>• (5) Middle managers own and operate QI program (e.g. ongoing review and evaluation of the progress of the QI program by the middle and senior managers)	14/20 (70%)	15/22 (68%)	Activities Engagement Impact

For peer review only - http://bmjopen.bmj.com/site/about/guidelines.xhtml

Bradely et al, 2003 [US][47]	Qualitative (interviews)	n=45 [8 hospitals]	Clinical staff and senior management [Senior management (unspecified)]	Perceptions of roles and activities that comprise senior management's involvement in quality improvement efforts	Five common roles and activities that captured the variation in management involvement in quality improvement efforts:<br>• (1) Personal engagement of senior managers<br>• (2) Management's relationship with clinical staff<br>• (3) Promotion of an organisational culture of quality improvement<br>• (4) Support of quality improvement with organisational structures<br>• (5) Procurement of organisational resources for quality improvement efforts	19/20 (95%)	NA	Activities Engagement Impact
Bradely et al, 2006 [US][40]	Mixed-methods (surveys & interviews)	n=63 survey respondents [63 hospitals]; n=102 interviewees [13 hospitals]	Managers [Senior management (chief operating officer, vice president, medical director, chief nursing officer, director of volunteers, program director)]	Perceptions of management-related factors around the HELP programme	• Providing resources for needed staffing or staff training<br>• Promoting the program among the governing Board, physicians who were initially less involved, and other administrators<br>• Senior management support reported as the primary enabling factor in the implementation of such programmes (96.6%), along with a lack of support as the primary reason for not implementing the programme (65.0%)<br>• The interviews supported that having an administrative champion was considered essential to their program's success	19/20 (95%)	17/22 (77%)	Activities Engagement Impact Context
Braithwaite et al, 2004 [Australia] [52]	Mixed methods (ethnographic work, observations & focus groups)	n=64 managers in focus groups [1 hospital]; Ethnographic case studies and n=4 observed [2 hospitals]	Managers [Frontline management (Medical managers, nurse managers & allied health managers)]	Observations and self-reported perceptions of clinician-managers' activities	• Quality was the least discussed topic (e.g. Continuous quality improvement)<br>• The most discussed topic was people (e.g. staffing, delegating) and organisational issues, e.g. beds and, equipment	16/20 (80%)	NA	Time

For peer review only - http://bmjopen.bmj.com/site/about/guidelines.xhtml

Caine & Kenwrick, 1997[UK][41]	Qualitative (interviews)	n=10 [2 hospitals]	Managers [Middle management (Clinical directorate managers)]	Self-reported perceptions of managers on the managers' role in facilitating evidence-based practice in their nursing teams	• Managers saw their role in research implementation as a facilitator, ensuring quality and financial objectives and standards were met<br>• Managers perceived their facilitatory behaviours produced a low level of clinical change.<br>• Managers are not actively advocating research-based practice and failing to integrate it into everyday practice. Their behaviour inhibited the development of evidence-based nursing practice<br>• Devolved responsibility of use of research to individual professionals	14/20 (70%)	NA	Activities Impact
Fox, Fox & Wells 1999 [US][42]	Quantitative (surveys & self kept activity logs)	n=16 [1 hospital]	Managers [Frontline management (Nurse administrative managers (NAMs))]	Self-reported perceptions of managers on their activities impacting unit personnel productivity & monitored time/effort allocated to each function & managers' hours worked, patient admissions and length of stay.	• The small amount of total management allocated to QI (2.6%) was the least time spent of all management functions<br>• A negative relationship between time spent in QI activities and unit personnel productivity. An increase (from 2.5% to 5%) in QI time/effort by NAMs would reduce staff productivity significantly by approximately 8%.<br>• The greater the experiences of NAMs as managers, the more time spent on QI. These seasoned NAMs spent more time on monitoring, reporting QI results, and quality improvement teams (statistics nor provided)	NA	13/22 (59%)	Time Activities Impact (Objective outcome measure)
Harris, 2000 [UK][43]	Quantitative (surveys)	n=42 [42 hospitals]	Managers [Middle management (Nurse managers)]	Self-reported perceptions of managers on managers' quality and safety practices	• The majority of managers (91%) who received collated incident information used it to feed back to their own staff. 60% always fed back to staff, 28% sometimes did, 2% never did.<br>• Of the Trusts that had written guidance on types of clinical incident to report, 80% of managers had general guidance and fewer (20%) had written speciality specific guidance.<br>• 76% of managers reported information collation of clinical incidents. Of these, 59% were involved in data collection themselves	NA	13/22 (59%)	Activities

For peer review only - http://bmjopen.bmj.com/site/about/guidelines.xhtml

Jha and Epstein, 2010 [US][50]	Quantitative (surveys)	n=722 [767 hospitals]	Managers [Board]	Perceptions of managers on the role of managers in quality and safety & quality outcome measurement (from Hospital Quality Alliance) i.e. 19 practices for care in 3 clinical conditions	• Two-thirds (63%) of Boards had quality as an agenda item at every meeting<br>• Fewer than half (42%) of the hospitals spent at least 20% of the Board's time on clinical quality<br>• 72% of Boards regularly reviewed a quality dashboard<br>• Most respondents reported that their Boards had established, endorsed, or approved goals in four areas of quality: hospital-acquired infections (82%), medication errors (83%), the HQA/Joint commission core measures (72%), and patient satisfaction (91%)<br>High-performing hospitals were more likely than low-performing hospitals to have:<br>• Board reviews of a quality dashboard regularly (<0.001) & of clinical measures (all <0.05)<br>• Quality performance on the agenda at every Board meeting (0.003)<br>• At least 20% of Board time on clinical quality (0.001)<br>• Has a quality subcommittee (0.001)	NA	22/22 (100%)	Time Activities Impact (Objective outcome measure) Context
Jiang et al, 2008 [US][37]	Quantitative (surveys)	n=562 [387 hospitals]	Managers [Board & Senior management (presidents/ CEOs)]	Perceptions of managers on managers' practices in quality and safety; and outcomes of care (composite scores of risk-adjusted mortality indicators)	• 75% of CEOs reported that most to all of the Board meetings have a specific agenda item devoted to quality. Only 41% indicated that the Boards spend more than 20 percent of its meeting time on the specific item of quality.<br>The following activities were most reported to be performed:<br>• Board establishing strategic goals for QI (81.3%)<br>• Use quality dashboards to track performance (86%)<br>• Follow up corrective actions related to adverse events (83%)<br>The following activities were least reported to be performed:<br>• Board involvement in setting the agenda for the discussion on quality (42.4%)<br>• Inclusion of the quality measures in the CEO's performance evaluation (54.6%)<br>• Improvement of quality literacy of Board members (48.9%)<br>• Board written policy on quality and formally communicated it (30.8%)	NA	20/26 (77%)	Time Activities Impact (Objective outcome measure) Context
Jiang et al, 2009 [US][38]	Quantitative (surveys)	n=490 [490 hospitals]	Managers [Board & Senior management (CEOS & Hospital presidents reports)]	Perceptions of managers on manager's practices in quality and safety; and process of care measures (20 measures in 4 clinical areas); and outcome measures (composite scores of risk-adjusted	Board practices found to be associated with better performance (all P<0.05) in process of care (POC) and adjusted mortality (M) included:<br>• Having a Board quality committee (83.8%POC, 6.2M versus 80.2%POC, 7.9M without a committee)<br>• Establishing strategic goals for quality improvement (82.8%POC, 6.6M versus 80.3%POC, 7.9M)<br>• Being involved in setting the quality agenda for the hospital (83.2%POC, 6.4M versus 80.9%POC, 7.7M)<br>• Including a specific item on quality in Board meetings (83.2%POC, 6.5M versus 78.5%POC, 8.6M)<br>• Using a dashboard with national benchmarks and internal data that includes indicators for clinical quality, patient safety, and patient satisfaction (all above 80%POC & below 6.5M versus all below 80%POC and above 7M)<br>• Linking senior executives' performance evaluation to quality and patient safety indicators (83.1%POC, 6.6M versus 80.4%POC, 7.6M)<br>Practices that did NOT show significant association with the quality measures for process and mortality include:<br>• Reporting to the Board of any corrective action related to adverse events	NA	22/24 (92%)	Activities Impact (Objective outcome measure) Context

For peer review only

For peer review only - http://bmjopen.bmj.com/site/about/guidelines.xhtml

				mortality indicators)	(82.5%POC, 7.0M versus 81.8%POC, 6.6M)<br>• Board's participation in physician credentialing (82.8%POC, 6.9M versus 81.5%POC, 6.9M)<br>• Orientation for new Board members on quality(82.9%POC, 6.8M versus 81.7%POC, 7.0M)<br>• Education of Board members on quality issues (82.8%POC, 7.0M versus 81.9%POC, 6.9M)			
Joshi & Hines, 2006 [US][35]	Mixed-methods (surveys & interviews)	n=37 survey respondents; n=47 interviewees [30 hospitals]	Managers [Board & Senior management (CEOs, Board chairs)]	Perceptions of managers on managers' practices in quality and safety and appropriate care measure (ACM) and risk adjusted mortality.	• Board engagement in quality was reported as satisfactory (7.58 by CEOs and 8.10 by Chairs on a 1-10 scale where 10 indicates greatest satisfaction)<br>• Board engagement was positively associated with perceptions of the rate of progress in improvement (r=.44, p = .05), and marginally associated with ACM scores (r=.41, p=.07)<br>• Approximately one third of Board meetings are devoted to discussing quality issues (reported at 35% by CEOs and 27% by Chairs)<br>• Integrating Quality Planning and Strategic Planning was also rated as satisfactory (7.67 by CEOs and 8.85 by Chairs)<br>• Approximately two-thirds of respondents reported using patient satisfaction surveys (70% and 65% reported by CEOs and Chairs respectively)<br>• Low level of CEO expertise in QI, as reported by themselves (2.70) and by Board Chairs (3.35%) on a scale of 1-10 where 1 is low familiarity and 10 is high familiarity.	12/20 (60%)	16/20 (80%)	Time Activities Engagement Impact (Objective outcome measure) Context
Levey et al, 2007 [US][48]	Qualitative (Interviews)	n=96 [18 hospitals]	Managers [Board & Senior management (Hospital Board members, CEOs, Chief Medical Officers, Chief Quality Officers, medical staff leaders)]	Perceptions of managers on managers' role in quality and safety	• Few CEOs were willing to take the lead for transformation to a "culture of quality"<br>• Board members were largely uninvolved in strategic planning for QI<br>• In terms of the Board's quality functions, respondents largely agreed that physician credentialing was their critical responsibility<br>• Non-physicians reported that they felt relegated to "passive" roles in decisions on quality and seemed reluctant to assume leadership roles in the quality domain.<br>• Board meeting agendas maintained a focus on financial issues, although patient safety/care and QI were gaining prominence<br>• About half of the respondents said that quality was not sufficiently highlighted during meetings. Estimates of time devoted to quality and safety issues at Board meetings were between 10% to 35%	13/20 (65%)	NA	Time Activities Engagement Context
Mastal, Joshi & Shulke 2007 [US][36]	Qualitative (interviews and a focus group)	n=73 interviewees; 1 focus group [63 hospitals]	Managers [Board & Senior management (Board chairs, CEOs, Chief Nurse Officers)]	Perceptions of managers on managers' role in quality and safety	• Two chief nursing officers (CNOs) reported that nursing quality was never addressed at Board meetings.<br>• Few of the CNOs, CEOs, and Board chairs responded that issues are discussed more frequently, such as at every meeting.<br>• Quality and patient safety measures for nurses are not consistently addressed during all hospital Board meetings.<br>• Staffing concerns are the most frequent measure of nursing quality reported at the Board level	12/20 (60%)	NA	Time Context

For peer review only - http://bmjopen.bmj.com/site/about/guidelines.xhtml

Poniatowski, Stanley & Youngberg, 2005 [US][45]	Quantitative (surveys)	n=515 [16 academic medical centers]	Managers [Frontline management –unclear whether frontline or middle managers (Unit Nurse managers)]	Self-reported perceptions of managers on their practices with Patient Safety Net (PSN)	• Managers reviewed on average 65% of the PSN events reported<br>As a result of what was learned from PSN data, 162 managers detailed their changes made to:<br>• Policies and practices (59%)<br>• Training, education and communication between care providers (27%)<br>• Purchase of new equipment and supplies (8%)<br>• Staffing (6%)	N/A	10/20 (50%)	Activities
Prybil et al, 2010(US) [51]	Quantitative (surveys)	n=123 [712 hospitals]	Managers [Board & Senior management (CEOs & Boards)]	Perceptions of managers on their role in quality and safety	• Health system Boards spent 23% of their Board meeting time on quality and safety issues. They only spent slightly more on financial issues (25.2%) and strategic planning (27.2%)<br>• Almost all (96%) CEOs said that the Boards regularly received formal written reports on quality targets<br>• 88% of CEOs said that the Boards had assigned quality and safety oversight to a standing Board committee<br>• All but one (98.9%) of the CEOs stated that they have specific performance expectations and criteria related to quality and safety<br>• CEOs reported 59% of the Boards formally adopted system-wide measures and standards for quality	N/A	14/22 (64%)	Time<br>Activities<br>Context
Saint et al, 2010 [US][44]	Qualitative (interviews)	n=86 (interviewees) [14 hospitals]	Senior hospital staff and managers [Mixed levels (Nurse managers, chief physicians, Chairs of medicine, Chief of Staffs, hospital directors, CEOs & clinical non-managerial staff)]	Perceptions of managers on managers' practices in healthcare-associated infection (HAI)	• Although committed leadership by CEOs can be helpful, it was not always necessary, provided that other hospital leaders were committed to infection prevention<br>Behaviours of leaders who successfully implemented/facilitated practices to prevent HAI:<br>• Cultivated a culture of clinical excellence and kept their eye on improving patient care<br>• Developed a vision<br>• Articulated the organisational culture well and conveyed that to staff at all levels.<br>• Focused on overcoming barriers and dealing directly with resistant staff or process issues that impeded prevention of HAI<br>• Cultivated leadership skills and inspired the people they supervised (motivating and energising them to work towards the goal of preventing HAI)<br>• Thought strategically while acting locally; planned ahead and left few things to chance<br>• They did the politicking before issues arose for committee votes<br>• They leveraged personal prestige to move initiatives forward<br>• They worked well across disciplines	16/20 (80%)	NA	Activities<br>Engagement<br>Impact

For peer review only - http://bmjopen.bmj.com/site/about/guidelines.xhtml

Vaughn et al, 2006 (US)[49]	Quantitative (surveys)	n=413 [413 hospitals]	Managers [Board & Senior management (chief executives & senior quality executives; Board, executives, clinical leadership)]	Perceptions of managers on managers' role in QI & observed hospital Quality index outcomes (risk-adjusted measures of morbidity, mortality, and medical complications)	• 72% of hospital Boards spent one-quarter of their time or less on quality-of-care issues. About 5% of Boards spent more than half of their time on these issues<br>• A majority of respondents reported great influence from government and regulatory agencies (87%), consumers (72%) and accrediting bodies (74%) on quality priorities. Although 44% of respondents also noted that multiple government and regulatory requirements were unhelpful<br>Better quality index scores (QIS) are associated with hospitals where the Board:<br>• Spends more than 25% of their time on quality issues (QIS 83 – QIS mean 100 across hospitals)<br>• Receives a formal quality performance measurement report (QIS 302)<br>• Bases the senior executives' compensation in part on QI performance (QIS 239)<br>• Engages in a great amount of interaction with the medical staff on quality strategy	NA	21/22 (95%)	Time<br>Activities<br>Engagement<br>Impact (Objective outcome measure)<br>Context
Weingart & Page, 2004 [US][53]	Qualitative (case study documentation analysis and meeting discussions and focus group)	n=30 [10 hospitals and other stakeholder organisations]	Managers [Senior management (Executives)]	Perceptions of managers on manager's practices in quality and safety	Executives developed and tested a set of governance best practices in patient safety, such as:<br>• Creation of a Board committee with explicit responsibility for patient safety<br>• Development of Board level safety reports, introduction of educational activities for Board members<br>• Participation of Board members in executive walk rounds.<br>• Executives reviewed measures to assess safety (e.g. incident reports, infection rates, pharmacist interventions, readmissions, etc)<br>• Executives endorsed a statement of public commitment to patient safety.<br>• Executives believed their behaviours affected their organisations' patient safety mission	14/20 (70%)	NA	Activities<br>Impact

Table 1 Table of characteristics and summary findings of included studies

----

Enter Figure 2

For peer review only - http://bmjopen.bmj.com/site/about/guidelines.xhtml

DISCUSSION

Our review examined the role of managers in maintaining and promoting safe, quality care. The existing studies detail the time spent, activities and engagement of hospital managers and Boards, and suggest that these can positively influence quality and safety performance. They further reveal that such involvement is often absent, as are certain conditions that may help them in their work.

Evidence from the review promotes hospitals to have a Board quality committee, with a specific item on quality at the Board meeting, a quality performance measurement report and a dashboard with national quality and safety benchmarks along with standardised quality and safety measures. Outside of the Boardroom, the implications are for senior managers to build a good infrastructure for staff-manager interactions on quality strategies and attach compensation and performance evaluation to quality and safety achievements. For QI programmes, managers should keep in mind its consistency with the hospital's mission and provide commitment, resources, education, and role accountability. Literature elsewhere supports much of these findings, such as the use of quality measurement tools [21, 56] better quality-associated compensation, a separate quality committee,[16, 57] and has also emphasised poor manager-clinician relationships as damaging to patients and QI.[58, 59]

Some of the variables that were shown to be associated with good quality performance, such as having a Board committee, compensation/performance and adoption of system-wide measures, were lacking within the study hospitals. There are also indications of the need to develop Board and senior managerial knowledge and training on quality and safety. Furthermore, this review indicates that many managers do not spend sufficient time on quality and safety. The included studies suggest time spent by the Board should exceed 20-25%, yet the findings expose that certain Boards devote less time than this. Inadequacies of time allocated to quality at the Board meeting hold concerning implications for quality. If little

For peer review only

time is taken to consider quality of care matters at the highest level, an inference is that less attention will be paid to prevention and improvement of quality within the hospital. While the position that the item appears on the agenda is deemed of high importance, it is unimportant if the duration on this item is overly brief. In this vein, the inadequate time on quality spent by some may reflect their prioritisation on quality in relation to other matters discussed at the meetings or the value perceived to be gained from discussing it further. It might instead however be indicative of the difficulties in measuring time spent on quality by management. Some of these studies provide us not necessarily with Board managers' time on quality and safety but their time spent on this at Board meetings. The two may not equate and time spent on quality may not necessarily be well-spent.[36] The emerging inference that managers greatly prioritise other work over quality and safety is not explicit, with further research required to identify what time is actually devoted and required from managers inside and outside of the Boardroom. Perhaps encouragingly, the more recent studies present more time spent on quality and safety than the earlier studies. Yet even the most recent empirical studies not included in our review conclude that much improvement is required.[60]

This review presents a wide-range of managerial activities, such as public reporting of quality strategies and driving an improvement culture. It further highlights the activities that appear to affect quality performance. Priorities for Boards/managers are to engage in quality, establish goals and strategy to improve care, and get involved in setting the quality agenda, support and promote a safety and QI culture, cultivate leaders, manage resisters, plan ahead and procure organisational resources for quality. Again, much of the findings support the assertions made in the non-empirical literature. Above all, involvement through action, engagement and commitment have been suggested to positively affect quality and safety.[61] While researchers have stressed the limited empirical evidence showing conclusive connection between management commitment and quality,[21] some supporting evidence however can be unearthed in research that concentrates on organisational factors

For peer review only

related to changes made to improve quality and safety in healthcare.[62-64] In addition to this evidence, a few studies have specifically investigated the impact that hospital managers have on quality and safety (rather than examination of their role). These studies have shown senior managerial leadership to be associated with a higher degree of QI implementation,[65] promotion of clinical involvement,[66, 67] safety climate attitudes,[68] and increased Board leadership for quality.[57] A clear case for the positive influence of management involvement with quality is emerging both from the findings of our review and related literature.

There is a dearth of empirical research on the role of hospital managers in quality of care and patient safety and quality improvement. This evidence is further weakened by the largely descriptive nature of many of the studies. They most lack theoretical underpinnings and appropriate objective measures. Very few studies reported objective clinical quality outcome measures that better show the influence of managerial actions. Moreover, the content of many of the articles was dominated by the contextual issues surrounding managers' roles, rather than actual manager practices. Some of the outlined managerial actions would further benefit from more detail, for example, the literature fails to present changes made based on data-related activities at the Board or senior management level. Only one study clearly demonstrated that senior management and Board priorities can impact upon middle management quality-related activities and engagement. Considering the likely influence that seniors have on their managers, examination of the interactions between the different roles held (e.g. Boards setting policies on quality and middle managers implementing them) would improve our understanding of how these differences reflect in their time spent and actions undertaken. Supplementary work could also resolve contradictions that were found within the review, clarifying for example the positive impact of managerial expertise versus knowledge on quality and who sets the Board agenda for the discussion on quality. Research on this area is particularly required to examine middle and frontline managers, to take into consideration non-managers' perceptions, and to assess senior managers' time

For peer review only

and tasks outside of the Boardroom. Future studies would benefit from better experimental controls, ideally with more than one time point, verifications and reflections on qualitative work, robust statistical analysis, appropriate study controls, consideration of confounding variables, and transparent reporting of population samples, methodologies, and analyses used. Box 2 presents the key messages from this review.

Review limitations

There are several limitations of the present review pertaining to the search strategy and review process, the limited sample of studies, publication bias, and limitations of the studies themselves. Specifically, the small number of included studies and their varied study aims, design and population samples make generalisations difficult. Grouped demographics, such as middle management, are justified by the overlap between positions. With more literature on this topic, distinctions could be made between job positions. Furthermore, more research on lower levels of management would have provided a better balanced review of hospital managers work and contributions to quality. Restricting the language of studies to English in the search strategy is likely to have biased the findings and misrepresent which countries conduct studies on this topic. There is an over-reliance on perceptions across the studies, which ultimately reduces the validity of the conclusions drawn from their findings. As most of the study findings relied on self-reports, social desirability may have resulted in exaggerated processes and inflated outputs. Although, encouragingly, one of the included studies found that managers that perceived their Boards to be effective in quality oversight were from hospitals that had higher processes-of-care scores and lower risk adjusted mortality. The quality assessment scores should be viewed with caution; such scores are subjective and may not take into consideration factors beyond the quality assessment scale used. Due to the enormity of this review, the publication of this article is some time after the search run date. As there is little evidence published on this topic, we consider this not to greatly impact on the current relevance of the review, particularly as the literature reviewed spans almost

For peer review only

three decades. However, we acknowledge the need for an update of the data as a limitation of this review.

Conclusion

The modest literature that exists suggests that managers' time spent, engagement and work can influence quality and safety clinical outcomes, processes and performance. Managerial activities that affect quality performance are especially highlighted by this review, such as establishing goals and strategy to improve care, setting the quality agenda, engaging in quality, promoting a quality improvement culture, managing resisters, and procurement of organisational resources for quality. Positive actions to consider include the establishment of a Board quality committee with a specific item on quality at the Board meeting, a quality performance measurement report and a dashboard with national quality and safety benchmarks, performance evaluation attached to quality and safety, and an infrastructure for staff-manager interactions on quality strategies. However, many of these arrangements were not in place within the study samples. There are also indications of a need for managers to devote more time to quality and safety. More than one study suggest time spent by the Board should exceed 20-25%, yet the findings expose that certain Boards devote less time than this. Much of the content of the articles focused on such contextual factors rather than on the managerial role itself; more empirical research is required to elucidate managers' actual activities. Research is additionally required to examine middle and frontline managers, non-manager perceptions, and to assess senior managers' time and tasks outside of the Boardroom. We present the IPO model to summarise the evidence-based promotion of conditions and activities in order to guide managers on the approaches taken to influence quality performance. More robust empirical research with objective outcome measures could strengthen this guidance.

For peer review only

- There is a dearth of empirical evidence on hospital managerial work and its influence on quality of care.
- There is some evidence that Boards'/managers' time spent, engagement and work can influence quality and safety clinical outcomes, processes and performance.
- Some variables associated with good quality performance were lacking in study hospitals.
- Many Board managers do not spend sufficient time on quality and safety.
- There is a greater focus on the contextual issues surrounding managers' roles than on examining managerial activities.
- Research is required to examine middle and frontline managers, to take into consideration non-managers' perceptions, and to assess senior managers' time and tasks outside of the Boardroom. More robust methodologies with objective outcome measures would strengthen the evidence.
- We present a model to summarise the evidence-based promotion of conditions and activities for managers to best affect quality performance.

Box 2 Key messages from the systematic literature review

CONTRIBUTORS

All co-authors contributed to the study design and review of drafts of the article. The first author screened all of the articles for inclusion in this review and appraised the study quality. Dr Anna Renz and Miss Dina Grishin screened a sample of these at title/abstract and full text, and Miss Ana Wheelock scored the quality of a sample of the included articles.

ACKNOWLEDGEMENTS

We would like to thank Miss Dina Grishin for helping to review the abstracts and Miss Ana Wheelock for helping to assess the quality of the articles.

COMPETING INTERESTS

There are no competing interests.

FUNDING

This work was supported by funding from the Health Foundation and the National Institute for Health Research.

Figure Legends

Figure 1 Review stages based on PRISMA Flow Diagram[33]
Figure 2 The Quality Management IPO model

For peer review only

REFERENCES

1. Kizer KW. Patient Safety: A Call to Action: Safety Is a Critical Component of Quality. Medscape General Medicine 2001;**3:10**.
2. Kizer KW. Establishing health care performance standards in an era of consumerism. Journal of the American Medical Association 2001;**286**:1213-7.
3. Berwick D. Will is the way to win the patient safety war. Health Serv J 2007;**117**:18-9.
4. Institute of Medicine. Crossing the quality chasm: a new health system for the 21st century. Washington: National Academy Press; 2001.
5. Gosfield AG, Reinertsen JL. The 100,000 lives campaign: crystallizing standards of care for hospitals. Health aff 2005;**24**:1560–70.
6. Denham CR. Is your hospital as safe as your bank?...Time to ask your board. J Patient Saf 2009;**5**:122-6.
7. Francis R. Report of the Mid Staffordshire NHS Foundation Trust Public Inquiry London: The Stationery Office 2013 [accessed on 18.03.13]. Available from: http://www.midstaffspublicinquiry.com/report.
8. National Advisory Group on the Safety of Patients in England. A promise to learn – a commitment to act: Improving the Safety of Patients in England 2013 [accessed on 15.08.13]. Available from: https://www.gov.uk/government/uploads/system/uploads/attachment_data/file/226703/Berwick_Report.pdf.
9. O'Dea A, Flin R. Site managers and safety leadership in the offshore oil and gas industry. Safety Sci 2001;**37**:39-57.
10. Zohar D, Luria G. The use of supervisory practices as leverage to improve safety behavior: a cross-level intervention model. Journal of Safety Res 2003;**34**:567-77.
11. Flin R. "Danger-Men at Work": Management Influence on Safety. Hum Factor and Ergon Man 2003;**13**:261-8.
12. Guidotti TL. How can managers visibly promote safety and health protection? J Occ Env Med 2010;**52**:1141-2.
13. Conway J. Getting boards on board: engaging governing boards in quality and safety. Jt Comm J Qual Patient Saf 2008;**34**:214-20.
14. Gautam KS. A call for board leadership on quality in hospitals. Qual Manag Health Care 2005;**14**:18-30.
15. Caldwell C, Butler G, Grah J. Breakthrough quality: what the board must do. Trustee 2008;**61**:32-3, 1.
16. Goeschel CA, Wachter RM, Pronovost PJ. Responsibility for quality improvement and patient safety: hospital board and medical staff leadership challenges. Chest 2010;**138**:171-8.
17. McGinn P, Dave R. What is the board's role in quality? Trustee 2007;**60**:40.
18. Øvretveit J. Leading improvement effectively: Review of research 2009 [accessed on 21.12.12]. Available from: http://www.health.org.uk/public/cms/75/76/313/560/Leading%20imrpovement%20effectively.pdf?realName=YUaqJk.pdf.
19. Flin R, Yule S. Leadership for safety: industrial experience. Qual and Saf Health Care 2004;**13**:45-51.

For peer review only

20. West E. Management matters: the link between hospital organisation and quality of patient care. Qual and Saf Health Care 2001;**10**:40-8.

21. Glickman SW, Baggett KA, Krubert CG, Peterson ED, Schulman KA. Promoting quality: the health-care organization from a management perspective. Int J Qual Health Care 2007;**19**:341-8.

22. Gaucher E, Kratochwill EW. The leader's role in implementing total quality management. Qual Manag Health Care 1993;**1**:10-8.

23. Thorman KE, Capitulo KL, Dubow J, Hanold K, Noonan M, Wehmeyer J. Perinatal patient safety from the perspective of nurse executives: a round table discussion. JOGNN 2006;**35** 409-16.

24. Aspden P, Corrigan JM, Wolcott J, Erickson SM. Patient safety: achieving a new standard of care. Institute of Medicine, editor. Washington: National Academies Press; 2004.

25. AHRQ. AHRQ Patient Safety Network Glossary 2011 [accessed on 19.05.12]. Available from: http://www.psnet.ahrq.gov/glossary.aspx?indexLetter=P.

26. Merali F. NHS managers’ commitment to a socially responsible role: the NHS managers' views of their core values and their public image. Social Responsibility Journal 2005;**1**:38-46.

27. Mintzberg H. The Nature of Managerial Work. New York: Harper & Row; 1973.

28. Tanon AA, Champagne F, Contandriopoulos AP, Pomey MP, Vadeboncoeur A, Nguyen H. Patient safety and systematic reviews: finding papers indexed in MEDLINE, EMBASE and CINAHL. Quality saf health care 2010;**19**:452-61.

29. Cohen J. A coefficient for agreement for nominal scales. Educ Psych Meas 1960;**20**:37–46.

30. Cicchetti DV, Feinstein AR. High agreement but low kappa: II. Resolving the paradoxes. J Clin Epidemiol 1990;**43**:551-8.

31. Viera AJ, Garrett JM. Understanding interobserver agreement: the kappa statistic. Family med 2005;**37**:360-3.

32. Baker GR, Denis JL, Pomey MP, MacIntosh-Murray A. Effective governance for quality and patient safety in Canadian Healthcare organizations: a report to the Canadian Health Services Research Foundation and the Canadian Patient Safety Institute. Ottawa: Canadian Health Services Research Foundation and Canadian Patient Safety Institute, 2010.

33. Moher D, Liberati A, Tetzlaff J, Altman DG. Preferred reporting items for systematic reviews and meta-analyses: the PRISMA statement. Ann Intern Med 2009;**151**:264-9.

34. Kmet LM, Lee RC, Cook LS. Standard Quality Assessment Criteria for Evaluating Primary Research Papers from a Variety of Fields. Edmonton: Alberta Heritage Foundation for Medical Research, 2004.

35. Joshi MS, Hines SC. Getting the board on board: Engaging hospital boards in quality and patient safety. Jt Comm J Qual Patient Saf 2006;**32**:179-87.

36. Mastal MF, Joshi M, Schulke K. Nursing leadership: championing quality and patient safety in the boardroom. Nurs Econ 2007;**25**:323-30.

37. Jiang HJ, Lockee C, Bass K, Fraser I. Board engagement in quality: findings of a survey of hospital and system leaders. J Healthc Manag 2008;**53**:121-34.

38. Jiang HJ, Lockee C, Bass K, Fraser I. Board oversight of quality: Any differences in process of care and mortality? J Healthc Manag 2009;**54**:15-29.

39. Walsh D, Downe S. Meta-synthesis method for qualitative research: a literature review. J Adv Nurs 2005;**50**:204-11.

40. Bradley EH, Webster TR, Schlesinger M, Baker D, Inouye SK. The roles of senior management in improving hospital experiences for frail older adults. J Healthc Manag 2006;**51**:323-36.

For peer review only

41. Caine C, Kenrick M. The role of clinical directorate managers in facilitating evidence-based practice: a report of an exploratory study. J Nurs Manag 1997;**5**:157-65.

42. Fox RT, Fox DH, Wells PJ. Performance of first-line management functions on productivity of hospital unit personnel. J Nurs Adm 1999;**29**:12-8.

43. Harris A. Risk management in practice: how are we managing? Clin Perform Qual Health Care 2000;**8**:142-9.

44. Saint S, Kowalski CP, Banaszak-Holl J, Forman J, Damschroder L, Krein SL. The importance of leadership in preventing healthcare-associated infection: Results of a multisite qualitative study. Infect Control Hosp Epidemiol 2010;**31**:901-7.

45. Poniatowski L, Stanley S, Youngberg B. Using information to empower nurse managers to become champions for patient safety. Nurs Adm Q 2005;**29**:72-7.

46. Balding C. Embedding organisational quality improvement through middle manager ownership. Int J Health Care Qual Assur 2005;**18**:271-88.

47. Bradley EH, Holmboe ES, Mattera JA, Roumanis SA, Radford MJ, Krumholz HM. The roles of senior management in quality improvement efforts: what are the key components? J Healthc Manag 2003;**48**:15-28.

48. Levey S, Vaughn T, Koepke M, Moore D, Lehrman W, Sinha S. Hospital Leadership and Quality Improvement: Rhetoric Versus Reality. J Patient Saf 2007;**3**:9-15.

49. Vaughn T, Koepke M, Kroch E, Lehrman W, Sinha S, Levey S. Engagement of Leadership in Quality Improvement Initiatives: Executive Quality Improvement Survey Results. J Patient Saf 2006;**2**:2-9.

50. Jha A, Epstein A. Hospital governance and the quality of care: Health Aff 2010;29:182-187.

51. Prybil LD, Peterson R, Brezinski P, Zamba G, Roach Jr W, Fillmore A. Board oversight of patient care quality in community health systems. Am J Med Qual 2010;**25**:34-41.

52. Braithwaite J, Finnegan TP, Graham EM, Degeling PJ, Hindle D, Westbrook MT. How important are quality and safety for clinician managers? Evidence from triangulated studies. Clin Governance 2004;**9**:34-41.

53. Weingart SN, Page D. Implications for practice: challenges for healthcare leaders in fostering patient safety. Qual Saf Health Care 2004;**13**:52-6.

54. Flin R, Maran N. Identifying and training non-technical skills for teams in acute medicine. Qual Saf Health Care 2004;**13**:80-4.

55. Kunzle B, Kolbe M, Grote G. Ensuring patient safety through effective leadership behaviour: A literature review. Safety Sci 2010;**48**:1-17.

56. Naranjo-Gil D. Strategic performance in hospitals: the use of the balanced scorecard by nurse managers. Health Care Manage Rev 2009;**34**:161-70.

57. Weiner BJ, Alexander JA, Shortell SM. Leadership for quality improvement in health care; empirical evidence on hospital boards, managers, and physicians. Med Care Res Rev 1996;**53**:397-416.

58. Edwards N, Marshall M, McLellan A, Abbasi K. Doctors and managers: A problem without a solution? BMJ 2003;**326**:609-10.

59. Wilkinson JE, Powell A, Davies H. Are clinicians engaged in quality improvement? A review of the literature on healthcare professionals' views on quality improvement initiative: Health Foundation Report; 2011 [accessed on 21.12.12]. Available from: http://www.health.org.uk/public/cms/75/76/313/2440/Are%20clinicians%20engaged%20in%20quality%20improvement.pdf?realName=y8NfS4.pdf.

For peer review only

60. Jha AK, Epstein AM. A Survey Of Board Chairs Of English Hospitals Shows Greater Attention To Quality Of Care Than Among Their US Counterparts. Health Aff 2013;**32**:677-85.

61. Stanton P. The role of an NHS Board in assuring the quality of clinically governed care and the duty of trust to patients. Clin Governance 2006;**11**:39-49.

62. Lukas CV, Holmes SK, Cohen AB, Restuccia J, Cramer IE, Shwartz M, et al. Transformational change in health care systems: an organizational model. Health Care Manage Rev 2007;**32**:309-20.

63. Shortell SM, Bennett CL, Byck GR. Assessing the impact of continuous quality improvement on clinical practice: what it will take to accelerate progress. The Milbank Q 1998;**76**:593-624, 510.

64. Keroack MA, Youngberg BJ, Cerese JL, Krsek C, Prellwitz LW, Trevelyan EW. Organizational factors associated with high performance in quality and safety in academic medical centers. Acad Med 2007;**82**:1178-86.

65. Parker VA, Wubbenhorst WH, Young GJ, Desai KR, Charns MP. Implementing quality improvement in hospitals: the role of leadership and culture. Am J Medical Qual 1999;**14**:64-9.

66. Weiner B, Shortell S, Alexander J. Promoting clinical involvement in hospital quality improvement efforts: the effects on top management, board, and physician leadership. Health Serv Res 1997;**32**:491-510.

67. Duckers MLA, Stegeman I, Spreeuwenberg P, Wagner C, Sanders K, Groenewegen PP. Consensus on the leadership of hospital CEOs and its impact on the participation of physicians in improvement projects. Health Policy 2009;**91**:306-13.

68. Vogus TJ, Sutcliffe KM. The impact of safety organizing, trusted leadership, and care pathways on reported medication errors in hospital nursing units. Med Care 2007;**45**:997-1002.

For peer review only

For peer review only - http://bmjopen.bmj.com/site/about/guidelines.xhtml

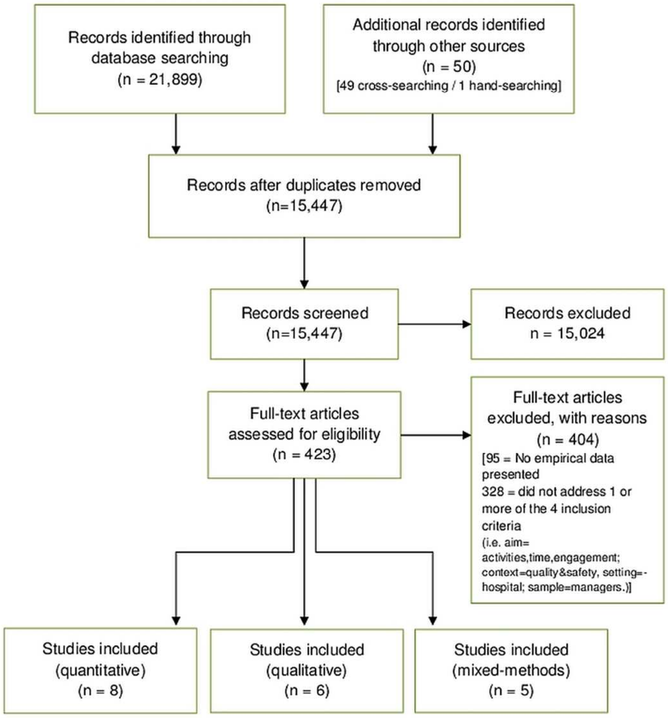

Figure 1 Review stages based on PRISMA Flow Diagram[33]
90x127mm (300 x 300 DPI)

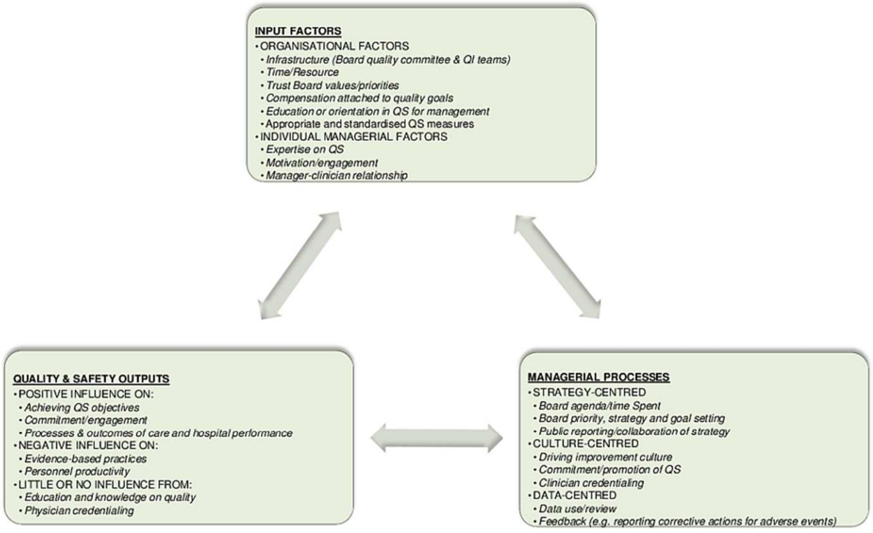

Figure 2 The Quality Management IPO model
90x63mm (300 x 300 DPI)

view only

APPENDICES TO APPEAR ONLINE

Appendix 1 Systematic review search strategies

Key: */=MeSH term; ti=title; ab=abstract; $=truncation; *=focus of search & substitution; exp=explode; adj=adjacent to*

MEDLINE SEARCH

Step	Search strategy formula
**1**	((safe$.ti,ab. or exp Safety/ or Err$.ti,ab. or Adverse.ti,ab.) and (exp *Risk Management/ or exp medical Errors/ or Safety Management/ or Medical Audit.mp.)) or exp Health Care Quality/ or patient centred care/ or length of stay/ or exp consumer satisfaction/ or patient readmission/ or exp evidence based medicine/ or exp "outcome and process assessment (health care)"/ or exp Quality Indicators, healthcare/ or quality assurance, health care/ or exp clinical audit/ or exp program evaluation/ or guideline adherence/ or (patient centred care or length of stay or customer satisfaction or patient satisfaction or medical audit or clinical audit or clinical effectiveness or performance measurement or outcome assessment or process assessment or guideline adherence or compliance to practice$1 or benchmarking or patient centred care or incident report$ or infection control or patient readmission or evidence based medicine or (evidence based adj2 practice) or waiting times or patient experience or complaints or target$1 or clinical excellence or service excellence or quality).ti,ab.
**2**	exp Hospital/ or (hospital$ or secondary care or acute care or health care organi*ation$1 or healthcare organi*ation$1 or infirmar$).ti,ab.
**3**	exp Health Facility Administrators/ or Governing board/ or (Manager$1 or Matron$1 or CEO$1 or executive$1 or director$3 or board$1 or middle management or senior management or lower management or frontline management or leader$4 or president$1 or head of department$1.ti,ab. or department head$1.ti,ab. or head of nursing or administrator$1 or healthcare administration or (chief adj4 officer$1) or (chief adj4 nurs$) or (chief adj4 operation$1) or (chief adj4 service$1) or chief of staff).ti,ab.
**4**	1 and 2 and 3
**5**	limit 4 to (human and English language and yr=”1983-Current”)

For peer review only - http://bmjopen.bmj.com/site/about/guidelines.xhtml

EMBASE SEARCH

Step	Search strategy formula
**1**	((exp Safety/ or Safe$.ti,ab. or Err$.ti,ab. or Adverse.ti,ab.) and (Health Care Quality.mp. or exp Health Care Organization/ or Health$.ti,ab.)) or exp Health Care Quality/ or clinical effectiveness/ or incident report/ or nursing outcome/ or performance measurement system/ or quality of nursing care/ or length of stay/ or hospital readmission/ or evidence based medicine/ or exp outcome assessment/ or quality control/ or medical audit/ or patient satisfaction/ or (patient centred care or length of stay or customer satisfaction or patient satisfaction or medical audit or clinical audit or clinical effectiveness or performance measurement or outcome assessment or process assessment or guideline adherence or compliance to practice$1 or benchmarking or patient centred care or incident report$ or infection control or patient readmission or evidence based medicine or (evidence based adj2 practice) or waiting times or patient experience or complaints or target$1 or clinical excellence or service excellence or quality).ti,ab.
**2**	exp Hospital/ or Health care organization/ or (hospital$ or secondary care or acute care or health care organi*ation$1 or healthcare organi*ation$1 or infirmar$).ti,ab.
**3**	exp manager/ or (Manager$1 or Matron$1 or CEO$1 or executive$1 or director$3 or board$1 or middle management or senior management or lower management or frontline management or leader$4 or president$1 or head of nursing or head of department$1.ti,ab. or department head$1.ti,ab. or administrator$1 or healthcare administration or health care administration or (chief adj4 officer$1) or (chief adj4 nurs$).ti,ab. or (chief adj4 operation$).ti,ab. or (chief adj4 service$).ti,ab. or chief of staff).ti,ab.
**4**	1 and 2 and 3
**5**	limit 4 to (human and English language and yr=”1983-Current”)

For peer review only - http://bmjopen.bmj.com/site/about/guidelines.xhtml

PSYCHINFO SEARCH

Step	Search strategy formula
**1**	((exp Safety/ or Safe$.ti,ab. or Err$.ti,ab. or Adverse.ti,ab.) and (Health Care Quality.mp. or risk management/ or Health$.ti,ab.)) or exp quality of care/ or quality control/ or Quality of Services/ or Treatment Duration/ or exp consumer satisfaction/ or exp client satisfaction/ or exp evidence based medicine/ or clinical audit/ or (patient centred care or length of stay or customer satisfaction or patient satisfaction or medical audit or clinical audit or clinical effectiveness or performance measurement or outcome assessment or process assessment or guideline adherence or compliance to practice$1 or benchmarking or patient centred care or incident report$ or infection control or patient readmission or evidence based medicine or (evidence based adj2 practice) or waiting times or patient experience or complaints or target$1 or clinical excellence or service excellence or quality).ti,ab.
**2**	Hospitals/ or (hospital$ or secondary care or acute care or health care organi*ation$1 or healthcare organi*ation$1 or infirmar$).ti,ab.
**3**	exp Management Personnel/ or exp Hospital Administration/ or (Manager$1 or Matron$1 or CEO$1 or executive$1 or director$3 or board$1 or middle management or senior management or lower management or frontline management or leader$4 or president$1 or head of department$1.ti,ab. or department head$1.ti,ab. or head of nursing or administrator$1 or healthcare administration or (chief adj4 officer$1) or (chief adj4 nurs$) or (chief adj4 operation$1) or (chief adj4 service$1) or chief of staff).ti,ab.
**4**	1 and 2 and 3
**5**	limit 4 to (human and English language and yr=”1983-Current”)

For peer review only

HMIC SEARCH

Step	Search strategy formula
**1**	((exp Safety/ or Safe$.ti,ab. or Err$.ti,ab. or Adverse.ti,ab.) and (Health Care Quality.mp. or Health$.ti,ab.)) or exp quality assurance in health services/ or patient centred care/ or hospital stay duration/ or exp consumer satisfaction/ or patient readmission/ or exp evidence based medicine/ or exp outcomes/ or benchmarking/ or (patient centred care or length of stay or customer satisfaction or patient satisfaction or medical audit or clinical audit or clinical effectiveness or performance measurement or outcome assessment or process assessment or guideline adherence or compliance to practice$1 or benchmarking or patient centred care or incident report$ or infection control or patient readmission or evidence based medicine or (evidence based adj2 practice) or quality improvement or waiting times or patient experience or complaints or target$1 or clinical excellence or service excellence or quality).ti,ab.
**2**	Acute Hospitals/ or hospital care/ or (hospital$ or secondary care or acute care or health care organi*ation$1 or healthcare organi*ation$1 or infirmar$).ti,ab.
**3**	exp managers/ or "middle and lower management"/ or senior managers/ or top management/ or (Manager$1 or Matron$1 or CEO$1 or executive$1 or director$3 or board$1 or middle management or senior management or lower management or frontline management or leader$4 or president$1 or head of department$1 or department head$1 or head of nursing or administrator$1 or healthcare administration or (chief adj4 officer$1) or (chief adj4 nurs$) or (chief adj4 operation$1) or (chief adj4 service$1) or chief of staff).ti,ab.
**4**	1 and 2 and 3
**5**	limit 4 to (human and English language and yr="1983-Current")

For peer review only - http://bmjopen.bmj.com/site/about/guidelines.xhtml

Appendix 2 Inclusion/exclusion criteria for systematic review

							Population sample		
	**Type of paper**	**Country**	**Language**	**Study design**	**Aims or focus**	**Setting**	**Population group reported on**	**Types of managers**	**Level of management**
**Inclusion**	Empirical Data	Any	English	Any (e.g. qualitative, quantitative or mixed methods)	To identify, describe or test types of managerial activities (i.e. behaviours, actions, role) relating to Q&S and QSI	Hospital	Hospital Managers	Clinical & operational managers (E.g. general managers, service managers, service delivery managers, directorate managers, Matrons, Ward managers, chief operating officer, chief executive officer or clinical directors or board managers or US administrators) Q&S managers: case managers, quality managers, risk managers, quality improvement managers *(mark as QM)*	Includes any levels of management (e.g. senior or middle management)
**Exclusion**	Commentary/opinion papers; literature reviews; editorials; conference proceedings; books guides; manuals; leaflets; conference proceedings; seminar proceedings; meeting notes; internal letters; Single case studies; and case studies that have no methods description.	None	Non-English	None	Managers' perceptions, knowledge, attitudes, awareness, competencies, leadership styles, or contextual issues (e.g. system barriers to involvement) of Q&S. Quality of cost of care.	Services solely for mental health or solely non-acute care community services; primary care; and all other industries	Managers from other settings; Senior clinicians (e.g. Clinician leaders such as consultants)	Those whose main position is to deal with financial management responsibilities alone<br><br>Other very specialist managers, e.g. materials specialist manager or information manager<br><br>Managers whose official role is quality and safety, e.g. quality manager	None

For peer review only - http://bmjopen.bmj.com/site/about/guidelines.xhtml

Appendix 3 Full review screening for systematic review

Article ID:

Stage 1

**A**	**Aim: Is the aim of the article to investigate work activities/engagement/time?**	**yes**	**no**	**unclear**
**B**	Context: Are the work activities in the context of Quality & Safety?	yes	no	unclear
**C**	Setting: Is the setting related to hospitals)?	yes	no	unclear
**D**	Sample Described: Are the work activities described those of managers?	yes	no	unclear

If no to any of the above then exclude and do not proceed to stage 2.
If yes to all, proceed to Stage 2.

**Proceed to stage 2**	**yes**	**no**

Stage 2

**Type: Is it an empirical study?**	**yes**	**no**	**unclear**

If yes, tick box Yes
If unclear, tick box Maybe
If no tick box Yes commentary

**Yes**	
**Maybe**	
**Yes commentary**	

Terms to identify the inclusion criteria

**A) ROLE**	Definition: Any activities carried out at work
	Key Terms: Role, tasks, work, responsibilities, activities, function, duties, undertaking, briefs, assignments, projects, errands, jobs, etc.
	Not: impact on quality & safety, managers competencies or knowledge on quality & safety etc
**B) QUALITY and/or SAFETY**	Definition: Safe, effective, patient-centred, timely efficient and equitable care.
	Key Terms Quality: Health Care Quality or length of stay or mortality or hospital readmission or exp evidence based medicine or evidence based practice or exp outcome assessment or quality control or medical audit or patient satisfaction or patient centred care or length of stay or mortality or customer satisfaction or patient readmission or evidence based medicine or or waiting times or patient experience or complaints or target(s) or clinical excellence or service excellence or quality or patient safety or medical errors or adverse events
	Not: Cost
**C) HOSPITALS**	Definition: Organisations that provide medical/surgical care
	Key Terms: Hospital(s) or acute care or acute care or health care organisation(s)
	Not: All other non-hospital settings, those that only focus on mental health & non-acute care community services, and all other industries
**D) MANAGEMENT**	Definition: A manager is a person that manages staff and has budgetary and service responsibilities. All levels of management are included (frontline managers, middle managers, senior managers, Board)
	Key Terms: General managers, service managers, service delivery managers, directorate managers, divisional managers, manager(s) or matron(s) or CEO(s) or executive(s) or director(s) or board(s) or middle management or senior management or leader(s) or president(s) or head(s) of nursing or head(s) of department(s) or department head(s) or administrator(s) or healthcare administration or chief officer(s) or chief(s) of staff, etc.
	Not: Clinician leaders such as consultants that do not hold budgetary responsibilities, those with financial management responsibilities, those with an official role in quality & safety (e.g. clinical governance managers, quality managers, risk managers)/

For peer review only - http://bmjopen.bmj.com/site/about/guidelines.xhtml

PRISMA 2009 Checklist

Section/topic	#	Checklist item	Reported on page #
**TITLE**			
Title	1	Identify the report as a systematic review, meta-analysis, or both.	1
**ABSTRACT**			
Structured summary	2	Provide a structured summary including, as applicable: background; objectives; data sources; study eligibility criteria, participants, and interventions; study appraisal and synthesis methods; results; limitations; conclusions and implications of key findings; systematic review registration number.	2
**INTRODUCTION**			
Rationale	3	Describe the rationale for the review in the context of what is already known.	4
Objectives	4	Provide an explicit statement of questions being addressed with reference to participants, interventions, comparisons, outcomes, and study design (PICOS).	NA (not a review of interventions)
**METHODS**			
Protocol and registration	5	Indicate if a review protocol exists, if and where it can be accessed (e.g., Web address), and, if available, provide registration information including registration number.	All protocol info in appendices
Eligibility criteria	6	Specify study characteristics (e.g., PICOS, length of follow-up) and report characteristics (e.g., years considered, language, publication status) used as criteria for eligibility, giving rationale.	7
Information sources	7	Describe all information sources (e.g., databases with dates of coverage, contact with study authors to identify additional studies) in the search and date last searched.	6
Search	8	Present full electronic search strategy for at least one database, including any limits used, such that it could be repeated.	in appendices
Study selection	9	State the process for selecting studies (i.e., screening, eligibility, included in systematic review, and, if applicable, included in the meta-analysis).	7
Data collection process	10	Describe method of data extraction from reports (e.g., piloted forms, independently, in duplicate) and any processes for obtaining and confirming data from investigators.	10
Data items	11	List and define all variables for which data were sought (e.g., PICOS, funding sources) and any assumptions and simplifications made.	6
Risk of bias in individual studies	12	Describe methods used for assessing risk of bias of individual studies (including specification of whether this was done at the study or outcome level), and how this information is to be used in any data synthesis.	10
Summary measures	13	State the principal summary measures (e.g., risk ratio, difference in means).	NA
Synthesis of results	14	Describe the methods of handling data and combining results of studies, if done, including measures of consistency (e.g., I^2) for each meta-analysis.	10

For peer review only - http://bmjopen.bmj.com/site/about/guidelines.xhtml

PRISMA 2009 Checklist

Section/topic	#	Checklist item	Reported on page #
Risk of bias across studies	15	Specify any assessment of risk of bias that may affect the cumulative evidence (e.g., publication bias, selective reporting within studies).	10
Additional analyses	16	Describe methods of additional analyses (e.g., sensitivity or subgroup analyses, meta-regression), if done, indicating which were pre-specified.	NA
**RESULTS**			
Study selection	17	Give numbers of studies screened, assessed for eligibility, and included in the review, with reasons for exclusions at each stage, ideally with a flow diagram.	9
Study characteristics	18	For each study, present characteristics for which data were extracted (e.g., study size, PICOS, follow-up period) and provide the citations.	16
Risk of bias within studies	19	Present data on risk of bias of each study and, if available, any outcome level assessment (see item 12).	16
Results of individual studies	20	For all outcomes considered (benefits or harms), present, for each study: (a) simple summary data for each intervention group (b) effect estimates and confidence intervals, ideally with a forest plot.	NA
Synthesis of results	21	Present results of each meta-analysis done, including confidence intervals and measures of consistency.	NA
Risk of bias across studies	22	Present results of any assessment of risk of bias across studies (see Item 15).	11
Additional analysis	23	Give results of additional analyses, if done (e.g., sensitivity or subgroup analyses, meta-regression [see Item 16]).	NA
**DISCUSSION**			
Summary of evidence	24	Summarize the main findings including the strength of evidence for each main outcome; consider their relevance to key groups (e.g., healthcare providers, users, and policy makers).	24
Limitations	25	Discuss limitations at study and outcome level (e.g., risk of bias), and at review-level (e.g., incomplete retrieval of identified research, reporting bias).	26
Conclusions	26	Provide a general interpretation of the results in the context of other evidence, and implications for future research.	27
**FUNDING**			
Funding	27	Describe sources of funding for the systematic review and other support (e.g., supply of data); role of funders for the systematic review.	30

From: Moher D, Liberati A, Tetzlaff J, Altman DG, The PRISMA Group (2009). Preferred Reporting Items for Systematic Reviews and Meta-Analyses: The PRISMA Statement. PLoS Med 6(6): e1000097. doi:10.1371/journal.pmed1000097

For more information, visit: **www.prisma-statement.org**.

For peer review only - http://bmjopen.bmj.com/site/about/guidelines.xhtml
